# The Tuberculosis Cascade of Care in India’s Public Sector: A Systematic Review and Meta-analysis

**DOI:** 10.1371/journal.pmed.1002149

**Published:** 2016-10-25

**Authors:** Ramnath Subbaraman, Ruvandhi R. Nathavitharana, Srinath Satyanarayana, Madhukar Pai, Beena E. Thomas, Vineet K. Chadha, Kiran Rade, Soumya Swaminathan, Kenneth H. Mayer

**Affiliations:** 1 Division of Infectious Diseases, Brigham and Women’s Hospital and Harvard Medical School, Boston, Massachusetts, United States of America; 2 Partners for Urban Knowledge, Action, and Research (PUKAR), Mumbai, India; 3 Division of Infectious Diseases, Beth Israel Deaconess Medical Center and Harvard Medical School, Boston, Massachusetts, United States of America; 4 Section of Infectious Diseases & Immunity, Imperial College London, London, United Kingdom; 5 Department of Epidemiology, Biostatistics and Occupational Health and McGill International TB Centre, McGill University, Montreal, Canada; 6 Center for Operations Research, International Union Against Tuberculosis and Lung Disease, Paris, France; 7 Department of Social and Behavioral Research, National Institute for Research in Tuberculosis, Chennai, India; 8 Epidemiology and Research Division, National Tuberculosis Institute, Bangalore, India; 9 World Health Organization, Country Office for India (RNTCP-TSN), New Delhi, India; 10 Indian Council of Medical Research, New Delhi, India; 11 The Fenway Institute, Boston, Massachusetts, United States of America; Harvard School of Public Health, UNITED STATES

## Abstract

**Background:**

India has 23% of the global burden of active tuberculosis (TB) patients and 27% of the world’s “missing” patients, which includes those who may not have received effective TB care and could potentially spread TB to others. The “cascade of care” is a useful model for visualizing deficiencies in case detection and retention in care, in order to prioritize interventions.

**Methods and Findings:**

The care cascade constructed in this paper focuses on the Revised National TB Control Programme (RNTCP), which treats about half of India’s TB patients. We define the TB cascade as including the following patient populations: total prevalent active TB patients in India, TB patients who reach and undergo evaluation at RNTCP diagnostic facilities, patients successfully diagnosed with TB, patients who start treatment, patients retained to treatment completion, and patients who achieve 1-y recurrence-free survival. We estimate each step of the cascade for 2013 using data from two World Health Organization (WHO) reports (2014–2015), one WHO dataset (2015), and three RNTCP reports (2014–2016). In addition, we conduct three targeted systematic reviews of the scientific literature to identify 39 unique articles published from 2000–2015 that provide additional data on five indicators that help estimate different steps of the TB cascade. We construct separate care cascades for the overall population of patients with active TB and for patients with specific forms of TB—including new smear-positive, new smear-negative, retreatment smear-positive, and multidrug-resistant (MDR) TB.

The WHO estimated that there were 2,700,000 (95%CI: 1,800,000–3,800,000) prevalent TB patients in India in 2013. Of these patients, we estimate that 1,938,027 (72%) TB patients were evaluated at RNTCP facilities; 1,629,906 (60%) were successfully diagnosed; 1,417,838 (53%) got registered for treatment; 1,221,764 (45%) completed treatment; and 1,049,237 (95%CI: 1,008,775–1,083,243), or 39%, of 2,700,000 TB patients achieved the optimal outcome of 1-y recurrence-free survival.

The separate cascades for different forms of TB highlight different patterns of patient attrition. Pretreatment loss to follow-up of diagnosed patients and post-treatment TB recurrence were major points of attrition in the new smear-positive TB cascade. In the new smear-negative and MDR TB cascades, a substantial proportion of patients who were evaluated at RNTCP diagnostic facilities were not successfully diagnosed. Retreatment smear-positive and MDR TB patients had poorer treatment outcomes than the general TB population. Limitations of our analysis include the lack of available data on the cascade of care in the private sector and substantial uncertainty regarding the 1-y period prevalence of TB in India.

**Conclusions:**

Increasing case detection is critical to improving outcomes in India’s TB cascade of care, especially for smear-negative and MDR TB patients. For new smear-positive patients, pretreatment loss to follow-up and post-treatment TB recurrence are considerable points of attrition that may contribute to ongoing TB transmission. Future multisite studies providing more accurate information on key steps in the public sector TB cascade and extension of this analysis to private sector patients may help to better target interventions and resources for TB control in India.

## Abstract 2

In this systematic review and meta-analysis of public sector health data, Ramnath Subbaraman and colleagues estimate how many tuberculosis patients are "lost" and therefore do not receive effective care at each step of the treatment cascade.

## Author Summary

**Why was this study done?:**

- India is the country with the highest burden of tuberculosis (TB) in the world, with one-quarter of the world’s patients with active TB disease.
- The World Health Organization (WHO) estimates that India has nearly 1 million “missing” TB patients, who have not been reported to the national TB program and who therefore may not have received effective TB care.
- Government reports and studies from local regions of India suggest that a considerable percentage of TB patients are evaluated at government health facilities but fail to be diagnosed with TB or fail to start TB treatment even if they are correctly diagnosed, but these reports and studies have not been collectively analyzed to provide national estimates of critical points at which patients are being “lost” from the government TB program.
- The purpose of our study is to estimate how many TB patients in India’s national TB program are not being detected, not enrolling in treatment, not completing treatment, and not surviving without TB recurrence for 1 y after finishing treatment, using a model called the “cascade of care.”

**What did the researchers do and find?:**

- To estimate different steps of the TB cascade of care in India in 2013, we collected data from multiple official reports published by the WHO and India’s national TB program.
- We also conducted three systematic searches of the medical literature to identify 39 studies published between 2000–2015 that describe patient loss to follow-up at different steps of India’s TB cascade; we synthesized some of these findings using an approach called meta-analysis.
- We estimate that, of about 2,700,000 prevalent TB patients in India in 2013, 1,938,027 (72%) were evaluated at government TB health facilities; 1,629,906 (60%) were successfully diagnosed with TB; 1,417,838 (53%) started TB treatment; 1,221,764 (45%) completed TB treatment; and about 1,049,237 (39%) achieved the optimal outcome of 1-y recurrence-free survival.
- Patients who had a history of TB in the past (also called retreatment patients) and patients with TB resistant to the two most effective medications (also called multidrug-resistant TB) have considerably worse outcomes compared to other TB patients.
- The critical points at which patients are being “lost” to the government system varies depending on the type of TB that a patient has.

**What do these findings mean?:**

- Our findings suggest that, for some forms of TB—such as smear-negative TB and multidrug-resistant TB—increasing detection and diagnosis of new patients by using new TB diagnostic tests may be the most important intervention for improving patient outcomes.
- For other types of TB patients—such as new smear-positive patients—reducing loss to follow-up immediately after TB is diagnosed and improving adherence to medications so that TB is less likely to recur might be the best interventions to improve patient outcomes.
- Since a considerable proportion of patients who complete TB therapy experience recurrence of TB, routine follow-up of all patients for 1 y after completion of TB therapy may be an efficient approach for identifying new TB patients.
- Our study is limited by the fact that very little information is available regarding treatment outcomes for TB patients receiving care in the private sector and by considerable uncertainty in the number of prevalent TB patients in India.
- We recommend that well-designed research be conducted at multiple sites in India’s national TB program and in the private sector to improve the accuracy of the TB cascade in the future and to monitor progress on TB control.

## Introduction

India has the world’s largest tuberculosis (TB) epidemic, with 23% of the global burden of incident active TB patients annually and 27% of the world’s “missing” patients, representing about 1 million patients each year who have not been notified to the Government of India’s Revised National TB Control Programme (RNTCP) [1,2]. These patients may not have received health services for TB or may have received potentially suboptimal TB care in India’s private sector [3,4]. In recent years, the RNTCP has changed its policy objective from successfully identifying 70% of new smear-positive patients and completing treatment for 85% of these patients to a broader goal of “universal access to quality care” for all TB patients [5]. Similarly, the World Health Organization (WHO)’s post-2015 “End TB” Strategy hopes to treat “all people with TB, including drug-resistant TB” and to end the global TB epidemic by 2035 [6].

Achieving such an ambitious objective in India will require improvements across various RNTCP activities—including access to services, case finding, linkage of diagnosed patients to treatment, retention in treatment, and coordination with private sector providers—to bring about successful outcomes of treatment completion and, ideally, TB recurrence-free survival for all patients.

The “cascade of care” is a useful model for evaluating care delivery by a health system for a specific disease. This model has been used most extensively to assess the adequacy of HIV care [7,8] but has also been used for other diseases, including type II diabetes and hepatitis C [9,10]. The model defines an outcome that all patients should achieve—for example, virological suppression in the case of HIV. It outlines a series of sequential steps needed to achieve that outcome. Estimates are provided of the number of patients who have successfully achieved each sequential step. In the field of HIV, this model is sometimes referred to as the “continuum of care,” reflecting the idea that, because HIV is a chronic disease, patients can sometimes move “backwards” along these steps during their life course (e.g., dropping out of care after initially being retained in care or developing HIV viremia after initially achieving viral suppression). Since TB is usually not a lifelong disease and can be cured with appropriate therapy, we use the term “cascade” to describe our model, even though TB patients can occasionally also move “backwards” in care along the continuum.

The strength of the cascade model is that it allows policymakers to visualize the biggest gaps in care delivery. They can then identify patient subpopulations who may be at highest risk for adverse outcomes or who may drive the development of incident TB patients. The cascade model may promote more efficient targeting of resources and motivate development of new strategies for addressing shortfalls in care. While the WHO has promoted a similar “onion” model (i.e., assessment of concentric layers of patient attrition) for evaluating local TB programs [11], to date, neither the onion nor the cascade model has been used to understand gaps in care delivery for TB at a national scale.

In this article, we describe the TB cascade of care in the public sector in India for 2013, based on an integrated model that draws from the WHO’s onion model and the cascade model used for HIV. Although about half of India’s TB patients may receive private sector care [12,13], we focus on the public sector alone, given substantial limitations in data on TB outcomes in the private sector. Our primary objective is to estimate the number of patients completing each step of the cascade, and an important secondary objective is to highlight gaps in knowledge in order to guide future research to improve the cascade’s accuracy. We also describe cascades for new smear-positive, retreatment smear-positive, new smear-negative, and multidrug-resistant (MDR) TB patients, to show how this model may shed light on care delivery for specific patient subpopulations.

## Methods

### Integrating the Cascade Model with the WHO Onion Model

The WHO onion model serves as an excellent starting point for constructing a TB cascade of care; however, this model has a few deficiencies [11]. The WHO model has been primarily promoted as a method for refining estimates of TB incidence. As a result, the stopping point for this model is a programmatic outcome—the total number of TB patients notified to the government—rather than a patient-oriented outcome, such as cure, treatment completion, or recurrence-free survival.

We believe that 1-y TB recurrence-free survival is a useful end outcome of the cascade. This indicator not only reflects whether patients completed treatment but also whether treatment was of high quality, since poor adherence increases the risk of post-treatment TB relapse or death [14]. We assume that most patients with recurrent TB in the RNTCP are experiencing disease relapse (rather than reinfection). Two studies help to justify this assumption. First, a study of TB recurrence in South India that used genotypic techniques to compare the DNA fingerprints of paired mycobacterial isolates from patients at the time of initial diagnosis and the time of TB recurrence found that recurrence was due to exogenous re-infection in only 9% of HIV-uninfected patients as compared to 88% of HIV-infected patients [15]. Since the prevalence of HIV-infection in the RNTCP’s overall population of TB patients is low (about 6%) [16], this suggests that the vast majority of TB recurrence represents relapse of prior disease. Second, another study in the RNTCP found that patients who took TB therapy irregularly were twice as likely to experience TB recurrence, even after adjusting for other risk factors, suggesting that the TB recurrence rate partly reflects medication adherence and the quality of care received by patients [14].

We use a 1-y minimum time period after treatment completion to assess for recurrence-free survival because most TB relapses (91%) occur within 1 y [17]. One-year post-treatment follow-up is recommended by the Standards for TB Care in India [18], and routine 1-y follow-up of all patients may be more feasible for national TB programs than longer follow-up periods.

The WHO onion model focuses on estimating “gaps” (the proportion of patients who did not complete specific steps), while most HIV cascades estimate the absolute number of patients who reach each step [7]. In most cascade models, the “gaps” are implied by each drop-off in the bar graph.

**Fig 1:**
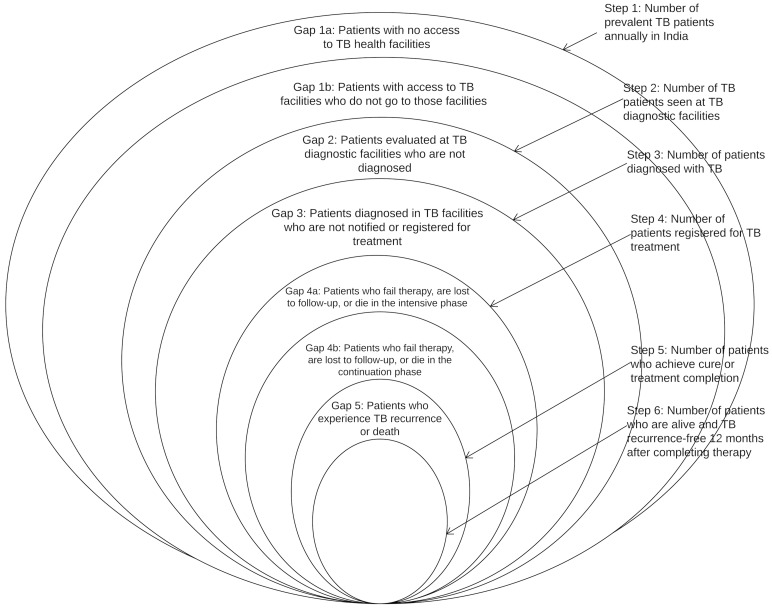
A model for the tuberculosis cascade of care in India that integrates the WHO onion model with concepts from the HIV cascade of care. Adapted from WHO, 2009 [11].

We have therefore integrated the WHO onion model and the cascade model, as represented in Fig 1. Each ring represents the estimated number of TB patients at that step of the cascade; we will refer to each of these rings as a “step.” This integrated model incorporates the following steps: (1) the total number of prevalent active TB patients in India, (2) the number of TB patients who reached and were evaluated at TB diagnostic facilities, (3) the number of patients diagnosed with TB, (4) the number of TB patients registered for treatment, (5) the number of patients who achieved cure or treatment completion, and (6) the number of patients who remained alive and TB recurrence-free as assessed a minimum of 1 y (and no more than 24 mo) after completing therapy. For steps 2–6, we will only include data from public sector RNTCP facilities in our analysis, as discussed further below. The spaces between the rings represent the proportion of patients who fail to complete each step; we will refer to these spaces as “gaps.”

The steps and gaps in the model can also be divided into the following parts: accessing care (Steps 1/2, Gaps 1a/1b/2), diagnosis and treatment registration (Steps 3/4, Gap 3), retention during the treatment course (Step 5, Gaps 4a/4b), and recurrence-free survival (Step 6, Gap 5). The steps and gaps are interrelated; in some cases we use data on steps to help estimate subsequent gaps and vice versa.

### Data Sources

Detailed information on the data sources and methods used to estimate cascade steps and gaps are presented in Tables 1 and 2. The online database associated with WHO’s 2015 Global TB Report provides the most recently revised estimate of the total number of prevalent TB patients in India in 2013 [2,19]. The 2014 WHO Global TB Report provides an estimate of the total number of multidrug-resistant tuberculosis (MDR TB) patients among notified TB patients in the RNTCP in 2013 [1]. The RNTCP publishes national statistics annually in a document called *TB India*: *RNTCP annual status report* [16,20,21]. This report contains statistics on the total number of TB patients registered for treatment in the government program.

**Table 1:** Methods and data sources used to estimate each step of the TB cascade of care for different subpopulations of patients and for the overall population of TB patients in India in 2013.

	Step 1 (total prevalent patients)	Step 2 (reached and evaluated at TB diagnostic facilities)	Step 3 (diagnosed with TB)	Step 4 (registered in treatment)	Step 5 (completed treatment)	Step 6 (achieved recurrence-free survival at 12–24 mo)
**New smear-positive**	No data available	Back-calculation from Gap 2	“Number of smear positive patients diagnosed” from *TB India* report [16]	*TB India* report [20]	*TB India* report [20]	Calculation from Gap 5
**New smear-negative**	No data available	Back-calculation from Gap 2	Back-calculation from Gap 3	*TB India* report [20]	*TB India* report [20]	Calculation from Gap 5
**Extrapulmonary TB**	No data available	Back-calculation from Gap 2	Back-calculation from Gap 3	*TB India* report [20]	*TB India* report [20]	Calculation from Gap 5
**Retreatment smear-positive**	No data available	Back-calculation from Gap 2	“Number of smear positive patients diagnosed” from *TB India* report [16]	*TB India* report [20]	*TB India* report [20]	Calculation from Gap 5
**Retreatment smear-negative**	No data available	Back-calculation from Gap 2	Back-calculation from Gap 3	*TB India* report [20]	Study on treatment outcomes of retreatment smear-negative patients [22]	Calculation from Gap 5
**Multidrug-resistant**	No data available	WHO Global TB Report estimate of MDR TB patients among all notified TB patients [1]	Back-calculation from Gap 3	*TB India* report [21]	*TB India* report [21]	Calculation from Gap 5
**All forms of TB**	WHO Global TB Report [2,19]	Addition of the above estimates for each form of TB	Addition of the above estimates for each form of TB	Addition of the above estimates for each form of TB	Addition of the above estimates for each form of TB	Addition of the above estimates for each form of TB

**Table 2:** Methods and data sources used to estimate each gap of the TB cascade of care for different subpopulations of patients and for the overall population of TB patients in India in 2013.

	Gap 1a (no access to TB facilities)	Gap 1b (access to TB facilities but did not seek care)	Gap 2 (evaluated at TB diagnostic facilities but not diagnosed with TB)	Gap 3 (diagnosed with TB but not registered in treatment)	Gaps 4a/4b (treatment failure, death, or loss to follow-up on treatment)	Gap 5 (TB recurrence or death in the 12–24 mo after treatment completion)
**New smear-positive**	No data available	Meta-analysis of studies of care-seeking by chest symptomatics^a^	Meta-analysis of studies to estimate the proportion of patients who do not submit a second sputum sample	Difference between Steps 3 and 4; cross-checked by meta-analysis of studies of pretreatment loss to follow-up of smear-positive patients	Difference between Step 4 and 5	Pooled prevalence of studies estimating TB recurrence and death of new smear-positive patients after completing Category I therapy
**New smear-negative**	No data available	Meta-analysis of studies of care-seeking by chest symptomatics^a^	Estimated using ratio of smear-positive to smear-negative patients derived from a multisite study of Xpert MTB/Rif [24]	Conservative estimate of 10.5% pretreatment loss extrapolated from the proportion lost after referral to directly observed therapy centers for smear positive patients	Difference between Step 4 and 5	Pooled prevalence of studies estimating TB recurrence and death of new smear-negative, new smear-positive, and extrapulmonary patients after completing Category I or III therapy
**Extrapulmonary TB**	No data available	No data available	Estimated as the mean of Gap 2 for new smear-positive and new smear-negative patients as no data are available on this gap for extrapulmonary TB	Same estimate used for new smear-negative patients	Difference between Step 4 and 5	Pooled prevalence of studies estimating TB recurrence and death of new smear-negative, new smear-positive, and extrapulmonary patients after completing Category I or III therapy
**Retreatment smear-positive**	No data available	No data available	Meta-analysis of studies to estimate the proportion of patients who do not submit a second sputum sample	Difference between Steps 3 and 4; cross-checked by meta-analysis of studies of pretreatment loss to follow-up of smear-positive patients	Difference between Step 4 and 5	Estimate derived from one study of TB recurrence among retreatment smear-positive patients [25]
**Retreatment smear-negative**	No data available	No data available	Estimated using ratio of smear-positive to smear-negative patients derived from a multisite study of Xpert MTB/Rif [24]	Same estimate used for new smear-negative patients	Difference between Step 4 and 5	Same estimate as used for new smear-negative patients
**Multidrug-resistant (MDR)**	No data available	No data available	Difference of the WHO estimate of MDR TB patients among all notified pulmonary TB patients (Step 2) [1] and the total number of diagnosed MDR TB patients extrapolated from RNTCP data (Step 3) [21]	Pooled prevalence based on two studies of pretreatment loss of MDR TB patients	Difference between Step 4 and 5	Same estimate used for retreatment smear-positive patients; given poor outcomes of MDR TB patients, we assume this estimate is conservative
**All forms of TB**	Gap 1a/1b for the overall cascade is estimated as the difference between Step 1 and Step 2		Addition of the above estimates for each form of TB	Addition of the above estimates for each form of TB	Addition of the above estimates for each form of TB	Addition of the above estimates for each form of TB

^a^Note that these studies provide general insights into Gap 1b (the proportion of patients who do not seek care); however, they are not used to formally estimate this gap.

Treatment outcomes are provided in the *TB India* reports for all categories of TB, except retreatment smear-negative patients [20]. Due to the long duration of treatment, final outcomes for MDR TB patients are not reported until 3 y after treatment initiation. Therefore, 2013 is the earliest year for which we are able to construct a TB cascade of care that includes patients with all forms of TB, since treatment outcomes for MDR TB patients registered in 2013 were published in the 2016 *TB India* report [21].

We conducted targeted systematic reviews and meta-analyses of Indian studies published from 2000 to 2015 to inform or estimate the following gaps in the TB cascade of care: (1) the proportion of individuals in the community with cough >2 wk (i.e., suspected TB patients or “chest symptomatics”) who have not sought medical care (Gap 1b); (2) the proportion of patients who provide one sputum smear at designated microscopy centers but who fail to provide a second smear (sometimes referred to as “lost to follow-up during diagnosis” or “diagnostic default,” used to estimate Gap 2) [23]; (3) the proportion of patients who are lost to follow-up during the multistep diagnostic workup for smear-negative TB (Gap 2); (4) the proportion of smear-positive patients diagnosed at microscopy centers who fail to register for treatment (also known as “pretreatment loss to follow-up” or “initial default,” used to estimate Gap 3); and (5) the proportion of TB patients who experience TB recurrence or death within 12–24 mo after treatment completion (Gap 5). The PRISMA checklist and methods for each of these systematic reviews are presented in detail in S1–S4 Texts.

### Analytical Approach

We organize TB patients registered in the RNTCP into the following categories: (1) new smear-positive, (2) new smear-negative, (3) new extrapulmonary, (4) retreatment smear-positive, (5) retreatment smear-negative, and (6) multidrug-resistant (MDR). We define “retreatment smear-positive” patients as including patients falling into the categories of treatment after failure (TAF), treatment after default (TAD), and smear-positive relapse (i.e., patients with TB recurrence) in the *TB India* reports. We define retreatment smear-negative patients as consisting of patients listed in the category of “retreatment other” in the *TB India* reports, based on a recent study which found that the vast majority of “retreatment other” patients consist of smear-negative patients with a history of prior TB [22]. For some forms of TB, no data were available from the WHO reports, the *TB India* reports, or the published literature to estimate a few of the gaps in the cascade. When reasonable, we extrapolated from estimates of the same gap for other forms of TB. In general, we made conservative assumptions that favor better outcomes for the health system.

In 2012, the Government of India issued an order for mandatory private sector notification of TB patients facilitated by a web-based application called E-NIKSHAY. Private sector participation in E-NIKSHAY is low but slowly gaining momentum [26,27]; our study therefore reflects a time period before a substantial increase in private sector notifications, when they had been historically very low. Only 38,596 TB patients were notified to the government by the private sector in 2013 [16], and, since treatment outcome data for these patients are not available, we exclude them from the cascade.

For many of the steps and gaps, uncertainty in the estimated number of patients has been represented using 95% confidence intervals (95%CIs) when appropriate. Most of these 95%CIs derive from the meta-analysis or pooled prevalence estimates that inform specific cascade steps, based on studies identified through the systematic reviews. When appropriate, we have been careful to propagate uncertainty in situations in which we were calculating a chain of estimates involving multiple proportions or numerical estimates, each with its own confidence interval. In such cases, we take the uncertainty of both values into account by multiplying the upper and lower bounds of the confidence interval of the first value by the upper and lower bounds of the confidence interval of the second value in a manner that produces the widest possible confidence interval for the new value.

In addition, when calculating the “gaps” in the cascade, we subtract the upper bound of the confidence interval for a step from the lower bound of the confidence interval for the preceding step to estimate the most conservative lower bound of the confidence interval for the gap between those steps. Similarly, we subtract the lower bound of the confidence interval for a step from the upper bound of the confidence interval for the preceding step to estimate the most conservative upper bound of the confidence interval for the gap between those steps. The sources of uncertainty for the estimates of specific steps in the TB cascade of care are summarized in Table 3.

**Table 3:** Sources of uncertainty for each step in the TB cascade of care in India in 2013.

	Step 1 (total prevalent patients)	Step 2 (reached and evaluated at TB diagnostic facilities)	Step 3 (diagnosed with TB)	Step 4 (registered in TB treatment)	Step 5 (completed TB treatment)	Step 6 (achieved TB recurrence-free survival at 12–24 mo)
**New smear-positive**	--	Confidence intervals from meta-analysis of the proportion of patients who fail to provide a second sputum specimen	Assumption that pretreatment loss to follow-up is equal for NSP and RSP patients^b^	Precise value from *TB India* report [20]	Precise value from *TB India* report [20]	Confidence interval for pooled prevalence of smear-positive TB recurrence
**New smear-negative**	--	Confidence intervals for: (a) sensitivity of Xpert for diagnosing smear-negative TB [30]; (b) assumption of infinite population size for smear-positive to smear-negative ratio; (c) number of new smear-positive patients evaluated (Step 2)^a^	Confidence interval for the pretreatment loss to follow-up estimate for smear-negative and extrapulmonary TB patients	Precise value from *TB India* report [20]	Precise value from *TB India* report [20]	Confidence interval for pooled prevalence of smear-negative and extrapulmonary TB recurrence
**Extrapulmonary TB**	--	Confidence intervals for: (a) proportion of undiagnosed smear-positive patients (Gap 2); (b) proportion of undiagnosed smear-negative patients (Gap 2); (c) number of extrapulmonary TB patients diagnosed (Step 3)^a^	Confidence interval for the pretreatment loss to follow-up estimate for smear-negative and extrapulmonary TB patients	Precise value from *TB India* report [20]	Precise value from *TB India* report [20]	Confidence interval for pooled prevalence of smear-negative and extrapulmonary TB recurrence
**Retreatment smear-positive**	--	Same sources of uncertainty as for Step 2 for new smear-positive patients	Assumption that pretreatment loss to follow-up is equal for NSP and RSP patients^b^	Precise value from *TB India* report [20]	Precise value from *TB India* report [20]	Confidence interval for estimate of retreatment smear-positive TB recurrence [25]
**Retreatment smear-negative**	--	Confidence intervals for: (a) sensitivity of Xpert for diagnosing smear-negative TB [30]; (b) assumption of infinite population size for smear-positive to smear-negative ratio; (c) number of retreatment smear-positive patients evaluated (Step 2)^a^	Confidence interval for the pretreatment loss to follow-up estimate for smear-negative and extrapulmonary TB patients	Precise value from *TB India* report [20]	Confidence interval for estimated treatment outcomes of retreatment “others” [22]	Confidence interval for pooled prevalence of smear-negative and extrapulmonary TB recurrence with propagated uncertainty from Step 5 estimate^a^
**Multidrug-resistant**	--	Confidence intervals for MDR TB patients among notified TB patients from the WHO Global TB Report [1]	Confidence interval for pretreatment loss to follow-up estimate for MDR TB patients	Precise value from *TB India* report [21]	Precise value from *TB India* report [21]	Confidence interval for estimate of retreatment smear-positive TB recurrence [25]
**All forms of TB**	Confidence intervals for TB prevalence from the WHO TB Report [2,19]	Overall confidence intervals estimated by separately combining all upper bounds and lower bounds of the confidence intervals for each form of TB	Overall confidence intervals estimated by separately combining all upper bounds and lower bounds of the confidence intervals for each form of TB	No confidence interval as estimates for each form of TB are precise values from the *TB India* reports	Confidence interval for retreatment smear-negative Step 3 estimate; all other forms of TB have precise estimates	Overall confidence intervals estimated by separately combining all upper bounds and lower bounds of the confidence intervals for each form of TB

^a^Steps with multiple reasons for uncertainty with different confidence intervals, and these different types of uncertainty are “propagated” in our estimates

^b^Types of uncertainty for which we are unable to provide confidence intervals in this model

### Estimating Steps and Gaps in the TB Cascade of Care

Detailed explanations of the assumptions and calculations for every step and gap in the TB cascade of care are included in S5 Text. We summarize the methods used to estimate every step and gap below.

#### Accessing care

Step 1: We used the revised 2013 WHO prevalence estimate of the overall number of TB patients in India as the starting step of the cascade [2,19] because TB prevalence is estimated partly from population-based surveys. In contrast, estimates of incidence may be more uncertain because they are extrapolated from the number of TB patients notified to the government [28]. Also, TB incidence estimates exclude some types of retreatment TB patients (specifically “failure” and “treatment after default”); these types of TB are relevant to the overall cascade, since such patients require ongoing or modified treatment and may continue to transmit TB.

Gap 1a: The proportion of prevalent TB patients with no access to government TB facilities is difficult to estimate based on existing data. As per the RNTCP, universal district-level coverage with directly observed therapy (DOT) centers was achieved in 2006 [29]; however, recent studies suggest that some subpopulations, especially remote Adivasi (“tribal”) populations, continue to have poor access to government TB services [31]. Unfortunately, national-level data are not available to help quantify this gap, so we are not able to separate Gap 1a from Gap 1b. As a result, Gaps 1a and 1b are presented as a single gap in the cascade. We assume that Gaps 1a and 1b can be estimated together as the difference between Step 1 (total prevalent patients) and Step 2 (the number of TB patients seen at diagnostic facilities) (S5 Text).

Gap 1b: The proportion of patients with access to government TB services who never seek care at these facilities (and remain without care or in private sector care) is also difficult to estimate using existing data. However, there are multiple population-based studies assessing the proportion of people with cough >2–3 wk (i.e., people with suspected TB or “chest symptomatics”) who had not sought medical evaluation at the time of the survey.

This indicator has limitations, since the behavior of chest symptomatics may not fully reflect the behavior of patients who actually have TB, because chronic cough could be secondary to other causes, including chronic obstructive pulmonary disease or other chronic infections. About 5%–10% of patients in India with chronic cough screened at government microscopy facilities and about 2%–5% in community surveys have a positive sputum specimen for TB, which suggests that cough for >2 wk has relatively low positive predictive value for TB [20,32–36]. Screening for cough is also not completely sensitive for pulmonary TB, as a minority of pulmonary TB patients do not have cough but instead have other symptoms such as chest pain, hemoptysis, or fever. In addition, not having sought care at the time of the survey does not necessarily mean that these individuals with suspected TB will never seek care.

Despite these limitations, we systematically reviewed and meta-analyzed studies of care-seeking by chest symptomatics (S2 Text), because these findings still provide some value in understanding TB care-seeking behavior at the population level. However, we did not use the findings of this meta-analysis to calculate Gap 1b. Instead, Gaps 1a and 1b were estimated as the difference between Steps 1 and 2 as noted above (S5 Text).

Step 2 and Gap 2: We estimated Step 2 (the number of TB patients who reach and are evaluated at RNTCP diagnostic facilities) for all forms of TB except MDR TB through back-calculation from estimates for Step 3 (the number of TB patients who are successfully diagnosed). For all forms of TB, we then estimated Gap 2 (the number of patients evaluated at TB diagnostic facilities who remain undiagnosed) as the difference between the estimates for Step 2 and Step 3 (S5 Text).

To estimate Step 2 for new and retreatment smear-positive patients, we assumed that these patients may remain undiagnosed despite being initially evaluated at a diagnostic microscopy center if they are lost to follow-up before providing a second sputum specimen. We therefore estimated the proportion of patients who fail to provide a second sputum specimen through a meta-analysis of previous studies in India (Table F in S3 Text, and Results section, below). We then estimated the number of new and retreatment smear-positive patients who might have remained undiagnosed because they did not provide a second sputum specimen, by extrapolating from findings of a previously published meta-analysis that estimated a 11.9% incremental diagnostic yield of a second sputum specimen for diagnosing smear-positive TB (confidence intervals for this value are not provided in that manuscript) [37].

Based on these two values, we estimated that 1.3% (95%CI: 0.8%–1.8%) of smear-positive patients presenting to TB diagnostic facilities may remain undiagnosed because some patients fail to provide a second sputum specimen for diagnostic evaluation. Using this proportion for Gap 2, we back-calculated an estimate for Step 2 from Step 3 for both new smear-positive and retreatment smear-positive patients (S5 Text).

We estimated Step 2 for new smear-negative and retreatment smear-negative patients as follows: There are multiple reasons why smear-negative TB patients may remain undiagnosed despite presenting to a government TB diagnostic facility. Due to lack of mycobacterial culture and polymerase chain reaction (PCR)-based diagnostic tests such as Xpert MTB/RIF at most facilities, smear-negative TB patients are diagnosed empirically using a multistep workup that includes a trial of broad-spectrum antibiotics and a chest X-ray (Fig B in S3 Text). Studies identified in our systematic review highlight dramatic attrition of smear-negative TB suspects during each step of this workup (Table G in S3 Text) [38–40]; however, none of these studies evaluated this diagnostic algorithm against a gold standard diagnosis of smear-negative TB using mycobacterial culture. Therefore, it is unclear what proportion of “true” smear-negative TB patients fail to get diagnosed.

We therefore used an alternative approach to estimate Step 2 for smear-negative patients. Historical data suggest that the proportion of new smear-positive to new smear-negative TB patients is 1:1 [41]; the RNTCP also cites this ratio in its training manual [42]. However, we estimate a more conservative 1.42:1 (95%CI: 1.26:1 to 1.59:1) ratio of smear-positive to smear-negative TB patients, based on data from the recent study evaluating the implementation of Xpert at 18 sites in India (S5 Text) [24].

Using this ratio, we back-calculated the expected number of new smear-negative TB patients being evaluated at diagnostic microscopy facilities (Step 2) from the estimated number of new smear-positive patients being evaluated at these facilities (Step 2) (S5 Text). We also used this ratio to back-calculate the number of retreatment smear-negative patients in Step 2 from the number of retreatment smear-positive patients in Step 2.

To estimate Step 2 for extrapulmonary TB patients, there are no studies we could identify that estimate the proportion of extrapulmonary patients evaluated at RNTCP diagnostic centers who fail to get appropriately diagnosed. One local study suggests that the substantial majority of extrapulmonary TB in India’s public sector is being diagnosed clinically without collection of diagnostic samples for mycobacterial stain, culture, or histopathology [43]. Of the most common forms of extrapulmonary TB, lymphadenitis is relatively easy to diagnose clinically. Diagnosis of TB pleuritis is facilitated by a chest X-ray, and this imaging study may not be available at many RNTCP diagnostic facilities, especially in rural areas [40]. Other forms of TB are considerably more difficult to diagnose without invasive tests.

We therefore assumed that extrapulmonary TB is more challenging to diagnose than smear-positive pulmonary TB, which by definition is easily diagnosable by sputum microscopy. We also conservatively assumed that extrapulmonary TB is easier to diagnose than smear-negative pulmonary TB, as some forms of extrapulmonary TB are more clinically evident (e.g., lymphadenitis) or serious enough to warrant hospitalization (e.g., meningitis). This assumption biases our estimates towards better outcomes for the RNTCP. We therefore estimated the proportion of extrapulmonary TB patients who remain undiagnosed despite presenting to a TB diagnostic facility by taking the average of the proportion of undiagnosed smear-positive TB patients and the proportion of undiagnosed smear-negative TB patients (S5 Text). This estimate for the proportion of undiagnosed extrapulmonary TB patients allowed us to estimate Step 2 for extrapulmonary TB by back-calculation from Step 3 (S5 Text).

To estimate Step 2 for MDR TB patients, we used the WHO estimate of the number of MDR TB patients among notified pulmonary TB patients in 2013 [1]. This estimate is derived from culture-based studies conducted at RNTCP sites in India that estimate the prevalence of MDR TB patients among both new and retreatment patients. In other words, this figure probably provides a relatively accurate estimate of the number of MDR TB patients who undergo diagnostic evaluation at RNTCP microscopy centers. This estimate is not the same as the number of MDR TB patients who are actually diagnosed in the public sector. Because most RNTCP diagnostic facilities still primarily use smear microscopy, many of these patients are not diagnosed with MDR TB and are instead misclassified as new or retreatment TB patients.

We also cross-checked this WHO MDR TB estimate using data from the recent study assessing the pilot implementation of Xpert MTB/RIF at 18 geographically diverse sites in India [24]. This study found that the proportion of TB suspects diagnosed with rifampin-resistant TB, which is presumed to be a marker of MDR TB, increased by 5.82 times between the baseline phase (when Xpert was not being used, but sputum smears were used with drug-susceptibility testing only on high-risk patients) and the intervention phase (when Xpert was used upfront for diagnosis and drug-susceptibility testing of all patients with suspected TB). In that study, 31 out of 10,907 diagnosed TB patients were found to have drug resistance at baseline compared to 1,190 out of 71,754 diagnosed TB patients after implementation of Xpert for upfront screening. Correcting this ratio for the fact that Xpert is only 67% sensitive for diagnosing smear-negative TB [30], we estimate a 6.89 times rate of underdiagnosis of true MDR TB patients evaluated at the vast majority of RNTCP diagnostic facilities relying on smear microscopy alone.

Since there were 9,466 (95%CI: 9,321–9,629) total MDR TB patients diagnosed nationwide in the RNTCP in 2012 (the year the Xpert study was conducted), we multiply this value by 6.89 to estimate that 65,221 (95%CI: 64,221–66,343) true MDR TB patients reached RNTCP diagnostic centers and underwent diagnostic evaluation for TB. Notably, this estimate is very similar to the WHO’s estimate of 64,000 MDR TB patients among all notified pulmonary TB patients in India in 2012 and 61,000 MDR TB patients in 2013. We therefore used the 2013 WHO estimate for Step 2 of the MDR TB cascade.

#### Diagnosis and registration in treatment

Step 3 and Gap 3: To estimate Step 3 (the number of patients diagnosed with TB) for new smear-positive and retreatment smear-positive TB patients, we extracted the total number smear-positive patients diagnosed in 2013 from the 2014 *TB India* report [16]. This value does not separate new smear-positive and retreatment smear-positive patients. We therefore assumed that the proportion of new smear-positive and retreatment smear-positive patients among all smear-positive patients diagnosed in Step 3 is the same as the proportion of new smear-positive and retreatment smear-positive patients among all smear-positive patients registered in treatment in Step 4. Based on this assumption, we estimated that 77.6% of all diagnosed smear-positive patients are “new” and 22.4% of all diagnosed smear-positive patients are “retreatment,” which allows us to estimate the number of new smear-positive and retreatment smear-positive patients in Step 3 (S5 Text).

We then estimated Gap 3 (pretreatment loss to follow-up) for new smear-positive and retreatment smear-positive patients by calculating the difference between estimates for Step 3 and Step 4 [16,20]. Estimating pretreatment loss to follow-up in this manner may have shortcomings. It is possible that some patients might submit sputum specimens at multiple microscopy centers, which would lead to overestimation of Gap 3. Alternatively, some patients may migrate or transfer to other centers and get re-registered in care, which could underestimate Gap 3. For this reason, we also crosscheck this estimate by independently estimating the rate of pretreatment loss to follow-up for smear-positive patients through a systematic review and meta-analysis of studies (Table H in S3 Text and results section below).

We could find no studies of pretreatment loss to follow-up among new smear-negative, extrapulmonary, or retreatment smear-negative patients to help inform our estimate of Step 3 and Gap 3 for these forms of TB; however, we estimated Step 3 and Gap 3 for these forms of TB based on our findings on smear-positive pretreatment loss to follow-up. As with smear-positive patients, most patients with these other forms of TB are diagnosed at government TB microscopy centers. After diagnosis, they are referred to directly observed therapy (DOT) centers close to their homes to start treatment. The studies of smear-positive pretreatment loss to follow-up identified in this systematic review suggest that most patients are lost to follow-up during the process of referral from government TB microscopy centers to DOT centers. Specifically, three of the studies in the review provide specific information on patient losses during the referral process, and we used the findings of these studies to estimate the proportion of all pretreatment loss to follow-up patients who are lost during the referral process [44–46].

Based on the overall smear-positive pretreatment loss to follow-up rate, we then estimated the proportion of all smear-positive patients who experience pretreatment loss to follow-up during the referral process. Since most new smear-negative, extrapulmonary, and retreatment smear-negative TB patients also undergo this referral process, we conservatively estimated that the pretreatment loss to follow-up rate (Gap 3) for these patients is the same as the proportion of smear-positive patients experience pretreatment loss to follow-up during the referral process. While some new smear-negative, extrapulmonary, and retreatment smear-negative patients will also be lost to follow-up prior to referral, we assumed that most of these patient losses will be captured in Gap 2 for these forms of TB (loss to follow-up during the diagnostic workup). Using this estimate for Gap 3, we were able to back-calculate Step 3 from Step 4 for new smear-negative, extrapulmonary, and retreatment smear-negative patients.

To estimate Step 3 and Gap 3 for MDR TB patients, we identified two studies that describe the pretreatment loss to follow-up rate (Gap 3) for patients diagnosed with MDR TB through our systematic review (Table H in S3 Text) [47,48]. We used the pooled prevalence of these findings to estimate Step 3 by back-calculation from Step 4.

Step 4: For all forms of TB, we estimated the number of patients registered in treatment in the RNTCP (Step 4) using values reported in the *TB India* reports [20,21].

#### Retention during the treatment course

Gap 4: Gaps 4a (loss to follow-up or death during the intensive phase of therapy) and 4b (loss to follow-up, treatment failure, or death during the continuation phase of therapy) could not be estimated separately since the *TB India* reports do not provide information on the phases of TB therapy during which these poor treatment outcomes occur. We therefore report Gaps 4a and 4b together as a combined Gap 4. We estimated the proportion of patients who died, failed treatment, or were lost to follow-up during treatment by calculating the difference between Step 4 and Step 5 using data from the *TB India* reports [16,20].

Step 5: We estimated Step 5 (the number of patients who achieved cure or treatment completion) using data for each form of TB from the *TB India* reports [16,20]. This estimation includes all forms of TB except for retreatment smear-negative patients. For retreatment smear-negative patients, we estimated Step 5 based on findings from the only Indian study reporting treatment outcomes for these patients, which describes a retreatment smear-negative treatment completion rate of 83.2% (95%CI: 81.0%–85.2%) [22].

#### Recurrence-free survival

Step 6 and Gap 5: The final outcome of the cascade—the number of patients who are recurrence-free 12 mo after completing TB treatment (Step 6)—was calculated by extrapolating from the estimates for Step 5 (the number of patients who achieve treatment completion) using estimates for Gap 5 (the proportion of patients who experience post-treatment recurrence or death within 12–24 mo) for each form of TB.

We estimated these proportions for Gap 5 based on a systematic review of local studies conducted in India’s public sector (Table K in S4 Text, and Results section, below). We estimated Gap 5 based on the pooled prevalence of findings from two studies of new smear-positive TB patients [14,25], four studies of new smear-negative and extrapulmonary TB patients [49–52], and one study of retreatment smear-positive patients [25]. We assumed that the proportion of patients who experience TB recurrence or death for retreatment smear-negative patients is the same as that for new smear-negative patients, given that these forms of TB have very similar treatment outcomes [22]. Since MDR TB patients have poor treatment outcomes, we made a conservative assumption that their rate of post-treatment TB recurrence and death is similar to that of retreatment smear-positive patients, who have the highest recurrence risk of any form of TB described in our systematic review.

## Results

### Systematic Reviews Informing Steps and Gaps in the TB Cascade

#### Chest symptomatics who do not get evaluated at TB diagnostic facilities (Gap 1b)

In the systematic review of care-seeking by people in the community with cough >2 wk (i.e., chest symptomatics), out of 1,679 abstracts found by searching the literature from between January 1, 2000, and October 1, 2015, we identified 219 eligible for full-text review, out of which 8 contain relevant data for extraction (Fig A in S2 Text). All eight studies evaluate the proportion of chest symptomatics who had not seen any provider by the time of the survey [32,53–59]; seven report whether patients sought care from public sector or private sector providers [32,53–56,58,59]; and three report the proportion who had not been screened for TB with a sputum test (Table C in S2 Text) [32,54,58].

Seven studies were conducted in 5 of India’s 36 states [32,53–56,58,59]. In addition, one study collected data from households in 30 districts located in numerous states [57]. Some of India’s poorest states with a high population, such as Uttar Pradesh and Madhya Pradesh, were included. Three studies were conducted in urban areas, three in rural areas, and two in both rural and urban areas (Table C in S2 Text). All of the studies are high quality with regard to sampling strategy and sample size; however, six of the eight studies did not report the proportion of people screened during their population-based data collection (low quality for this criterion), and six studies did not report the proportion of chest symptomatics identified who completed an interview (low quality) (Table C in S2 Text).

**Fig 2:**
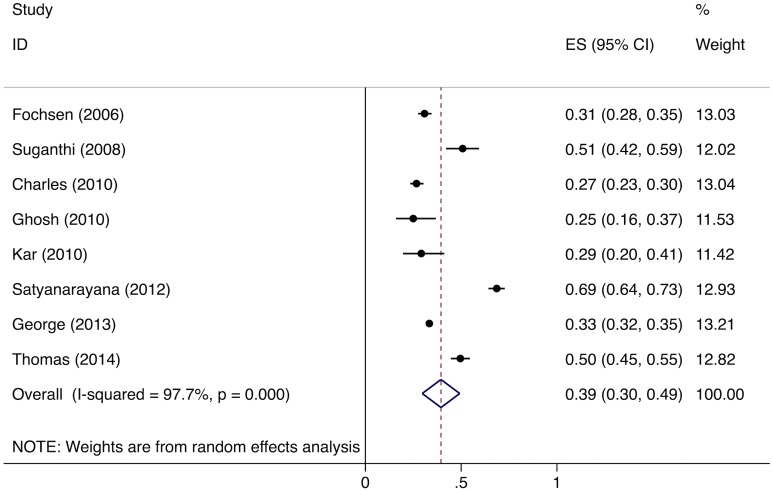
Forest plot of studies estimating the proportion of individuals in the community with cough >2 wk who report not having visited any medical provider after the onset of cough (Gap 1b). ES, effect size; CI, confidence interval.

**Fig 3:**
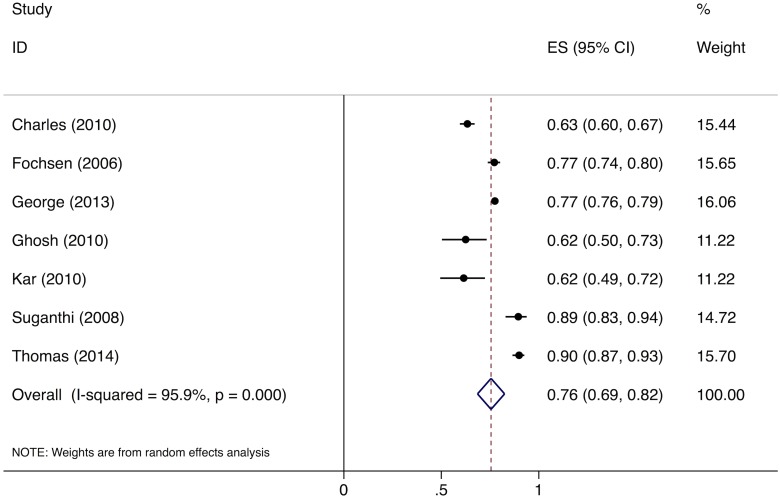
Forest plot of studies estimating the proportion of individuals in the community with cough >2 wk who report not having visited a public sector provider after the onset of cough (Gap 1b). ES, effect size; CI, confidence interval.

**Fig 4:**
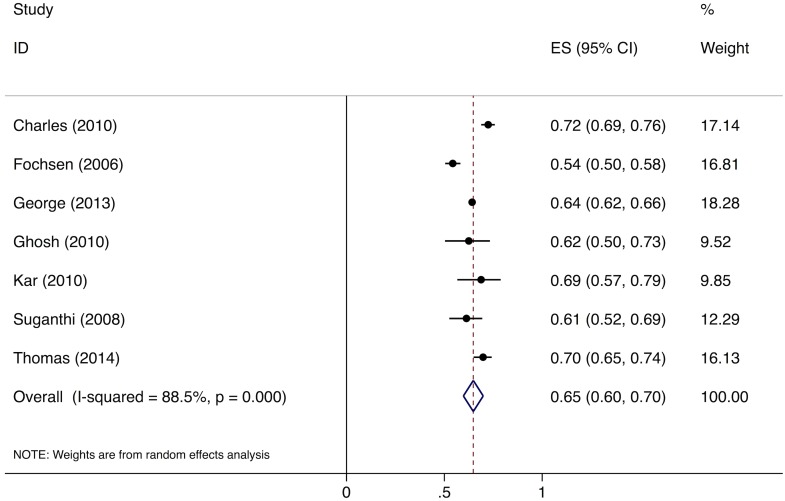
Forest plot of studies estimating the proportion of individuals in the community with cough >2 wk who report not having visited a private sector provider after the onset of cough (Gap 1b). ES, effect size; CI, confidence interval.

Meta-analysis of the eight studies estimating the proportion of individuals in the community with cough >2 wk who report not having visited any medical provider after the onset of cough show that, overall, 39% (95%CI: 30%–49%) of chest symptomatics had not seen any medical provider (Fig 2). Meta-analysis of the seven studies estimating the proportion of individuals with cough >2 wk who report not having visited a public sector provider shows that, overall, 76% (95%CI: 69%–82%) of chest symptomatics had not seen a public sector provider (Fig 3). Meta-analysis of the proportion of individuals with cough >2 wk who report not having visited a private sector medical provider shows that, overall, 65% (95%CI: 60%–70%) of chest symptomatics had not seen a private sector provider (Fig 4). Three studies reported the proportion of patients who had not received screening with sputum microscopy; these studies found that 91%–97% of chest symptomatics had not been screened with sputum microscopy at the time of the survey, even if they had been evaluated by a medical provider (Table C in S2 Text).

#### TB patients evaluated at diagnostic facilities who are not successfully diagnosed (Gap 2)

In the systematic review of studies of TB patients evaluated at diagnostic facilities who fail to get diagnosed with TB, out of 1,704 abstracts found by searching the literature from between January 1, 2000, and February 26, 2015, we identified 26 eligible for full-text review (Fig C in S3 Text). Of these, six studies contain relevant data on the proportion of chest symptomatics who failed to provide a second sputum sample [23,38,60–63]. Three studies contain data on the proportion of patients undergoing evaluation for smear-negative TB who fail to get a chest X-ray, which is the final step in the diagnostic workup [38–40].

The six studies describing the proportion of patients who failed to provide a second sputum sample were conducted in 7 out of India’s 36 states, with two studies focused solely on urban populations, two studies focused solely on rural populations, and two studies focused on both (Table F in S3 Text). The three studies describing the proportion of patients who completed the diagnostic workup for smear-negative TB were conducted in three different states, with one study conducted solely in a rural location and two studies conducted in both urban and rural locations (Table G in S3 Text).

For the studies evaluating the proportion of patients who failed to submit two sputum specimens, one study was medium quality because it was conducted at only one microscopy center at an urban tertiary care hospital; the studies were high quality on other indicators (Table F in S3 Text). One of the studies evaluating loss to follow-up during the smear-negative diagnostic workup was excluded due to a small sample size of <150 patients (Table G in S3 Text) [38].

**Fig 5:**
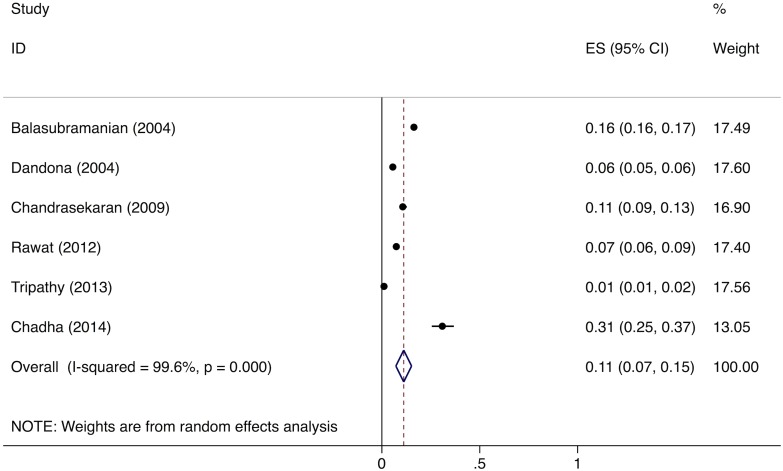
Forest plot of studies estimating the proportion of patients who fail to provide a second sputum smear (i.e., “diagnostic default”), which allows estimation of the proportion of smear-positive TB patients who might be “missed” at TB diagnostic facilities (Gap 2). ES, effect size; CI, confidence interval.

The meta-analysis of the six studies estimating the proportion of patients who fail to provide a second sputum sample shows that, overall, 11% (95%CI: 7%–15%) of chest symptomatics fail to provide a second sputum sample (Fig 5). Since there were fewer than five studies evaluating the proportion of patients who fail to complete the workup for smear-negative TB, we did not conduct a formal meta-analysis; however, these studies suggest that a large proportion (61%–80%) of TB suspects fail to complete the multistep diagnostic workup for smear-negative TB (Table G in S3 Text) [38–40].

#### Pretreatment loss to follow-up of smear-positive patients (Gap 3)

In the systematic review of studies of TB patients who get appropriately diagnosed but fail to get registered in treatment (i.e., pretreatment loss to follow-up), out of 1,704 abstracts found by searching the literature from between January 1, 2000, and February 26, 2015, we identified 26 studies eligible for full-text review (Fig C in S3 Text). Of these, 14 contain relevant data on pretreatment loss to follow-up of smear-positive TB patients [44–46,60,62–71], and two studies contain data on pretreatment loss to follow-up of MDR TB patients [47,48].

The studies of pretreatment loss to follow-up of smear-positive patients were conducted in 10 different Indian states. Four of these studies were conducted solely in rural areas; four were conducted solely in urban areas; and six were conducted in both (Table H in S3 Text). The studies of pretreatment loss to follow-up of MDR TB patients were conducted in both urban and rural settings in two different states (Table H in S3 Text).

Of all 16 pretreatment loss to follow-up studies, four are medium quality for sample selection, because they collected data from single microscopy centers, potentially limiting their representativeness at the local level (Table H in S3 Text). In addition, some studies were low quality for study methodology (five studies) and time frame of evaluation (11 studies) (Table H in S3 Text).

**Fig 6:**
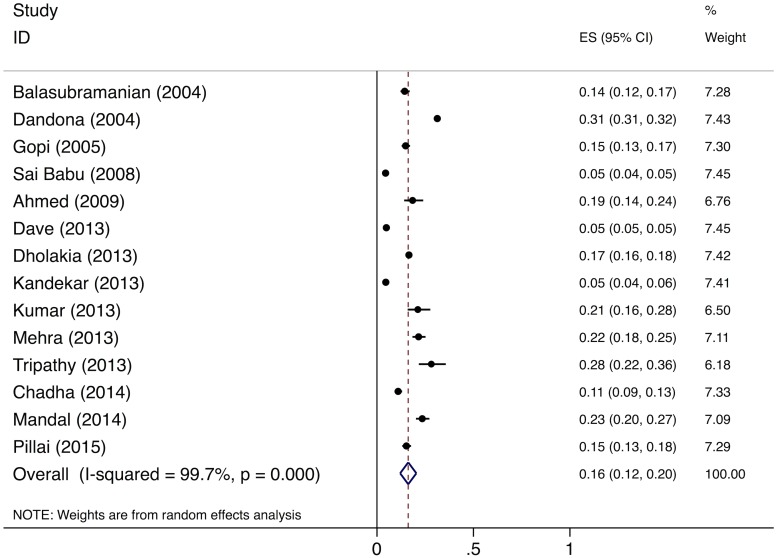
Forest plot of studies estimating pretreatment loss to follow-up (i.e., “initial default”) of smear-positive TB patients in India (Gap 3). ES, effect size; CI, confidence interval.

The meta-analysis of the 14 studies of smear-positive patients shows an overall pretreatment loss to follow-up rate of 16% (95%CI: 12%–20%) (Fig 6). Three of these studies provide specific information on the proportion of pretreatment loss to follow-up patients who are lost during the referral process. These studies report that 98/120 (81.6%) [44], 62/63 (98.4%) [45], and 75/145 (51.7%) [46] of pretreatment loss to follow-up among smear-positive patients occurred during referral, for a pooled prevalence of 235/328 (71.6%, 95%CI: 66.5%–76.3%). The pooled prevalence of the two studies of MDR TB patients shows a pretreatment loss to follow-up rate of 23.0% (95%CI: 21.8%–24.3%) (Table H in S3 Text).

#### Post-treatment TB recurrence and death (Gap 5)

In the systematic review of studies evaluating post-treatment TB recurrence and death, out of 2,547 short-listed abstracts found by searching the literature from between January 1, 2000, and October 9, 2015, we identified 30 studies eligible for full-text review (Fig D in S4 Text). Of these, 10 contain data on post-treatment TB recurrence or death in the RNTCP. Three of the studies evaluated predominantly HIV-negative smear-positive pulmonary TB patients who completed category I therapy; three evaluated HIV-infected smear-positive pulmonary TB patients who completed category I therapy; four studies evaluated a mixed population of smear-negative pulmonary, smear-positive pulmonary, and extrapulmonary TB patients who completed category I or III therapy; and one study evaluated retreatment smear-positive TB patients who completed category II therapy (Table K in S4 Text).

Studies were conducted in five Indian states. Eight studies were conducted in urban areas and two in rural areas (Table K in S4 Text). All of the studies are high-quality with regard to sampling strategy; however, two of the studies did not report the proportion of the cohort that was lost to follow-up during the study period [72,73]. Two of the studies describing TB outcomes among HIV-infected individuals are very low in quality because they have a sample size of <100 patients (Table K in S4 Text) [51,74].

We did not conduct a formal meta-analysis of these study findings, as none of these groups had five or more eligible studies; however, we estimated the pooled prevalence of relapse and death for these different types of TB from the limited available data. We excluded studies that did not have complete data for *both* death and TB recurrence and studies that did not have at least 12 mo and no more than 24 mo of follow-up.

For new smear-positive patients, we found two studies and estimate a pooled prevalence of 16.2% (95%CI: 14.25%–18.5%) for the combined outcome of TB recurrence and death [14,25]. For new smear-negative, extrapulmonary, and retreatment smear-negative patients, we found four studies and estimate a pooled prevalence of 8.8% (95%CI: 6.9%–11.1%) for the combined outcome of TB recurrence and death [49–52]. For retreatment smear-positive patients, we estimate a 27.3% (95%CI: 19.1%–37.4%) combined outcome of post-treatment TB recurrence and death from a single study [25]. Notably, HIV-infected TB patients of all types have more variable and often considerably higher rates of post-treatment TB recurrence and death (ranging from 9%–61%) compared to the general TB population [51,52,74,75]. This suggests that HIV co-infected patients may constitute a higher proportion of retreatment patients and poor outcomes in the RNTCP than would be suggested by their 6% prevalence in the general TB population.

### The TB Cascade of Care for 2013

**Fig 7:**
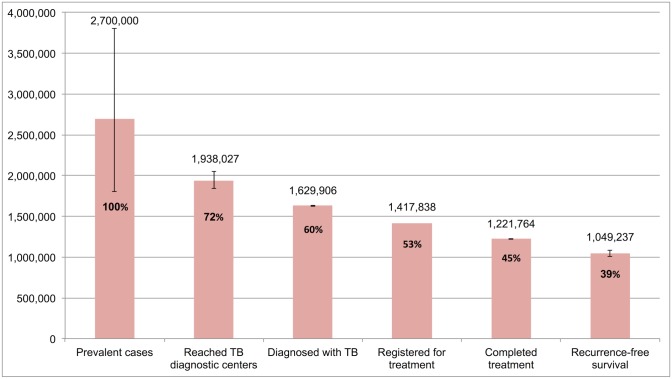
The cascade of care for all forms of tuberculosis in India’s Revised National Tuberculosis Control Programme (RNTCP) in India, 2013. Error bars depict 95% confidence intervals.

Table 4 and Fig 7 present the tuberculosis cascade of care in the government TB program in India for 2013. Of 2,700,000 (95%CI: 1,800,000–3,800,000) prevalent active TB patients in India, we estimate that 1,049,237 (95%CI: 1,008,775–1,083,243), or 38.9%, were successfully treated in the RNTCP and achieved at least 1 y of TB recurrence-free survival. We divide our discussion of estimates for each step and gap into the following sections: accessing care, diagnosis and registration in treatment, retention during the treatment course, and recurrence-free survival.

**Table 4:** Estimates for each step and gap in the cascade of care for different forms of tuberculosis in the Revised National Tuberculosis Control Programme (RNTCP), India, 2013.

	New smear-positive	New smear-negative	Extrapulmonary	Retreatment smear-positive	Retreatment smear-negative	Multidrug-resistant	Overall cascade
Step 1 (Prevalent TB patients)	--	--	--	--	--	--	2,700,000 (1,800,000–3,800,000)
Gaps 1a/1b (Patients who do not reach TB diagnostic facilities)	--	--	--	--	--	--	761,973 (-250,001–1,959,252)
Step 2 (Patients who are evaluated at TB diagnostic facilities)	730,108 (726,428–733,826)	514,161 (456,873–582,402)	310,284 (291,920–329,142)^b^	210,307 (209,247–211,378)	148,104 (131,602–167,760)	61,000 (47,000–76,000)	1,938,027 (1,840,748–2,050,001)
Gap 2 (Patients evaluated at TB diagnostic facilities who are not diagnosed)	9,491 (5,811–13,209)	193,179 (133,725–263,911)	60,505 (40,455–81,302)^b^	2,734 (1,674–3,805)	42,211 (24,995–62,689)	35,938 (21,507–51,322)	308,121 (205,845–425,731)
Step 3 (Patients successfully diagnosed with TB)	720,617^a^	320,982 (318,491–323,148)	249,779 (247,840–251,465)	207,573^a^	105,893 (105,071–106,607)	25,062 (24,678–25,493)	1,629,906 (1,624,270–1,634,903)
Gap 3 (Patients diagnosed with TB who are not registered in treatment)	105,008^a^	33,703 (31,212–35,869)	26,227 (24,288–27,913)	30,247^a^	11,119 (10,297–11,833)	5,764 (5,380–6,195)	212,068 (206,432–217,065)
Step 4 (Patients registered in treatment)	615,609^a^	287,279^a^	223,552^a^	177,326^a^	94,774^a^	19,298^a^	1,417,838^a^
Gap 4 (Patients who fail therapy, are lost to follow-up, or die during treatment)	73,873^a^	28,728^a^	15,649^a^	51,425^a^	15,922 (14,027–18,007)	10,477^a^	196,074 (194,179–198,159)
Step 5 (Patients who achieve cure or treatment completion)	541,736^a^	258,551^a^	207,903^a^	125,901^a^	78,852 (76,767–80,747)	8,821^a^	1,221,764 (1,219,679–1,223,659)
Gap 5 (Patients who experience TB recurrence or death)	87,761 (76,927–100,221)	22,752 (17,840–28,699)	18,295 (14,345–23,077)	34,371 (24,047–47,087)	6,939 (1,592–12,501)	2,408 (1,685–3,299)	172,527 (136,436–214,884)
Step 6 (Patients who achieve 1-y TB recurrence-free survival)	453,975 (441,515–464,809)	235,799 (229,852–240,711)	189,608 (184,826–193,558)	91,530 (78,814–101,854)	71,913 (68,246–75,175)	6,413 (5,522–7,136)	1,049,237 (1,008,775–1,083,243)

^a^No confidence intervals provided because the value is taken or extrapolated directly from absolute patient numbers reported in the *TB India* reports

^b^High degree of uncertainty; no studies in the published literature to facilitate this estimate

#### Accessing care

Of 2,700,000 prevalent active patients with TB (Step 1), we estimate that 1,938,027 (95%CI: 1,840,748–2,050,001), or 71.8%, of TB patients reached and were evaluated at government TB diagnostic facilities (Step 2), based on back-calculation for each form of TB from estimates for Gap 2 and Step 3 (S5 Text). This suggests that about 761,973 (95%CI: -250,001–1,959,252), or 28.2%, of prevalent TB patients did not have access to government TB facilities, never sought TB care, or directly sought TB care in the private sector (Gap 1a/1b).

No data were available to provide further insights into estimates for Gap 1a. For Gap 1b, as described above, the meta-analysis of studies evaluating care-seeking found that 39% (95%CI: 30%–49%) of individuals with cough >2 wk had not seen any medical provider at the time of the survey; 76% (95%CI: 69%–82%) had not seen a public sector provider; and 91%–97% of had not received evaluation for TB with sputum testing (Figs 2–4).

#### Diagnosis and registration in treatment

We estimated Gap 2—the proportion of TB patients who remain undiagnosed despite being evaluated at government TB diagnostic facilities—separately for each form of TB. For new smear-positive and retreatment smear-positive patients, we estimate that 11% (95%CI: 7%–15%) of people with suspected TB who present to government microscopy centers are lost to follow-up before submitting a second sputum specimen, based on the meta-analysis of six studies described above (Fig 5). Using an estimate of the incremental yield of a second sputum specimen for diagnosing smear-positive TB [37], we then estimate that 1.3% (95%CI: 0.08%–1.8%) of all smear-positive patients, or 9,491 (95%CI: 5,811–13,209) and 2,734 (95%CI: 1,674–3,805) new smear-positive and retreatment smear-positive patients respectively, remained undiagnosed despite presenting to government diagnostic facilities (S5 Text).

For new smear-negative patients, using the 1.42:1 (95%CI: 1.26:1 to 1.59:1) ratio of new smear-positive to new smear-negative TB patients seeking care in the public sector, we estimate that 514,161 (95%CI: 456,873–582,402) new smear-negative TB patients sought evaluation at government diagnostic facilities (Step 2) (S5 Text). Since 320,982 (95%CI: 318,491–323,148) new smear-negative TB patients were successfully diagnosed (Step 3), we estimate that 193,179 (95%CI: 133,725–263,911), or 37.6%, of patients with new smear-negative TB remained undiagnosed despite evaluation at government facilities (Gap 2). This estimate is consistent with the findings of our systematic review, which identified three studies showing that (61%–80%) of TB suspects fail to complete the multistep diagnostic workup for smear-negative TB, likely resulting in considerable under-diagnosis (Table G in S3 Text) [38–40].

For retreatment smear-negative patients, we also used a 1.42:1 (95%CI: 1.26:1 to 1.59:1) ratio of retreatment smear-positive patients to retreatment smear-negative patients to estimate Gap 2 (S5 Text). Of 148,104 (95%CI: 131,602–167,760) retreatment smear-negative patients evaluated at government diagnostic facilities (Step 2), we estimate that 105,893 (95%CI: 105,071–106,607), or 71.5%, were successfully diagnosed (Step 3) and 42,211 (95%CI: 24,995–62,689), or 28.5%, remained undiagnosed (Gap 2).

For extrapulmonary TB patients, we assume that 19.5% (95%CI: 15.1%–23.6%) of patients evaluated at TB diagnostic facilities remain undiagnosed (Gap 2). This suggests that about 310,284 (95%CI: 291,920–329,142) extrapulmonary TB patients were evaluated at TB diagnostic facilities (Step 2), of whom 249,779 (95%CI: 247,840–251,465) were successfully diagnosed (Step 3) and 60,505 (95%CI: 40,455–81,302) remained undiagnosed (Gap 2) (S5 Text).

For MDR TB patients, we use the WHO estimate of 61,000 (95%CI: 47,000–76,000) MDR TB patients among all notified TB patients in the RNTCP as the number of MDR TB patients who underwent evaluation at government TB diagnostic facilities (Step 2). We estimate that 25,062 (95%CI: 24,678–25,493), or 41.1%, of these patients were successfully diagnosed with MDR TB (Step 3), suggesting that 35,938 (95%CI: 21,507–51,322), or 58.9%, remained undiagnosed or got misdiagnosed with other forms of TB (Gap 2).

Aggregating these findings, we estimate that, of 1,938,027 (95%CI: 1,840,748–2,050,001) patients with all forms of TB who presented for evaluation to government TB diagnostic facilities (Step 2), 1,629,906 (95%CI: 1,624,270–1,634,903), or 84.1%, were appropriately diagnosed (Step 3) and 308,121 (95%CI: 205,845–425,731), or 15.9%, remained undiagnosed (Gap 2).

We also produced separate estimates for each form of TB for Gap 3—the proportion of patients diagnosed with TB who do not get registered in treatment in the government TB program (i.e., pretreatment lost to follow-up).

For Gap 3 among smear-positive TB patients, as noted above, the meta-analysis of findings from 14 studies suggests a pretreatment loss to follow-up rate of 16% (95%CI: 12%–20%) (Fig 6 and Table H in S3 Text). This finding is concordant with our Gap 3 estimate using data from the *TB India* reports (S5 Text) [16,20]. In that analysis, 928,190 new and retreatment smear-positive TB patients were diagnosed in 2013 (Step 3) and 792,935 (85.4%) new and retreatment smear-positive patients were registered in treatment (Step 4), suggesting there were 135,255 (14.6%) pretreatment loss to follow-up patients (Gap 3). Since this estimate is within the confidence interval of the meta-analysis estimate, we use the more conservative Gap 3 estimate from the *TB India* reports in the TB cascade.

For Gap 3 among new smear-negative TB patients, using the 10.5% (95%CI: 9.8%–11.1%) rate of pretreatment loss to follow-up, we estimate that 320,982 new smear-negative TB patients were diagnosed (Step 3). Since 287,279 new smear-negative patients were registered in treatment (Step 4), we estimate that 33,703 (95%CI: 31,212–35,869) were diagnosed but did not get registered in treatment (Gap 3) (S5 Text). Using the same rate of pretreatment loss to follow-up for extrapulmonary TB patients, we estimate that 249,779 patients were diagnosed (Step 3). Since 223,552 extrapulmonary TB patients were registered in treatment (Step 4), we estimate that 26,227 (95%CI: 24,288–27,913) were diagnosed but did not get registered in treatment (Gap 3) (S5 Text). Using the same rate of pretreatment loss to follow-up for retreatment smear-negative patients, we estimate that 105,893 patients were diagnosed (Step 3). Since 94,774 retreatment smear-negative patients were registered in treatment (Step 4), we estimate that 11,119 (95%CI: 10,297–11,833) were diagnosed but did not get registered in treatment (Gap 3) (S5 Text).

For MDR TB patients, using the Gap 3 estimate of 23.0% (95%CI: 21.8%–24.3%) from the meta-analysis, we estimate that 25,062 (95%CI: 24,678–25,493) MDR TB patients were diagnosed in 2013 (Step 3), of whom 19,298 (77.0%) got registered in treatment (Step 4).

Aggregating these estimates, we estimate that 1,629,906 (95%CI: 1,624,270–1,634,903) patients with all forms of TB were diagnosed in 2013 (Step 3), but only 1,417,838 (87.0%) patients ultimately got registered in treatment (Step 4), which suggests that 212,068 (95%CI: 206,432–217,065), or 13.0%, of all TB patients in India were diagnosed at government TB diagnostic facilities but lost to follow-up prior to starting treatment (Gap 3).

#### Retention during the treatment course

The numbers of new smear-positive, new smear-negative, extrapulmonary, and MDR TB patients who completed treatment were extracted from the *TB India* reports, and the estimates for Step 4, Gap 4, and Step 5 for these forms of TB are presented in Table 4 [20]. For retreatment smear-negative patients, one study describes a 83.2% (95%CI: 81.0%–85.2%) treatment completion rate in this patient population [22]. We therefore estimate that, of 94,774 retreatment smear-negative patients who started TB treatment (Step 4), 78,852 (95%CI: 76,767–80,747) achieved treatment completion (Step 5) and 15,922 (95%CI: 14,027–18,007) failed treatment, were lost to follow-up, or died during the treatment course (Gap 4).

Aggregating these estimates, we estimate that, of the 1,417,838 patients with all forms of TB who registered for treatment in the RNTCP in 2013 (Step 4), 1,221,764 (95%CI: 1,219,679–1,223,659), or 86.2%, successfully completed treatment (Step 5). This suggests that 196,074 (95%CI: 194,179–198,159), or 13.8%, of these patients failed treatment, were lost to follow-up, or died during the treatment course (Gap 4).

#### Recurrence-free survival

For new smear-positive patients, using the Gap 5 estimate of 16.2% (95%CI: 14.2%–18.5%) from the meta-analysis, we estimate that, of 541,736 patients who completed treatment (Step 5), 453,975 (95%CI: 441,515–464,809) achieved 1-y recurrence-free survival (Step 6) and 87,761 (95%CI: 76,927–100,221) experience TB recurrence or death within 12–24 mo of treatment completion (Gap 5).

For new smear-negative patients, using the Gap 5 estimate of 8.8% (95%CI: 6.9%–11.1%) from the meta-analysis, we estimate that, of 258,551 patients who completed treatment (Step 5), 235,799 (95%CI: 229,852–240,711) achieved 1-y recurrence-free survival (Step 6) and 22,752 (95%CI: 17,840–28,699) experienced post-treatment TB recurrence or death (Gap 5). Using the same 8.8% Gap 5 estimate for extrapulmonary TB patients, we estimate that, of 207,903 patients who completed treatment (Step 5), 189,608 (95%CI: 184,826–193,558) achieved 1-y recurrence free survival (Step 6) and 18,295 (95%CI: 14,345–23,077) experienced post-treatment TB recurrence or death (Gap 5). Using the same 8.8% Gap 5 estimate for retreatment smear-negative patients, we estimate that, of 78,852 patients who completed treatment (Step 5), 71,913 (95%CI: 68,246–75,175) achieved 1-y recurrence-free survival (Step 6) and 6,939 (95%CI: 1,592–12,501) experienced post-treatment TB recurrence or death (Gap 5).

For retreatment smear-positive patients, using the Gap 5 estimate of 27.3% (95%CI: 19.1%–37.4%) from the systematic review [25], we estimate that, of 125,901 patients who completed treatment (Step 5), 91,530 (95%CI: 78,814–101,854) achieved 1-y recurrence-free survival (Step 6) and 34,371 (95%CI: 24,047–47,087) experience post-treatment TB recurrence or death (Gap 5). Using the same 27.3% Gap 5 estimate for MDR TB patients, we estimate that, of 8,821 patients who completed treatment (Step 5), 6,413 (95%CI: 5,522–7,136) achieved 1-y recurrence-free survival (Step 6) and 2,408 (95%CI: 1,685–3,299) experienced post-treatment TB recurrence or death (Gap 5).

Aggregating these estimates, we estimate that of the 1,221,764 patients who successfully completed treatment in the RNTCP in 2013 (Step 5), 1,049,237 (95%CI: 1,008,775–1,083,243), or 85.9%, achieved the optimal outcome of 1-y TB recurrence-free survival (Step 6), and 172,527 (95%CI: 136,436–214,884), or 14.1%, experience TB recurrence or death within 12–24 mo of completing TB therapy (Gap 5).

### The TB Cascade of Care in Specific Subpopulations

We present the TB cascade of care for 2013 for specific subpopulations of TB patients. These cascades exclude Step 1 (the number of prevalent patients), since disaggregated prevalence estimates are not available for most forms of TB. Because they start from Step 2 (the number of patients evaluated at diagnostic facilities), these subpopulation cascades reflect the efficiency of RNTCP services in detecting, linking to care, treating, and retaining TB patients.

**Fig 8:**
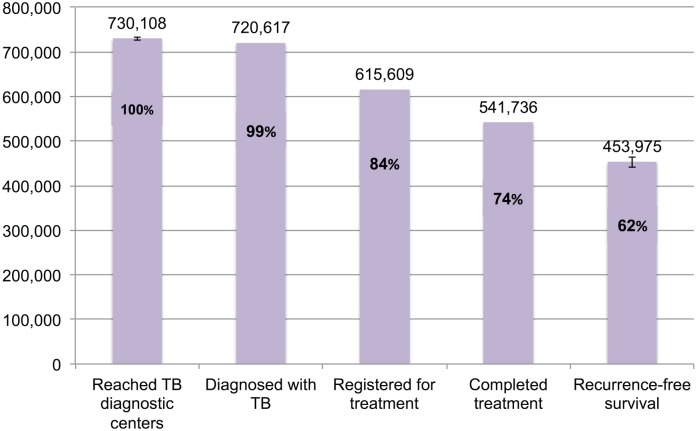
The tuberculosis cascade of care for new smear-positive tuberculosis patients detected and treated by the Revised National Tuberculosis Control Programme (RNTCP) in India, 2013. Error bars depict 95% confidence intervals.

Fig 8 presents the cascade for new smear-positive TB patients. Of the estimated 730,108 (95%CI: 726,428–733,826) patients who were evaluated at government diagnostic facilities, we estimate that 453,975 (95%CI: 441,515–464,809), or 62.2%, achieved 1-y recurrence-free survival. Moderate attrition of patients occurred at Gap 3 (diagnosed but not started on treatment), Gap 4 (loss to follow-up, failure, or death while on treatment), and Gap 5 (post-treatment TB recurrence or death).

**Fig 9:**
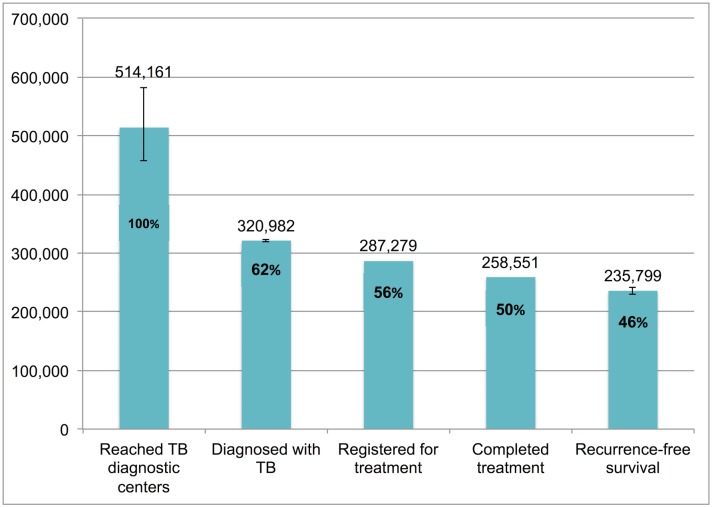
The tuberculosis cascade of care for new smear-negative tuberculosis patients detected and treated by the Revised National Tuberculosis Control Programme (RNTCP) in India, 2013. Error bars depict 95% confidence intervals.

Fig 9 presents the cascade for new smear-negative TB patients. Of the 514,161 (95%CI: 456,873–582,402) patients who were evaluated at government diagnostic facilities, we estimate that 235,799 (95%CI: 229,852–240,711), or 45.9%, achieved 1-y recurrence-free survival. The most substantial attrition occurred at Gap 2 (evaluated at diagnostic facilities but not diagnosed with TB).

**Fig 10:**
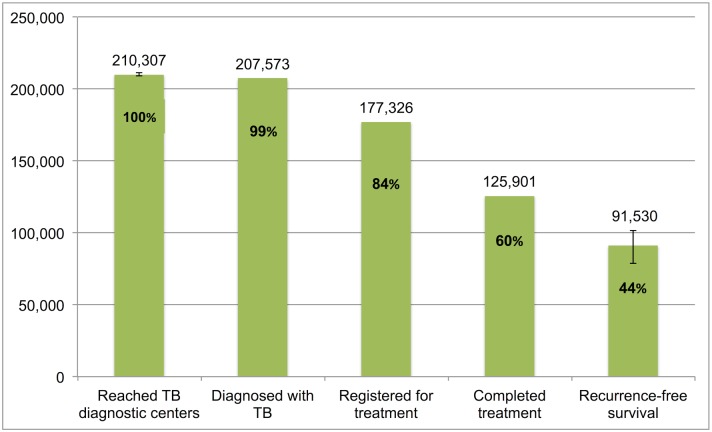
The tuberculosis cascade of care for retreatment smear-positive tuberculosis patients detected and treated by the Revised National Tuberculosis Control Programme (RNTCP) in India, 2013. Error bars depict 95% confidence intervals.

Fig 10 presents the cascade for retreatment smear-positive TB patients. Of the estimated 210,307 (95%CI: 209,247–211,378) patients who were evaluated at government diagnostic facilities, we estimate that 91,530 (95%CI: 78,814–101,854), or 43.5%, achieved 1-y recurrence-free survival. The most substantial attrition occurred at Gap 4 (loss to follow-up, failure, or death while on treatment) and Gap 5 (post-treatment TB recurrence or death).

**Fig 11:**
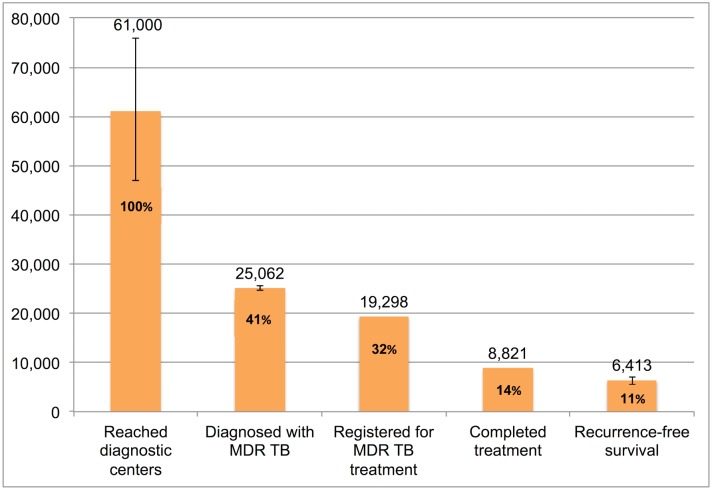
The tuberculosis cascade of care for multidrug-resistant tuberculosis (MDR TB) patients detected and treated by the Revised National Tuberculosis Control Programme (RNTCP) in India, 2013. Error bars depict 95% confidence intervals for each estimate.

Fig 11 presents the cascade for MDR TB patients. We estimate that, of the 61,000 (95%CI: 47,000–76,000) MDR TB patients who reached government TB diagnostic facilities, only 8,821 (14.4%) were appropriately diagnosed and completed MDR TB therapy and 6,413 (95%CI: 5,522–7,136), or 10.5%, ultimately achieved 1-y recurrence free survival. While there is considerable attrition at every step of the cascade, Gap 2 (evaluated at diagnostic facilities but not diagnosed with MDR TB) contributed most to the cascade’s poor performance.

## Discussion

We constructed the cascade of TB care in India for 2013 with two different aims—(1) to identify critical points of patient attrition that may guide TB control interventions and (2) to identify knowledge gaps for different steps of the cascade in order to guide future research to improve its accuracy.

### Critical Points of Attrition in the Cascade

For the overall cascade including all forms of TB, the most substantial point of attrition is Gap 1a/1b, which suggests that more than one-quarter of prevalent TB patients in India (or about 760,000 patients annually) are not evaluated at government TB diagnostic centers. In addition, our estimates for Gaps 2 and 3 together suggest that nearly one-fifth of prevalent TB patients (or about 520,000 patients annually) interface with public sector TB services but are either not successfully diagnosed or are diagnosed with TB but lost to follow-up before starting treatment. Together, Gaps 1, 2, and 3 indicate that nearly 1.3 million TB patients in India do not receive therapy in the public sector—a finding that roughly corroborates the WHO’s estimate of “missing” TB patients in India [1,12,13].

National-level surveys suggest that a considerable proportion of these “missing” patients in Gaps, 1, 2, and 3 are being treated by private sector providers [12,13]. The private sector in India is very heterogeneous, and studies suggest that, in some locations, the majority of care is provided by practitioners of Indian medical traditions (e.g., Ayurveda, Yoga, Unani, Siddha, and Homeopathy [AYUSH]) or informal providers, who often have no formal medical degrees [76]. The few available data suggest lower quality of care [3,4] and poorer treatment completion rates (about 50%) [77] in private facilities when compared to the public sector (~85%), even in the context of public–private partnerships aimed at improving private sector care.

To decrease Gap 1a/1b, more research is needed to understand the TB diagnostic and referral practices of private providers, especially AYUSH and informal practitioners who provide most community-level care [76]. Data from Mumbai suggest that, when they do suspect TB, most private providers prefer referring patients to public sector TB services or to private chest physicians with expertise in managing TB [78]. However, a study in Delhi showed that only one-fifth of unannounced standardized patients presenting to private providers with a classic TB history were correctly managed with referral to public or private TB services or ordering of an appropriate TB test (e.g., sputum microscopy) [4].

These findings highlight a need to bridge private providers’ “know–do” gap—the gap between their intentions and actual practice—to increase referrals to the public sector and case notifications. Enhanced engagement of the RNTCP with private providers, through robust continuing medical education initiatives, on-site visits from RNTCP clinicians to review TB patient management strategies, collaborative efforts at patient co-management with provision of free medications from the public sector, and incentives to promote referral of TB patients, may help to reduce this gap. At the same time, the cascade model emphasizes that improving private sector case notification alone is inadequate; it is equally important to routinely collect data on treatment outcomes and 1-y TB recurrence for patients treated in the private sector to monitor this sector’s quality of care.

The proportion of patients who never seek any TB care and remain untreated in Gap 1a/1b remains uncertain, though our meta-analysis shows that about 39% of chest symptomatics had not sought medical care at the time they were surveyed, which at minimum suggests delayed care-seeking behavior. Public education may help to improve care-seeking behavior, which can be monitored using serial population-based surveys. In addition, enhancing the availability and accessibility of RNTCP facilities in hard-to-reach Adivasi (tribal) areas, where case detection rates are very low, may help to reduce the proportion of India’s population without access to TB care [31]. Finally, various strategies of targeted active case finding—especially higher yield strategies focused on repeated screening of household contacts of known TB patients [79], people living with HIV [80,81], and individuals visiting health care facilities [82]—may also help to substantially reduce Gap 1a/1b by identifying patients who may otherwise not seek TB care or do so with substantial delay [83].

Gaps 2 and 3 are notable because they suggest that about one-fifth of all TB patients in India are “lost” despite engaging with government TB services. These patients go undetected during the diagnostic workup at government facilities (possibly due to heavy reliance on insensitive diagnostics such as smear microscopy) or fail to start TB treatment despite being successfully diagnosed. Since this population of half a million patients is willing to engage with public sector services in some capacity, they present a major opportunity for strengthening TB control. Improving care at public sector facilities may help to retain these patients—by increasing case detection (e.g., through use of more sensitive and rapid molecular tests), accelerating the time to treatment initiation, improving linkage of diagnosed patients to care, and making TB services more patient-friendly. In addition, since most diagnosed patients in Gap 2 are lost during the process of referral from TB microscopy centers to local DOT centers [44–46], strengthening patient tracking and improving the accountability of the health system for these patients may help address this gap.

While the RNTCP emphasizes the importance of treatment completion rates, our model suggests that the gap immediately after treatment—post-treatment TB recurrence and death (Gap 5)—may be an equally important contributor to the poor performance of the cascade. Routine follow-up of all patients for 1 y after treatment may have substantial benefits for TB control by promoting early detection of patients with recurrent TB, who are more likely to have drug resistant disease. The yield of this approach for detecting new patients may be comparable or superior to other strategies of case finding, such as household contact tracing [79,84].

The cascades for different subpopulations highlight different patterns of attrition for each form of TB. For new smear-positive patients, multiple steps—pretreatment loss to follow-up, loss to follow-up on treatment, and post-treatment TB recurrence or death—are equally important points of attrition.

In contrast, for new smear-negative patients, failure to detect patients who present to diagnostic facilities (Gap 2) is the major point of attrition. In fact, we probably underestimate the size of Gap 2 for these patients, because studies suggest that non-adherence of providers to the diagnostic algorithm for smear-negative TB may be resulting in considerable overdiagnosis [85]. As a result, patients with other pulmonary diseases might be incorrectly treated as having smear-negative TB, a problem that we were not able to correct for in this model. Concurrent use of alternative or more sensitive diagnostic tests (e.g., Xpert MTB/RIF or culture) may reduce this gap. In addition, an emerging revision to the RNTCP smear-negative diagnostic algorithm, in which chest X-ray is used upfront along with sputum smears, may reduce this gap [86].

For retreatment smear-positive patients, poor outcomes while taking TB therapy and high post-treatment recurrence and death rates are major points of attrition. Both of these points of attrition highlight a need for improved care of these patients after they register for treatment, potentially through better access to drug-susceptibility testing, enhanced counseling, and closer adherence monitoring. While HIV co-infected TB patients were not analyzed separately in the cascade, the results of our systematic review of TB recurrence suggests that these patients may also have substantially poorer treatment outcomes and higher rates of post-treatment TB recurrence and death [51,52,74,75]. For MDR TB patients, the cascade suggests that the vast majority of MDR TB patients who present to government diagnostic facilities are not diagnosed with drug resistance, highlighting an urgent need to move towards universal drug-susceptibility testing, as recommended by the End TB Strategy [6].

### Limitations and Improving the Accuracy of the Cascade

A major limitation of this study is the substantial uncertainty in the estimate of prevalent TB patients (Step 1), since India has not had a nationally representative TB prevalence survey conducted in decades. This figure also represents an estimate of point prevalence and is possibly an underestimate of the 1-y period prevalence of TB. Also, prior TB prevalence studies conducted in India have usually screened individuals in the community for TB symptoms before collecting sputum specimens [33,35,36]; this approach may miss a considerable proportion of patients without symptoms, thereby underestimating TB prevalence [87]. In addition, a recent study estimating the number of TB patients treated in India’s private sector suggests that the overall burden of TB disease in India may be underestimated [88]. While the uncertainty in the overall estimate of TB prevalence may impact interpretation of subsequent cascade steps, analysis of the cascade can also be performed starting from Step 2 (the number of TB patients evaluated at diagnostic facilities) and still provide substantial insights into the efficiency and functioning of the RNTCP.

A second major limitation of this study is the lack of data on the number of TB patients who are detected, treated, and cured in the private sector. Similar to the WHO’s estimate of “missing” TB patients, our model assumes that these patients are essentially undiagnosed and untreated. In reality, a considerable proportion of these patients have likely sought diagnosis and treatment in the private sector [12,13,89]. As such, we overestimate the gap in case detection and underestimate subsequent steps of the cascade. It is critical that the RNTCP strengthen private sector notification and, perhaps more importantly, routinely assess treatment outcomes for private sector patients.

A third limitation is the fact that we do not adequately account for possible misclassification of patients who may have diagnoses other than TB or who may have other forms of TB. We did assume, for example, that most MDR TB patients in Step 2 are subsequently misclassified and treated as either “new” or “retreatment” patients. However, due to limited data, we did not account for potential misdiagnosis with TB of patients who actually have other pulmonary processes, including non-tuberculous mycobacterial infection [90]. Due to poor adherence to the diagnostic algorithm, misdiagnosis with TB may especially be a problem for patients who have negative sputum smears [85]. In addition, one study suggests that as many as 9%–13% of new smear-positive patients started on treatment have a history of previous TB that was missed by providers, which means that these patients should have been screened for MDR TB and treated as retreatment smear-positive patients [91].

A fourth general limitation of our study is the inability to exclude duplicate patient records, since we used aggregate data from the RNTCP to construct the cascade. A small percentage of patients may have submitted sputum specimens at multiple facilities or have been registered for treatment at multiple DOT centers, thereby leading to overestimation of certain steps and underestimation of gaps. Preliminary analysis using computerized RNTCP records in Gujarat by one of this manuscript’s co-authors (KR) suggests that the percentage of duplicate records for smear-positive patients is likely very small (about 0.02%) [92]. The ongoing rollout of E-NIKSHAY, the RNTCP’s computerized case notification system, and India’s ongoing Unique ID (Aadhar) card program may facilitate more accurate nationwide estimates of duplicate records in the future.

A fifth general limitation is that time delays between steps are not well captured. This problem is especially relevant to patient care-seeking and case detection, since time delays (and not just the gaps alone) contribute to TB transmission. A recent systematic review suggests a median of nearly 2 mo delay in TB patients in India getting diagnosed after development of symptoms [93]. In addition, understanding the timing of loss to follow-up during TB treatment may help to target interventions for retaining patients on therapy.

A sixth general limitation is that we do not disaggregate the different types of poor outcomes in the cascade—loss to follow-up, death, treatment failure, and misclassification of patients. Since these are collapsed into single gaps in our current model, this limits understanding of the transmission risks associated with specific gaps, as some poor outcomes (e.g., treatment failure or loss to follow-up) may be associated with higher transmission than others (e.g., death). Incorporating a detailed description of poor outcomes into future cascades may help guide the development of interventions to reduce these gaps. A seventh general limitation is that we do not present longitudinal trends in the cascade. As the RNTCP is currently undergoing multiple transitions, including scale-up of Xpert MTB/RIF and switching from thrice weekly to daily TB therapy for all patients, longitudinal evaluation of the cascade in the future may help to measure changes in the performance of the overall program.

Other study limitations are specific to each step of the cascade and are summarized in Table 5, with suggested strategies for improving the accuracy of each estimate. For example, Gap 1b—the proportion of TB patients who never seek care—is very difficult to estimate; however, this indicator may be assessed indirectly by using serial surveys of care-seeking behavior by chest symptomatics or by conducting nationally-representative verbal autopsy studies that assess whether patients who died of TB ever sought care. Alternatively, strengthening the medical certification process for documenting causes of death, with subsequent audits of TB deaths to see whether patients had sought care, may be preferable. Gap 2—the proportion of TB patients seen at government diagnostic facilities who are not diagnosed—could best be assessed for smear-negative and MDR TB patients through studies in which mycobacterial culture is used to identify “true” TB patients, so that diagnostic attrition rates can be more accurately estimated.

**Table 5:** Limitations of current estimates and recommendations for improving the accuracy of the TB cascade of care for India.

Forms of TB	Limitations and biases of current estimates	Optimal data needed to improve estimates
Step 1: Total prevalent TB patients		
All forms of TB	No recent national prevalence survey; cities are underrepresented in recent regional surveys; WHO point prevalence estimate underestimates 1-y period prevalence; asymptomatic/subclinical TB not accounted for	National or state level TB prevalence surveys that also evaluate for asymptomatic TB in the community; regional data should be supplemented with data from high-burden cities
Gap 1a: Patients with no access to government TB facilities		
All forms of TB	No national survey data identifying localities with inadequate access to RNTCP services	Collection of data on access to RNTCP services in national household surveys like the National Family Health Survey
Gap 1b: Patients with access to government TB facilities who do not go to these facilities		
All forms of TB	Indicator is inherently difficult to estimate; evaluation of surrogate indicators may be required, such as chest symptomatic care-seeking and the proportion of TB-related deaths who did not access care	Assessment of chest symptomatic care-seeking can be routinely incorporated into TB prevalence surveys; verbal autopsy studies can assess whether patients who died of TB accessed care prior to death
Gap 2: Patients presenting to government TB facilities who are not diagnosed		
NSN / RO	Magnitude of underdiagnosis of NSN and RO patients uncertain without TB culture-based studies	TB culture-based studies will help determine the proportion of true NSN and RO patients lost during the diagnostic workup
EPTB	No studies identified that evaluate attrition during the diagnostic workup for EPTB	Studies of attrition of EPTB suspects during the diagnostic workup are needed
MDR TB	Magnitude of underdiagnosis of MDR TB uncertain without TB culture-based studies	Culture-based studies will help determine the proportion of MDR TB patients who are never diagnosed or misclassified as retreatment patients
Step 3: Number of patients diagnosed with TB		
All forms of TB, especially NSN, EP TB, MDR TB	Unlike for NSP patients, RNTCP statistics do not report the proportion of NSN, EP TB, RO and MDR TB patients who are diagnosed separately from the number registered for treatment	All forms of TB, including NSN, EP TB, RO, and MDR TB patients should be notified to the RNTCP at the time of diagnosis, rather than at the time of treatment registration
Gap 3: Patients diagnosed but not registered in treatment		
NSN, EPTB, RO, MDR TB	No studies identified that describe pretreatment loss to follow-up of NSN, EP TB, RO, or MDR TB patients, in contrast to the multiple studies describing this gap for NSP and RSP patients	Local studies of pretreatment loss to follow-up of these other forms of TB are needed; studies can focus on loss to follow-up after diagnosed patients are referred to DOT centers, as this is when most patients are lost
Step 4: Number of patients registered for treatment		
NSP, RSP, NSN	One study suggests that 9%–13% of NSP patients have a history of prior TB treatment that was “missed” and therefore could possibly be misclassified RSP or MDR TB patients [91]; studies suggest poor adherence to the NSN algorithm may result in empiric treatment of patients who have medical conditions other than TB	More robust multisite studies are needed to estimate the proportion of NSP patients with a history of “missed” prior TB treatment; TB culture-based studies will help determine the proportion of true NSN patients and the proportion of smear-negative patients started on TB treatment who actually have medical conditions other than TB
Gaps 4a/4b: Patients lost to follow-up or deaths early versus late in treatment		
All forms of TB	RNTCP does not report whether patients on treatment are lost to follow-up during the intensive versus the continuation phase	Routine reporting of lost to follow-up or death during each phase of therapy may help in targeting interventions
Gap 5: Patients who experience TB recurrence or death within 1 y of treatment completion		
All forms of TB	Local studies only evaluate post-treatment TB recurrence and death for some forms of TB; the quality of these studies is poor	Multisite study of post-treatment recurrence or death is needed; RNTCP can routinely assess 1-y post-treatment outcomes for all patients

TB, tuberculosis; NSP, new smear-positive tuberculosis; RSP, retreatment smear-positive tuberculosis; NSN, new smear-negative tuberculosis; RO, retreatment smear-negative tuberculosis; EP TB, extrapulmonary tuberculosis; MDR TB, multidrug-resistant tuberculosis; RNTCP, Revised National Tuberculosis Control Programme.

To best estimate Gaps 4a and 4b, the RNTCP should routinely collect and report data on when patients are lost or die while taking TB treatment, as this will help to focus resources for patient retention earlier or later in the treatment course. For Gap 6—the proportion of patients who experience post-treatment TB recurrence or death—higher quality studies are needed that estimate this indicator for all forms of TB, especially since routine follow-up of patients post-treatment may be an efficient strategy for early detection of recurrent and potentially drug-resistant TB patients.

The most recent year for which we could construct a complete cascade that included all forms of TB was 2013. Since the treatment course for MDR TB is prolonged (often >18 mo), treatment outcomes are not reported until 2–3 y after these patients are registered for treatment. Since 2013, MDR TB case detection has improved in India, due to increased use of Xpert MTB/RIF and line probe assays at select RNTCP sites to diagnose drug resistance, especially in patients with HIV or a prior history of TB [24]. Indeed, recent *TB India* reports suggest a considerable increases in the number of MDR TB patients registered for treatment from 12,285 in 2013 to 20,087 in 2014 and 26,966 in 2015 [16,20,21]. Therefore, the cascade presented here may provide baseline data against which emerging national efforts to improve MDR TB case detection and treatment can be evaluated.

Finally, if our model of the TB cascade is applied to other settings, it may require considerable modification to adjust for local differences in TB epidemics. For example, in using recurrence-free survival as the end outcome of the cascade, we assume that most TB recurrence is secondary to relapse of the same strain of TB [15]. However, this may be a less reliable end outcome in settings with a high prevalence of HIV infection, since a higher proportion of TB recurrences may be due to exogenous reinfection with a new strain of TB rather than relapse, which means that recurrences are less likely to reflect the quality of prior TB care received by the patient [94,95]. In addition, our cascade model will have to be modified in settings where Xpert is the predominant diagnostic test, as our analysis assumes the smear microscopy is the most common method of diagnosis.

### Conclusions

We describe the TB cascade of care in India for 2013 and estimate that about 39% of prevalent TB patients are treated in the government system and achieve an optimal outcome of 1-y recurrence free survival. Cascades constructed for subpopulations of TB patients highlight substantial attrition and poorer outcomes among retreatment smear-positive and MDR TB patients, and there are differences in the critical points of attrition for each type of TB.

TB control efforts in India are currently in the midst of a dramatic transition. The rollout of E-NIKSHAY, the RNTCP’s electronic data management system, may help to improve private sector case notification. Implementation of novel public–private partnerships in major Indian cities may increase case detection, improve private sector quality of care, and capture data on private sector treatment outcomes. Increased use of new diagnostic tests such as Xpert may reduce the considerable gaps in diagnosis of both smear-negative and MDR TB. Newer patient-centered models of care using electronic observation of therapy (e.g., through cell phones or electronic pillboxes) have the potential to improve patient retention throughout the treatment course.

In this context of rapid change, greater commitment of resources for TB control at the international, national, and state levels will be crucial for improving the outcomes of the TB cascade. The cascade model could help to identify gaps in care in a manner that might inform the most cost-effective targeting of resources in the public sector TB program. As with the Joint United Nations Programme on HIV/AIDS (UNAIDS)’ “90-90-90” global strategy for HIV, which is based on the HIV cascade model, future multisite studies of the TB cascade in India could be used to longitudinally measure TB control outcomes in a manner that accelerates progress towards the goals of the End TB Strategy [6]. In addition, since substantial numbers of India’s TB patients seek care in the private sector [12,88], a cascade of care analysis is urgently needed to identify critical gaps in patient care-seeking, diagnosis, retention in treatment, and disease recurrence in the private sector.

## Supporting Information

S1 Text PRISMA checklist for the three systematic reviews included in this manuscript. (PDF)

S2 Text Methods for the systematic review and meta-analysis of care-seeking by people with suspected TB. (PDF)

S3 Text Methods for the systematic review and meta-analysis of failure to complete the diagnostic workup and pretreatment loss to follow-up. (PDF)

S4 Text Methods for the systematic review and meta-analysis of studies evaluating TB recurrence or death after treatment completion or cure. (PDF)

S5 Text Detailed calculations for steps and gaps of the TB cascade of care in India, 2013. (PDF)

## Abbreviations

AYUSH: Ayurveda, Yoga, Unani, Siddha, and Homeopathy
DOT: directly observed therapy
MDR: multidrug-resistant
TB: tuberculosis
RNTCP: Revised National TB Control Programme
