# Supplementary material for: The Tuberculosis Cascade of Care in India’s Public Sector: A Systematic Review and Meta-analysis
Source: PLoS Med. 2016 Oct 25;13(10):e1002149. doi: 10.1371/journal.pmed.1002149 (PMC5079571; doi:10.1371/journal.pmed.1002149)
Supplement: S1 Text — (PDF) [file pmed.1002149.s001.pdf]

S1 Text: PRISMA 2009 Checklist for the systematic reviews included in the manuscript "The Tuberculosis Cascade of Care in India's Public Sector: A Systematic Review and Meta-Analysis"

Section/topic	#	Checklist item	Care-seeking systematic review (S2 Text)<br><br>Reported on page #	Failure to complete the diagnostic workup and pretreatment loss to follow-up (S3 Text)<br><br>Reported on page #	Tuberculosis recurrence (S4 Text)<br><br>Reported on page #
**TITLE**					
Title	1	Identify the report as a systematic review, meta-analysis, or both.	Main manuscript title, S2 Text title	Main manuscript title, S3 Text title	Main manuscript title, S4 Text title
**ABSTRACT**					
Structured summary	2	Provide a structured summary including, as applicable: background; objectives; data sources; study eligibility criteria, participants, and interventions; study appraisal and synthesis methods; results; limitations; conclusions and implications of key findings; systematic review registration number.	Main manuscript abstract	Main manuscript abstract	Main manuscript abstract
**INTRODUCTION**					
Rationale	3	Describe the rationale for the review in the context of what is already known.	Main manuscript "Introduction" section paragraphs 1-5; S2 Text "Objectives" section	Main manuscript "Introduction" section paragraphs 1-5; S3 Text "Objectives" section	Main manuscript "Introduction" section paragraphs 1-5; S4 Text "Objectives" section
Objectives	4	Provide an explicit statement of questions being addressed with reference to participants, interventions, comparisons, outcomes, and study design (PICOS).	Main manuscript "Methods" section	Main manuscript "Methods" section	Main manuscript "Methods" section

			paragraphs 1-13; S2 Text "Objectives" section	paragraphs 1-13; S3 Text "Objectives" section	paragraphs 1-13; S4 Text "Objectives" section
**METHODS**					
Protocol and registration	5	Indicate if a review protocol exists, if and where it can be accessed (e.g., Web address), and, if available, provide registration information including registration number.	Full details on the methodology of the review available in the manuscript supplementary text files	Full details on the methodology of the review available in the manuscript supplementary text files	Full details on the methodology of the review available in the manuscript supplementary text files
Eligibility criteria	6	Specify study characteristics (e.g., PICOS, length of follow-up) and report characteristics (e.g., years considered, language, publication status) used as criteria for eligibility, giving rationale.	S2 Text (Table C)	S3 Text (Tables F, G, H)	S4 Text (Table K)
Information sources	7	Describe all information sources (e.g., databases with dates of coverage, contact with study authors to identify additional studies) in the search and date last searched.	Main manuscript pages "Data sources" subsection of the "Methods" section; S2 Text "Search strategy" section	Main manuscript pages "Data sources" subsection of the "Methods" section; S3 Text "Search strategy" section	Main manuscript pages "Data sources" subsection of the "Methods" section; S4 Text "Search strategy" section
Search	8	Present full electronic search strategy for at least one database, including any limits used, such that it could be repeated.	S2 Text (Table A)	S3 Text (Table D)	S4 Text (Table I)
Study selection	9	State the process for selecting studies (i.e., screening, eligibility, included in systematic review, and, if applicable, included in the meta-analysis).	S2 Text "Inclusion and Exclusion criteria" and "Study	S3 Text "Inclusion and Exclusion criteria" and "Study	S4 Text "Inclusion and Exclusion criteria" and "Study

			selection” sections	selection” sections	selection” sections
Data collection process	10	Describe method of data extraction from reports (e.g., piloted forms, independently, in duplicate) and any processes for obtaining and confirming data from investigators.	S2 Text “Data extraction and analysis” section	S3 Text “Data extraction and analysis” section	S4 Text “Data extraction and analysis” section
Data items	11	List and define all variables for which data were sought (e.g., PICOS, funding sources) and any assumptions and simplifications made.	S2 Text “Data extraction and analysis” section and Table C	S3 Text “Data extraction and analysis” section and Tables F-H	S4 Text “Data extraction and analysis” section and Tables K
Risk of bias in individual studies	12	Describe methods used for assessing risk of bias of individual studies (including specification of whether this was done at the study or outcome level), and how this information is to be used in any data synthesis.	S2 Text “Quality assessment” section and Table B	S3 Text “Quality assessment” section and Table E	S4 Text “Quality assessment” section and Table J
Summary measures	13	State the principal summary measures (e.g., risk ratio, difference in means).	S2 Text “Objectives” section	S3 Text “Objectives” section	S4 Text “Objectives” section
Synthesis of results	14	Describe the methods of handling data and combining results of studies, if done, including measures of consistency (e.g., I^2) for each meta-analysis.	S2 Text “Data extraction and analysis”	S3 Text “Data extraction and analysis”	S4 Text “Data extraction and analysis”

Section/topic	#	Checklist item	Care-seeking systematic review (S2 Text) Reported on page #	Failure to complete the diagnostic workup and pretreatment loss to follow-up (S3 Text) Reported on page #	Tuberculosis recurrence (S4 Text) Reported on page #

Risk of bias across studies	15	Specify any assessment of risk of bias that may affect the cumulative evidence (e.g., publication bias, selective reporting within studies).	Note that there are no great instruments for evaluating risk of bias across these prevalence studies, but heterogeneity in these studies by region of India, etc. is described in the Main Manuscript "Results" section, subsection on "Chest symptomatics who do not get evaluated at TB diagnostic facilities"	Note that there are no great instruments for evaluating risk of bias across these prevalence studies, but heterogeneity in these studies by region of India, etc. is described in the Main Manuscript "Results" section, subsection on "TB patients evaluated at diagnostic facilities who are not successfully diagnosed" and subsection on "Pretreatment loss to follow-up of smear-positive patients"	Note that there are no great instruments for evaluating risk of bias across these prevalence studies, but heterogeneity in these studies by region of India, etc. is described in the Main Manuscript "Results" section, subsection on "Post-treatment TB recurrence and death"
Additional analyses	16	Describe methods of additional analyses (e.g., sensitivity or subgroup analyses, meta-regression), if done, indicating which were pre-specified.	Not applicable	Not applicable	Not applicable
**RESULTS**					
Study selection	17	Give numbers of studies screened, assessed for eligibility, and included in the review, with reasons for exclusions at each stage, ideally with a flow diagram.	S2 Text (Fig A)	S3 Text (Fig C)	S4 Text (Fig D)
Study characteristics	18	For each study, present characteristics for which data were extracted (e.g., study size, PICOS, follow-up period) and provide the citations.	S2 Text (Table C)	S3 Text (Tables F-H)	S4 Text (Table K)

Risk of bias within studies	19	Present data on risk of bias of each study and, if available, any outcome level assessment (see item 12).	S2 Text (Table C)	S3 Text (Tables F-H)	S4 Text (Table K)
Results of individual studies	20	For all outcomes considered (benefits or harms), present, for each study: (a) simple summary data for each intervention group (b) effect estimates and confidence intervals, ideally with a forest plot.	Main manuscript Fig 2-4	Main manuscript Fig 5-6	Main manuscript "Results" section, subsection on "Post-treatment TB recurrence and death"
Synthesis of results	21	Present results of each meta-analysis done, including confidence intervals and measures of consistency.	Main manuscript Fig 2-4 and "Results" section, subsection on "Chest symptomatics who do not get evaluated at TB diagnostic facilities"	Main manuscript Fig 5-6 and "Results" section, subsection on "TB patients evaluated at diagnostic facilities who are not successfully diagnosed" and subsection on "Pretreatment loss to follow-up of smear-positive patients"	Main manuscript "Results" section, subsection on "Post-treatment TB recurrence and death"
Risk of bias across studies	22	Present results of any assessment of risk of bias across studies (see Item 15).	Note that there are no great instruments for evaluating risk of bias across these prevalence	Note that there are no great instruments for evaluating risk of bias across these prevalence studies, but	Note that there are no great instruments for evaluating risk of bias across these prevalence studies, but

			studies, but heterogeneity in these studies by region of India, etc. is described in the Main Manuscript "Results" section, subsection on "Chest symptomatics who do not get evaluated at TB diagnostic facilities"	heterogeneity in these studies by region of India, etc. is described in the Main Manuscript "Results" section, subsection on "TB patients evaluated at diagnostic facilities who are not successfully diagnosed" and subsection on "Pretreatment loss to follow-up of smear-positive patients"	heterogeneity in these studies by region of India, etc. is described in the Main Manuscript "Results" section, subsection on "Post-treatment TB recurrence and death"
Additional analysis	23	Give results of additional analyses, if done (e.g., sensitivity or subgroup analyses, meta-regression [see Item 16]).	Not applicable	Not applicable	Not applicable
**DISCUSSION**					
Summary of evidence	24	Summarize the main findings including the strength of evidence for each main outcome; consider their relevance to key groups (e.g., healthcare providers, users, and policy makers).	Main manuscript Fig 2-4 and "Results" section, subsection on "Chest symptomatics who do not get evaluated at TB diagnostic facilities" and the	Main manuscript Fig 5-6 and "Results" section, subsection on "TB patients evaluated at diagnostic facilities who are not successfully diagnosed"	Main manuscript "Results" section, subsection on "Post-treatment TB recurrence and death" and the

			"Discussion" section	and subsection on "Pretreatment loss to follow-up of smear-positive patients" and the "Discussion" section	"Discussion" section
Limitations	25	Discuss limitations at study and outcome level (e.g., risk of bias), and at review-level (e.g., incomplete retrieval of identified research, reporting bias).	Main manuscript Fig 2-4 and "Results" section, subsection on "Chest symptomatics who do not get evaluated at TB diagnostic facilities" and the "Discussion" section, subsection on "Limitations"	Main manuscript Fig 5-6 and "Results" section, subsection on "TB patients evaluated at diagnostic facilities who are not successfully diagnosed" and subsection on "Pretreatment loss to follow-up of smear-positive patients" and the "Discussion" section, subsection on "Limitations"	Main manuscript "Results" section, subsection on "Post-treatment TB recurrence and death" and the "Discussion" section, subsection on "Limitations"
Conclusions	26	Provide a general interpretation of the results in the context of other evidence, and implications for future research.	Main manuscript "Discussion" section	Main manuscript "Discussion" section	Main manuscript "Discussion" section
**FUNDING**					

Funding	27	Describe sources of funding for the systematic review and other support (e.g., supply of data); role of funders for the systematic review.	Main manuscript "Funding" section	Main manuscript "Funding" section	Main manuscript "Funding" section

From: Moher D, Liberati A, Tetzlaff J, Altman DG, The PRISMA Group (2009). Preferred Reporting Items for Systematic Reviews and Meta-Analyses: The PRISMA Statement. PLoS Med 6(6): e1000097. doi:10.1371/journal.pmed1000097

For more information, visit: **www.prisma-statement.org**.
