# Supplementary material for: The Tuberculosis Cascade of Care in India’s Public Sector: A Systematic Review and Meta-analysis
Source: PLoS Med. 2016 Oct 25;13(10):e1002149. doi: 10.1371/journal.pmed.1002149 (PMC5079571; doi:10.1371/journal.pmed.1002149)
Supplement: S2 Text — (PDF) [file pmed.1002149.s002.pdf]

S2 Text. Methods for the systematic review and meta-analysis of care-seeking by individuals in the community with cough >2 weeks (i.e., chest symptomatics) (Gap 1b)

Methods

Objectives

The objective of this systematic review and meta-analysis is to gain insight into Gap 1b in the TB cascade of care—the proportion of TB cases with access to government TB health services who do not seek care at health facilities. Specifically, we aim to extract data on three variables:

(1) the proportion of individuals with cough >2 weeks (i.e., "chest symptomatics) in India who report not having visited any medical provider after the onset of cough

(2) the proportion of individuals with cough >2 weeks in India who report not having seen a public sector healthcare provider after the onset of cough

(3) the proportion of individuals with cough >2 weeks in India who report not having received screening for TB with sputum smear microscopy

Search strategy

A medical librarian searched PubMed, Embase, Web of Science, and the Cochrane Register of Controlled Clinical Trials for studies published between January 1, 2000 and October 1, 2015, without language restrictions, using search terms for "tuberculosis", "tuberculosis symptoms", "India", and "healthcare-seeking behavior" (Table A). In addition, we carried out electronic searches of key Indian journals that may not be indexed in the above databases: the Indian Journal of Tuberculosis, Lung India, the Indian Journal of Chest and Allied Sciences, the India Journal of Public Health, and the Indian Journal of Community Medicine. Additional studies were identified by searching the reference lists of the primary studies and relevant review articles.

Inclusion and exclusion criteria

We included population-based, cross-sectional studies that assessed the healthcare-seeking behavior of individuals with cough >2 weeks or the healthcare-seeking behavior of individuals later confirmed to have TB. The studies also had to evaluate at least one of the three variables listed above. Health facility-based studies, studies with field research conducted prior to the year 2000, and studies with solely qualitative methods were excluded. In addition, studies with a sample size of fewer than 1000 individuals screened for chest symptoms were considered to be of very low quality and were excluded.

Study selection

Citations identified by the search were independently assessed by two reviewers (authors RS and RN) for their eligibility (Fig A). Disagreements between the two reviewers were resolved by discussion between RS and RN or, if necessary, through consultation of a third reviewer (S Satyanarayana).

Quality assessment

There are no well-recognized tools for evaluating the quality of the studies included in this systematic review. We therefore developed quality criteria relevant to population-based studies focused on identifying and interviewing chest symptomatics (Table B). We classified the population-based sampling strategy based on whether it was comprehensive, random, or convenience. Studies using convenience sampling were excluded from the review. Studies with a sample size of fewer than 1000 individuals screened for chest symptoms were excluded. One of the major challenges of population-based sampling is screening all members of a household, since men in particular may be working and out of the house at times when researchers are in the communities. Therefore, we also include the proportion of the estimated population screened and the proportion of identified chest symptomatics who were interviewed regarding care-seeking as additional quality indicators.

Data extraction and analysis

Two reviewers (RS and RN) independently extracted data from each included study into a structured data extraction form. Disagreements were resolved by discussion or, if necessary, by consulting a third reviewer (S Satyanarayana). From each study, we extracted information on the study design, location, setting (i.e., urban versus rural), sample size, and variables of interest (Table C). We also extracted information on 95% confidence intervals (95% CIs) where available; if 95% CIs were not reported, we calculated these from the data provided, assuming an infinite population size.

We generated Forest plots using Stata version 14 (College Station, TX, USA). We assume that each study finding represents the care-seeking behavior of patients in that location. India is a diverse country with substantial differences in the socioeconomic status and cultural practices of the patient population in every state. Therefore, we allow that the proportion is likely to vary from study to study, representing meaningful local differences.

Given these assumptions, we conducted the meta-analysis using a random effects model, and we performed meta-analysis even if there was substantial heterogeneity in the values in different studies. We report the pooled prevalence and heterogeneity (I^2) for the values in the included studies. The Forest plot and a narrative discussion of the results are included in the main text of this manuscript.

References

1. Charles N, Thomas B, Watson B, Raja Sakthivel M, Chandrasekeran V, Wares F. Care seeking behavior of chest symptomatics: a community based study done in South India after the implementation of the RNTCP. PLoS One. 2010;5(9):e12379. doi: 10.1371/journal.pone.0012379. PMID: 20862219.
2. Fochsen G, Deshpande K, Diwan V, Mishra A, Diwan VK, Thorson A. Health care seeking among individuals with cough and tuberculosis: a population-based study from rural India. Int J Tuberc Lung Dis. 2006;10(9):995-1000. PMID: 16964790.
3. George O, Sharma V, Sinha A, Bastian S, Santha T. Knowledge and behaviour of chest symptomatics in urban slum populations of two states in India towards care-seeking. Indian Journal of Tuberculosis. 2013;60:95-106.
4. Ghosh S, Sinhababu A, Taraphdar P, Mukhopadhyay DK, Mahapatra BS, Biswas AB. A study on care seeking behavior of chest symptomatics in a slum of Bankura, West Bengal. Indian J Public Health. 2010;54(1):42-4. doi: 10.4103/0019-557x.70553. PMID: 20859052.
5. Kar M, Logaraj M. Awareness, attitude and treatment seeking behaviour regarding tuberculosis in a rural area of Tamil Nadu. Indian J Tuberc. 2010;57(4):226-9. PMID: 21141344.
6. Satyanarayana S, Nair SA, Chadha SS, Sharma G, Yadav S, Mohanty S, et al. Health-care seeking among people with cough of 2 weeks or more in India. Is passive TB case finding sufficient? Public Health Action. 2012;2(4):157-61.
7. Suganthi P, Chadha VK, Ahmed J, Umadevi G, Kumar P, Srivastava R, et al. Health seeking and knowledge about tuberculosis among persons with pulmonary symptoms and tuberculosis cases in Bangalore slums. International Journal of Tuberculosis and Lung Disease. 2008;12(11):1268-73.
8. Thomas BE, Charles N, Watson B, Chandrasekaran V, Senthil Kumar R, Dhanalakshmi A, et al. Prevalence of chest symptoms amongst brick kiln migrant workers and care seeking behaviour: a study from South India. J Public Health (Oxf). 2014. doi: 10.1093/pubmed/fdu104. PMID: 25538142.

Figures

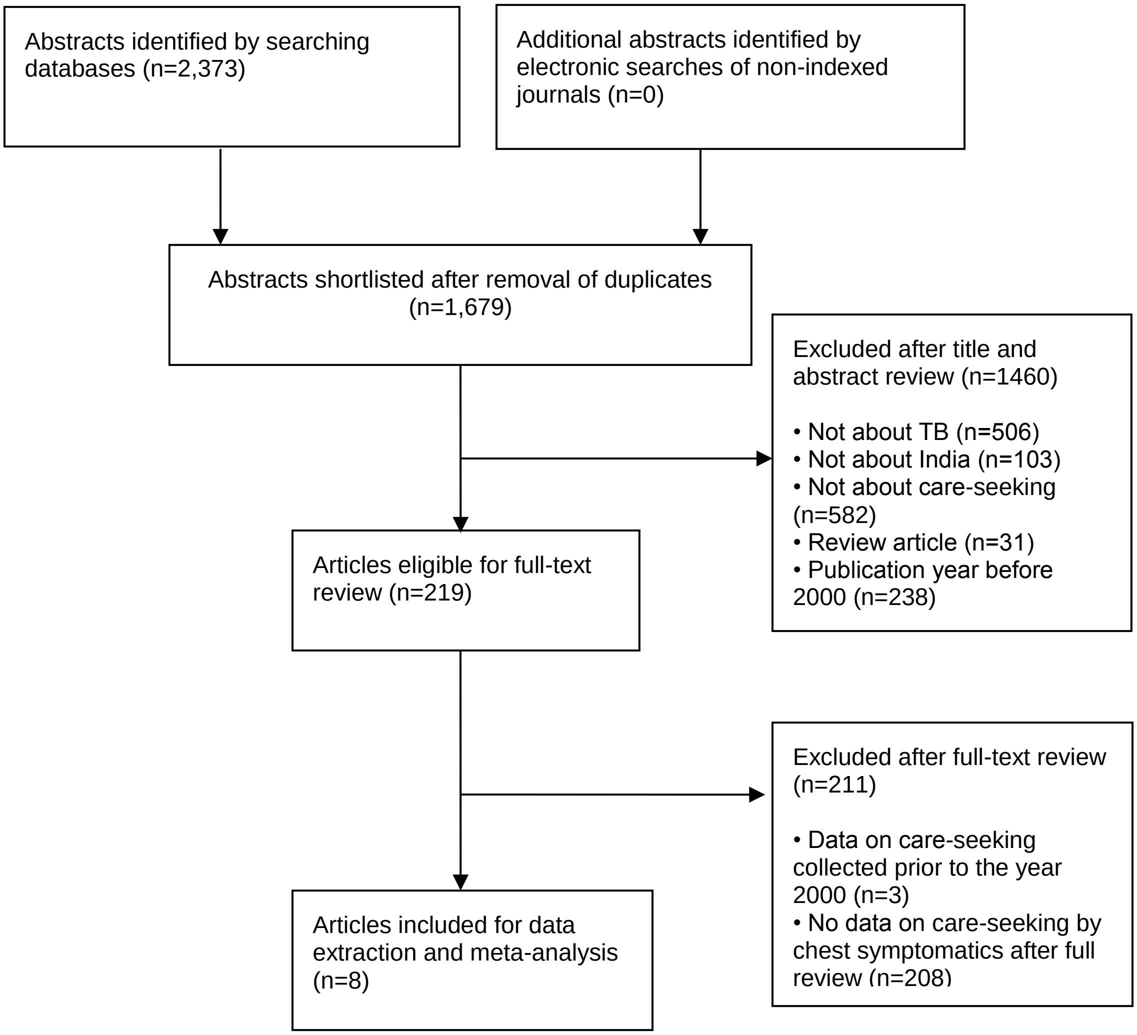

Fig A. Study selection for the systematic review of care-seeking behavior of individuals with cough >2-3 weeks (Gap 1b).

Tables

Table A. Search strategy to identify manuscripts regarding the health-seeking behavior of individuals with suspected TB (i.e., chest symptomatics) in India

**Terms for tuberculosis:**	“tuberculosis”[Mesh] OR *Mycobacterium tuberculosis*[tiab] OR TB[tiab]
**Terms for tuberculosis symptoms:**	chest symptomatic[tiab] OR chest symptomatics[tiab] OR suspects[tiab] OR chest symptoms[tiab] OR pulmonary symptoms[tiab] OR cough[tiab] OR respiratory symptoms[tiab]
**Terms for India:**	“India”[Mesh] OR India[tiab] OR India [ad] OR Indian [tiab] OR Indians [tiab]
**Terms for healthcare-seeking behavior:**	“patient acceptance of health care”[Mesh] OR healthcare seeking behavior[tiab]* OR care seeking[tiab] OR health behavior[tiab] OR behavior[tiab] OR “Health Services/utilization” [MeSH] OR referral[tiab] OR behaviour[tiab]* OR health behaviour[tiab] OR behaviour[tiab]

Table B. Criteria for assessing quality of care-seeking studies

Criterion	Quality level
**Sampling strategy**	
Random or comprehensive sampling of community residents or households	High
Convenience sampling of community residents or households	Low (exclude)
**Sample size**	
Multiple villages, slum settlements, or communities with 1000+ residents screened	High
Single village, slum settlement or community with 1000+ residents screened	Medium
<1000 residents screened	Low (exclude)
**Proportion of estimated population screened for cough**	
76-100% of screened	High
50-75% of screened	Medium
<50% of screened or not reported	Low
**Proportion of identified chest symptomatic who were interviewed about care seeking**	
76-100% interviewed	High
50-75% interviewed	Medium
<50% interviewed or not reported	Low

Table C. Characteristics of the included studies for the meta-analyses of chest-symptomatic care-seeking

Citation (year)	Location	Urban, rural, both, or unknown	Type of population	Number of community residents screened	Number of chest symptomatics identified	Non-response rate of chest symptomatics<br><br>N (%)	Chest symptomatics who have not seen any provider<br><br>N (%)	Chest symptomatics who have not seen a public sector provider<br><br>N (%)	Chest symptomatics who have not been evaluated with a sputum smear<br><br>N (%)
Charles (2010)[1]	Chennai and Madurai districts, Tamil Nadu	Urban and rural	General	18,417	640	5.3%	26.7%	63.4%	NR
Fochsen (2006)[2]	Ujjain district, Madhya Pradesh	Rural	General	45,719**	941	31.6%*	30.9%	77.2%	91%
George (2013)[3]	Four districts in Uttar Pradesh and Karnataka	Urban	Urban informal settlements	117,603**	3,041	NR*	33.3%	77.5%	92.8%
Ghosh (2010)[4]	Bankura district, West Bengal	Urban	An urban informal settlement*	1,156**	64	0%	25.0%	62.5%	NR
Kar (2010)[5]	Kancheepuram, Tamil Nadu	Rural	General	1,985**	65	0%	29.2%	61.5%	NR
Satyanarayana (2012)[6]	Thirty districts throughout India	Urban and rural	General	4,562**	437	0%	68.6%	NR	NR

Suganthi (2008)[7]	Bangalore, Karnataka	Urban	Urban informal settlements	9,676	166	25%*	50.8%	89.5%	97.2%
Thomas (2014)[8]	Tiruvallur district, Tamil Nadu	Rural	Villages with a large population of brick kiln workers	4,002**	377	0%	49.6%	89.9%	NR

*Medium or low quality for this indicator
**Proportion of the estimated population screened not reported (low quality)
NR=Not reported
