# Supplementary material for: The Tuberculosis Cascade of Care in India’s Public Sector: A Systematic Review and Meta-analysis
Source: PLoS Med. 2016 Oct 25;13(10):e1002149. doi: 10.1371/journal.pmed.1002149 (PMC5079571; doi:10.1371/journal.pmed.1002149)
Supplement: S3 Text — (PDF) [file pmed.1002149.s003.pdf]

S3 Text. Methods for the systematic review and meta-analysis of failure to complete the diagnostic workup and pretreatment loss to follow-up (Gaps 2 and 3)

Methods

Objectives

The objective of this systematic review and meta-analysis is to gain insight into Gaps 2 and 3 in the TB cascade of care—the proportion of TB cases presenting to government designated microscopy centers (DMCs) who fail to get diagnosed and the proportion of cases who are diagnosed with TB at government facilities but who do not get registered in treatment. Specifically, we aim to extract data on three variables:

(1) the proportion of patients evaluated at DMCs who submit one sputum sample but fail to submit a second sputum sample (sometimes referred to as "diagnostic default"). This value can be used to estimate the number of new smear-positive and retreatment smear-positive TB cases "missed" during the diagnostic workup (Gap 2).

(2) the proportion of TB patients with initial negative sputum smears who fail to complete the multi-step diagnostic workup for smear-negative TB (Fig B), especially chest X-ray evaluation (provides insights into Gap 2).

(3) the proportion of patients diagnosed with TB who fail to get registered and started on TB treatment, also known as "pretreatment loss to follow-up" (PTLFU) or "initial default" (Gap 3).

For the third aim, we should note that the Government of India's Revised National Tuberculosis Control Programme (RNTCP) has a relatively restrictive definition of PTLFU as consisting of smear-positive patients diagnosed in a given district, who are assumed to be living within the same district, but who are not initiated on treatment within the quarter (three-month calendar period) in which they were diagnosed. This definition has many shortcomings, especially the fact that many patients are diagnosed in one district but eventually migrate to another district to start treatment; these patients are excluded from the reporting of local PTLFU statistics.

Moreover, the RNTCP does not formally report PTLFU statistics in its annual report. Rather, we can only estimate this figure by calculating the difference between the number of smear-positive cases newly diagnosed at microscopy facilities and the number of smear-positive cases registered for treatment annually. This value may overestimate or underestimate the proportion of PTLFU cases. Therefore, we conduct our meta-analysis of studies that independently assess and more accurately report local PTLFU rates to help crosscheck this national estimate of PTLFU.

Search strategy

A medical librarian searched PubMed, Embase, Web of Science, and the Cochrane Register of Controlled Clinical Trials for studies published between January 1, 2000 and February 26, 2015, without language restrictions, using search terms for "tuberculosis", "India", and "loss to follow-up", including "pretreatment loss to follow-up" and "initial default" (Table D). In addition, we carried out electronic searches of key Indian journals that may not be indexed in the above databases: the Indian Journal of Tuberculosis, Lung India, the Indian Journal of Chest and Allied Sciences, the India Journal of Public Health, and the Indian Journal of Community

Medicine. Additional studies were identified by searching the reference lists of the primary studies and relevant review articles.

Inclusion and exclusion criteria

We included cross-sectional or cohort studies that audited records at government DMCs, tracked patients to understand attrition during the diagnostic workup for TB, or evaluated linkage of diagnosed smear-positive TB patients to care in the government sector. The studies had to evaluate at least one of the three variables listed above. Studies of private sector TB care, population-based studies, studies with field research conducted prior to the year 2000, and studies with solely qualitative methods were excluded. In addition, studies in which data were collected prior to the year 2000 (before the Government of India's DOT programme had broad coverage) were excluded. As per our quality criteria below, studies using convenience sampling or that include fewer than 150 patients were considered to be of very low quality and were also excluded from the meta-analysis.

Study selection

Citations identified by the search were independently assessed by two reviewers (authors RS and RN) for their eligibility (Fig C). Disagreements between the two reviewers were resolved by discussion between RS and RN or, if necessary, by consulting a third reviewer (S Satyanarayana).

Quality assessment

There are no well-recognized tools for evaluating the quality of studies included in systematic reviews of operational indicators within health systems (such as diagnostic default or pretreatment loss to follow-up). We created the following quality criteria relevant to the specific indicators being assessed in this systematic review (Table E):

(1) Studies in which a dedicated research team was used to track pretreatment loss to follow-up (PTLFU) patients were rated as being higher in quality than studies that rely on self-report by local TB programs to evaluate PTLFU.

(2) Studies that assess PTLFU and loss to follow-up during the diagnostic workup within a shorter time frame (e.g., within 1-2 months after diagnosis) were rated as being higher in quality than studies that assessed these indicators 3 or more months after diagnosis, since tracking patients to find out accurate outcomes becomes much harder with time due to patient mobility.

(3) Studies using convenience sampling or that include fewer than 150 patients were considered to be of very low quality and were excluded from the meta-analysis.

Notably, some of these quality criteria do not apply to evaluating of the proportion of people with suspected TB who fail to submit two sputum smears, since this indicator can be easily assessed through an audit of DMC records and does not require patient tracking.

Data extraction and analysis

Two reviewers (RS and RN) independently extracted the data from each included study into a structured data extraction form. Disagreements were resolved by consulting a third reviewer (S Satyanarayana). From each study, we extracted information on the study design, location,

setting (i.e., urban versus rural), sample size, study quality, and variables of interest (Tables F—H). We also extracted information on 95% confidence intervals (95% CIs) where available; if 95% CIs were not reported, we calculated these from the data provided, assuming an infinite population size.

We generated Forest plots for each variable for which data were available from at least five studies using Stata version 14 (College Station, TX, USA). We assumed that each study finding represents the local prevalence of a given indicator in that facility, city, or district in India. India is a diverse country with substantial differences in the quality of public sector services in every state. In addition, there are substantial differences in the socioeconomic status and cultural practices of the patient population in every state. Therefore, we allow that the proportion is likely to vary from study to study, representing meaningful local differences.

Given these assumptions, we conducted the meta-analyses for variables with data from 5 or more studies using a random effects model, and we performed meta-analysis even if there was substantial heterogeneity in the values in different studies. We report the pooled prevalence and heterogeneity (I^2) for the values from the included studies. The Forest plots and a narrative discussion of the results are included in the main text of this manuscript.

References

1. Sachdeva KS. Chapter 105: Management of tuberculosis: Indian guidelines. In: Muruganathan A, editor. Medicine Update 2013. Mumbai: The Association of Physicians of India; 2013.
2. Balasubramanian R, Garg R, Santha T, Gopi PG, Subramani R, Chandrasekaran V, et al. Gender disparities in tuberculosis: report from a rural DOTS programme in south India. Int J Tuberc Lung Dis. 2004;8(3):323-32. PMID: 15139471.
3. Chadha VK, Praseeja P, Hemanthkumar NK, Shivshankara BA, Sharada MA, Nagendra N, et al. Implementation efficiency of a diagnostic algorithm in sputum smear-negative presumptive tuberculosis patients. Int J Tuberc Lung Dis. 2014;18(10):1237-42. doi: 10.5588/ijtld.14.0218. PMID: 25216839.
4. Chandrasekaran V, Ramachandran R, Cunningham J, Balasubramanian R, Thomas A, Sudha G, et al. Factors leading to tuberculosis diagnostic drop-out and delayed treatment initiation in Chennai, India. Int J Tuberc Lung Dis. 2005;9(Supplement 1):S172.
5. Dandona R, Dandona L, Mishra A, Dhingra S, Venkatagopalakrishna K, Chauhan LS. Utilization of and barriers to public sector tuberculosis services in India. Natl Med J India. 2004;17(6):292-9. PMID: 15736548.
6. Rawat J, Biswas D, Sindhwani G, Kesharwani V, Masih V, Chauhan BS. Diagnostic defaulters: an overlooked aspect in the Indian Revised National Tuberculosis Control Program. J Infect Dev Ctries. 2012;6(1):20-2. PMID: 22240423.
7. Tripathy JP, Srinath S, Naidoo P, Ananthakrishnan R, Bhaskar R. Is physical access an impediment to tuberculosis diagnosis and treatment? A study from a rural district in North India. Public Health Action. 2013;3(3):235-9. doi: 10.5588/pha.13.0044. PMID: 26393036.
8. Sarkar J, Murhekar MV. Factors associated with low utilization of X-ray facilities among the sputum negative chest symptomatics in Jalpaiguri District (West Bengal) 2009. Indian J Tuberc. 2011;58:208-11.
9. Thomas A, Gopi PG, Santha T, Jaggarajamma K, Charles N, Prabhakaran E, et al. Course of action taken by smear negative chest symptomatics: a report from a rural area in South India. Indian J Tuberc. 2006;53:4-6.
10. Ahmed J, Chadha VK, Singh S, Venkatachalappa B, Kumar P. Utilization of RNTCP services in rural areas of Bellary District, Karnataka, by gender, age and distance from health centre. Indian J Tuberc. 2009;56(2):62-8. PMID: 19810587.
11. Chadha VK, Praseeja P, Gupta J, Ahmed J, Sharada MA, Srivastava R, et al. A descriptive study of tuberculosis case finding in private health care facilities in a South Indian district. Int J Tuberc Lung Dis. 2014;18(12):1455-8. doi: 10.5588/ijtld.14.0228. PMID: 25517811.
12. Dave P, Nimavat P, Shah A, Pujara K, Patel P, Modi B. Knowing more about initial default among diagnosed sputum smear-positive pulmonary tuberculosis patients in Gujarat, India [Abstract PC-868-03]. Int J Tuberc Lung Dis. 2013;17 (Suppl 2)(12):S469.
13. Dholakia YN. TB/ HIV coordination through Public Private Partnership: lessons from the field. Indian J Tuberc. 2013;60(1):23-7. PMID: 23540085.
14. Gopi PG, Chandrasekaran V, Subramani R, Narayanan PR. Failure to initiate treatment for tuberculosis patients diagnosed in a community survey and at health facilities under a DOTS program in a district of south India. Indian J Tuberc. 2005;52:153-6.
15. Khandekar J, Acharya AS, R TH, Sharma A. Do patients with tuberculosis referred from a tertiary care referral centre reach their peripheral health institution? Natl Med J India. 2013;26(6):332-4. PMID: 25073989.
16. Kumar S. A retrospective cohort study of the magnitude of initial default among sputum smear-positive TB patients diagnosed at NITRD New Delhi, 4th quarter 2012. India EIS Conferece; 2013 Nov 21-23; New Delhi, India.

17. Mandal A, Basu M, Das P, Mukherjee S, Das S, Roy N. Magnitude and reasons of initial default among new sputum positive cases of pulmonary tuberculosis under RNTCP in a district of West Bengal, India. South East Asia J of Public Health. 2015;4(1):41-7.
18. Mehra D, Kaushik RM, Kaushik R, Rawat J, Kakkar R. Initial default among sputum-positive pulmonary TB patients at a referral hospital in Uttarakhand, India. Trans R Soc Trop Med Hyg. 2013;107(9):558-65. doi: 10.1093/trstmh/trt065. PMID: 23920324.
19. Pillai D, Purty A, Prabakaran S, Singh Z, Soundappan G, Anandan V. Initial default among tuberculosis patients diagnosed in select medical colleges of Puducherry: issues and possible interventions. Int J Med Sci Public Health. 2015;4(7):1-4.
20. Sai Babu B, Satyanarayana AV, Venkateshwaralu G, Ramakrishna U, Vikram P, Sahu S, et al. Initial default among diagnosed sputum smear-positive pulmonary tuberculosis patients in Andhra Pradesh, India. Int J Tuberc Lung Dis. 2008;12(9):1055-8. PMID: 18713504.
21. Chadha SS, Sharath BN, Reddy K, Jaju J, Vishnu PH, Rao S, et al. Operational challenges in diagnosing multi-drug resistant TB and initiating treatment in Andhra Pradesh, India. PLoS One. 2011;6(11):e26659. doi: 10.1371/journal.pone.0026659. PMID: 22073182.
22. Shringarpure KS, Isaakidis P, Sagili KD, Baxi RK. Loss-To-Follow-Up on Multidrug Resistant Tuberculosis Treatment in Gujarat, India: The WHEN and WHO of It. PLoS One. 2015;10(7):e0132543. doi: 10.1371/journal.pone.0132543. PMID: 26167891.

Figures

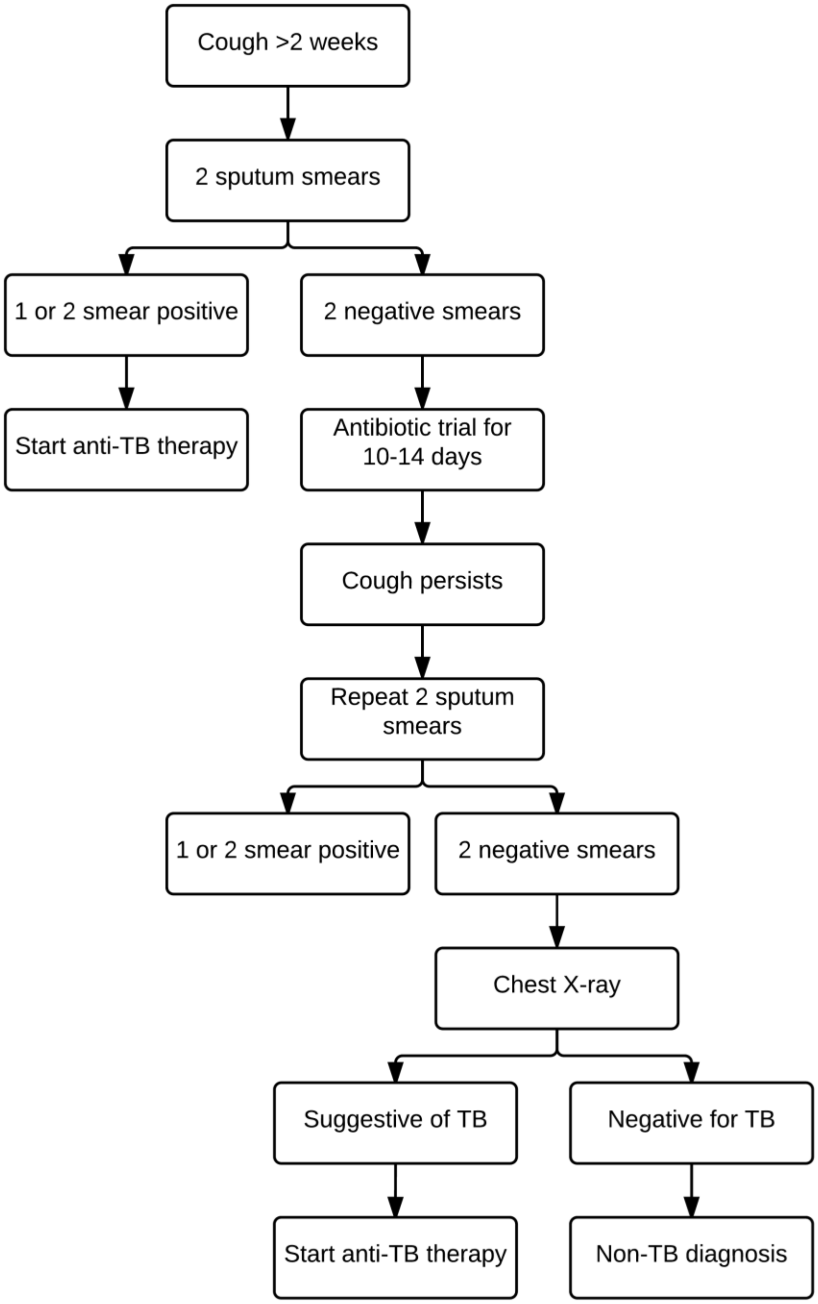

Fig B. Algorithm for diagnosing smear-negative tuberculosis in India's Revised National Tuberculosis Control Programme (RNTCP) [1]. This algorithm is currently undergoing revision by the RNTCP.

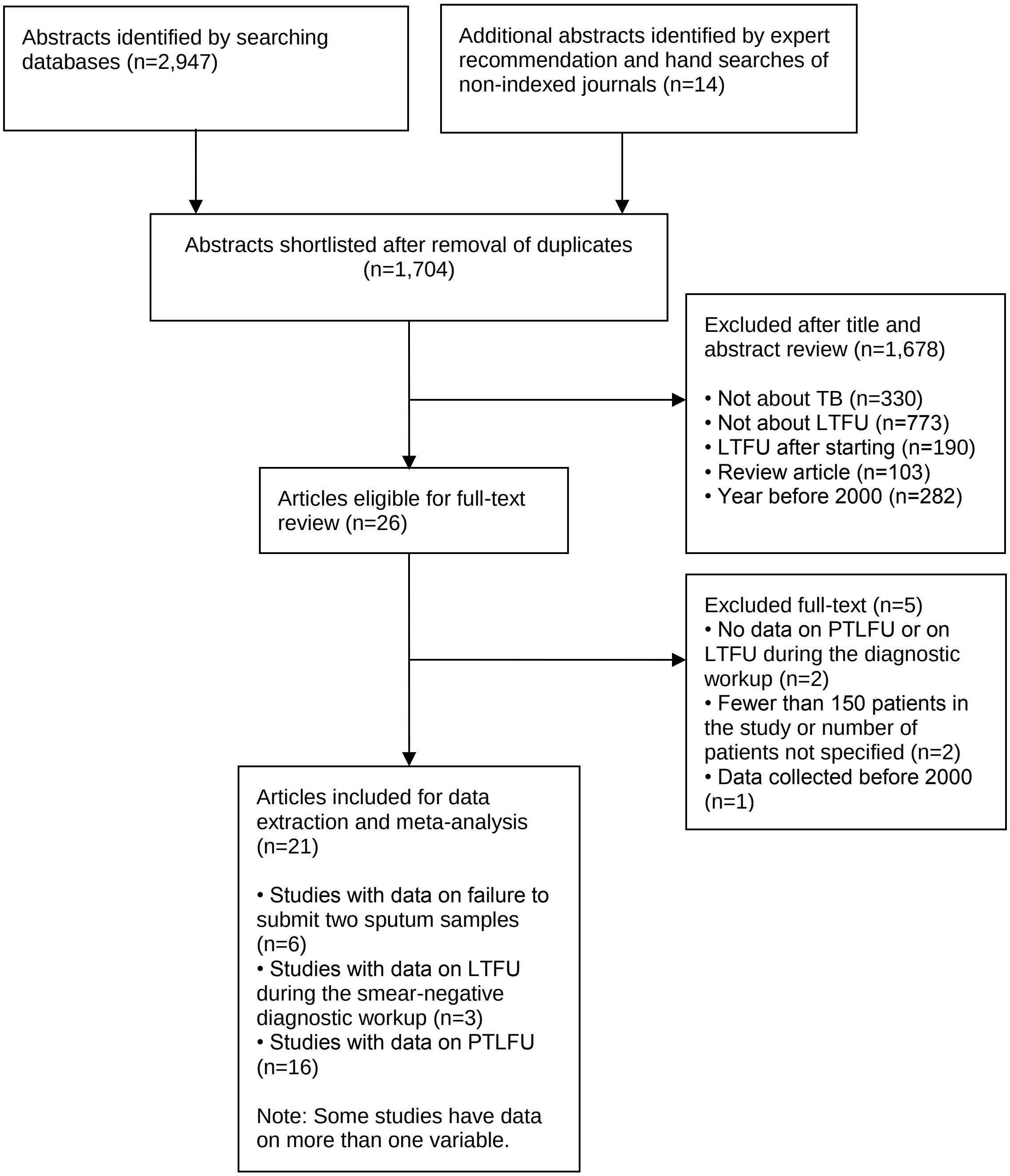

Fig C. PRISMA flowchart showing the selection process for studies evaluating pretreatment loss to follow-up or loss to follow-up during the diagnostic workup for tuberculosis. LTFU=loss to follow-up; PTLFU=pretreatment loss to follow-up; TB=tuberculosis.

Tables

Table D. Search strategy to identify manuscripts regarding pretreatment loss to follow-up, failure to complete the diagnostic workup, and loss to follow-up on treatment for TB patients in India

Terms for tuberculosis:	“tuberculosis”[Mesh] OR *Mycobacterium tuberculosis*[tiab] OR TB[tiab]
Terms for India:	“India”[Mesh] OR India[tiab] OR India[ad] OR Indian[tiab] OR Indians[tiab]
Terms for loss to follow-up:	"patient dropouts"[tiab] OR "treatment refusal"[mesh] OR "patient compliance"[Mesh] OR lost to follow up[tiab] OR loss to follow up[tiab] OR default*[tiab] OR compliance[tiab] OR adherence[tiab] OR noncompliance[tiab] OR nonadherence[tiab] OR patient cooperation[tiab] OR dropout*[tiab] OR linkage to care[tiab] OR retention[tiab] OR attrition[tiab] OR cascade of care[tiab] OR treatment cascade[tiab] OR treatment success*[tiab] OR treatment completion[tiab] OR cure[tiab] OR pretreatment loss to follow-up[tiab] OR initial default[tiab]

Table E. Criteria for assessing quality of studies evaluating pretreatment loss to follow-up or the proportion of patients who fail to complete the diagnostic workup for smear-negative TB

**Criteria**	**Quality level**
Sampling strategy	
Random or comprehensive sampling at selected facilities	High
Convenience sampling or not reported	Low (exclude)
Sample size	
>1 microscopy center and 150+ patients	High
Single center study with 150+ patients	Medium
<150 patients or not reported	Low (exclude)
Time frame of research fieldwork after patient diagnosis	
2 weeks to 1 month after diagnosis	High
1-3 months after diagnosis	Medium
>3 months after diagnosis or not reported	Low
Method of evaluating outcome	
Patient tracking by a dedicated research team	High
Relying on self-report by government TB program or not reported	Medium to Low

Table F. Characteristics of the included studies for the meta-analysis of the proportion of patients who fail to submit two sputum smears during initial evaluation

**Citation** (year)	**Location**	**Urban, rural, both, or unknown**	**Single or multiple designated microscopy centers** (DMCs)	**Sample size** (number of patients who submitted a single smear)	**Failed to submit a second or third sputum smear** N (%)
Balasubramanian (2004)[2]	Tiruvallur district, Tamil Nadu	Rural	7 DMCs	8,646	1,415 (16.4%)
Chadha (2014)[3]	Mysore, Shivamoga, and Chikmagalur districts, Karnataka	Urban and rural	22 DMCs	256	79 (30.9%)
Chandrasekaran (2005)[4]	Chennai city, Tamil Nadu	Urban	Numerous DMCs	1000	107 (10.7%)
Dandona (2004)[5]	Two districts each in Andhra Pradesh, Maharashtra, Rajasthan, and Tamil Nadu	Urban and rural	140 DMCs	83,099	4,646 (5.6%)
Rawat (2012)[6]	Dehradun, Uttarakhand	Urban	1 DMC (tertiary care hospital)*	2,349	175 (7.4%)
Tripathy (2013)[7]	Fatehgarh Sahib district, Punjab	Rural	6 DMCs	1,708	18 (1%)

*Low or medium study quality for the specific indicator

Table G. Characteristics of the included studies for the meta-analysis of the proportion of patients who fail to complete the multi-step smear-negative TB diagnostic workup

**Citation** (year)	**Location**	**Urban, rural, both, or unknown**	**Single or multiple designated microscopy centers** (DMCs)	**Methodology** (Patient tracking by a dedicated research team versus self-report by the government TB program)	**Time frame of research fieldwork after patient diagnosis**	**Sample size** (number of smear-negative patients evaluated whose symptoms did not subside)	**Failure to get screened with a chest X-ray—the final step of the diagnostic evaluation** N (%)
Chadha (2014)[3]**	Three districts in Karnataka	Urban and rural	22 DMCs	Dedicated research team	Not reported*	3	2 (66.7%)
Sarkar (2011)[8]	Jalpaiguri district, West Bengal	Rural	44 DMCs	Dedicated research team	Not reported*	4,875	2,962 (60.8%)
Thomas (2006)[9]	Tiruvallur district, Tamil Nadu	Urban and rural	17 DMCs	Dedicated research team	4 weeks	229	184 (80.3%)

*Low study quality for the specific indicator
**Study is excluded from the analysis due to small numbers

Table H. Characteristics of the included studies for the meta-analysis of the proportion of diagnosed smear-positive patients who are not registered and started on TB treatment (i.e., pretreatment loss to follow-up or initial default)

**Citation** (year)	**Location**	**Urban, rural, both, or unknown**	**Single or multiple diagnostic microscopy centers** (DMCs)	**Methodology** (Patient tracking by a dedicated research team versus self-report or audit of the government TB program)	**Time frame of research fieldwork after initial patient evaluation**	**Sample size** (number of patients diagnosed)	**Pretreatment loss to follow-up** N (%)
Studies of pretreatment loss to follow-up of smear-positive tuberculosis patients (Category I and II)							
Ahmed (2009)[10]	Bellary district, Karnataka	Rural	5 DMCs	Dedicated research team	4 weeks	232	43 (18.5%)
Balasubramanian (2004)[2]	Tiruvallur district, Tamil Nadu	Rural	7 DMCs	Dedicated research team	12 weeks*	833	120 (14.4%)
Chadha (2014)[11]	Tumkur district, Karnataka	Urban and rural	28 DMCs	Dedicated research team	Not reported*	940	103 (11.0%)
Dandona (2004)[5]	Two districts each in Andhra Pradesh, Maharashtra, Rajasthan, and Tamil Nadu	Urban and rural	140 DMCs	Not reported*	Not reported*	12,287	3,846 (31.3%)
Dave (2013)[12]	Entire state of Gujarat	Urban and rural	Numerous DMCs	Not reported*	Not reported*	65,010	3,159 (4.9%)
Dholakia (2013)[13]	Four districts in Maharashtra	Urban and rural	164 DMCs	Dedicated research team	Not reported*	5,062	842 (16.6%)

Gopi (2005)[14]	Tiruvallur district, Tamil Nadu	Rural	17 DMCs	Dedicated research team	8 weeks	1,049	156 (14.9%)
Khandekar (2013)[15]	Delhi	Urban	1 DMC (tertiary care hospital)*	Dedicated research team	Not reported*	1,361	63 (4.6%)
Kumar (2013)[16]	New Delhi	Urban	1 DMC (tertiary care hospital)*	Not reported*	Not reported*	184	39 (21.2%)
Mandal (2015)[17]	Darjeeling district, West Bengal	Urban and rural	2 DMCs	Dedicated research team	Not reported*	562	132 (23.5%)
Mehra (2013)[18]	Dehradun city, Uttarakhand	Urban	1 DMC (tertiary care hospital)*	Dedicated research team	2 weeks	555	120 (21.6%)
Pillai (2015)[19]	Puducherry	Urban	4 DMCs (tertiary care hospitals)	Dedicated research team	Not reported*	950	145 (15.3%)
Sai Babu (2008)[20]	20 districts in Andhra Pradesh	Urban and rural	Numerous DMCs	Self-report by the government TB program*	12 weeks or more after diagnosis*	15,361	685 (4.5%)
Tripathy (2013)[7]	Fatehgarh Sahib district, Punjab	Rural	6 DMCs	Dedicated research team	1 week after diagnosis	156	44 (28.2%)
Studies of pretreatment loss to follow-up of multidrug resistant tuberculosis (MDR TB) patients							
Chadha (2011)[21]	Four districts in Andhra Pradesh	Urban and rural	Numerous DMCs	Audit of the government TB program*	Two or more years after diagnosis*	169	57 (33.7%)
Shringarpure (2015)[22]	Vadodara, Gujarat (received patients from six districts)	Urban and rural	1 DMC (tertiary care hospital)*	Audit of the government TB program*	Not reported	4321	977 (22.6%)

*Low or medium study quality for the specific indicator
