# Supplementary material for: The Tuberculosis Cascade of Care in India’s Public Sector: A Systematic Review and Meta-analysis
Source: PLoS Med. 2016 Oct 25;13(10):e1002149. doi: 10.1371/journal.pmed.1002149 (PMC5079571; doi:10.1371/journal.pmed.1002149)
Supplement: S4 Text — (PDF) [file pmed.1002149.s004.pdf]

S4 Text. Methods for the systematic review and meta-analysis of studies evaluating tuberculosis recurrence or death after treatment completion or cure (Gap 5)

Methods

Objectives

The objective of this systematic review and meta-analysis is to gain insight into Gap 5 in the TB cascade of care—the proportion of TB patients who achieve cure or treatment completion who subsequently experience TB recurrence or die within the first year after completing treatment.

Search strategy

A medical librarian searched PubMed, Embase, Web of Science, and the Cochrane Register of Controlled Clinical Trials for studies published between January 1, 2000 and October 9, 2015, without language restrictions, using search terms for “tuberculosis”, “India”, and “recurrence” (Table I). In addition, we carried out electronic searches of key Indian journals that may not be indexed in the above databases: the Indian Journal of Tuberculosis, Lung India, the Indian Journal of Chest and Allied Sciences, the India Journal of Public Health, and the Indian Journal of Community Medicine. Additional studies were identified by searching the reference lists of the primary studies and relevant review articles.

Inclusion and exclusion criteria

We included studies of TB patients in government clinics in India who achieved either cure (for smear-positive patients) or treatment completion (for other types of TB) who were subsequently followed-up for a time period between 12 to 24 months after treatment completion to determine the rate of TB recurrence and death from any cause. We included prospective and retrospective cohort studies that evaluated this outcome in a “real world” setting.

Any studies of TB recurrence during a clinical trial were excluded, as these studies tend to be testing out new interventions or medications that may not be standard of care within the RNTCP. They also tend to have substantially higher rates of medication adherence and lower patient loss to follow-up. Studies with field research conducted prior to the year 2000 and with solely qualitative methods were excluded. We also excluded studies of TB recurrence that have follow-up time periods that are not between 12 to 24 months after the completion of therapy.

Study selection

Citations identified by the search were assessed by one reviewer (author RS) for their eligibility (Fig D). If the inclusion or exclusion status of a manuscript was unclear, two coauthors were consulted (RN and S Satyanarayana).

Quality assessment

There are no well-recognized tools for evaluating the quality of studies included in this systematic review. We therefore developed quality criteria relevant to studies of TB recurrence (Table J). We classified the sampling strategy based on whether it was comprehensive, random, or convenience. Studies using convenience sampling were excluded from the review. We considered studies that evaluated patient outcomes at multiple TB treatment centers to be higher in quality than single-center studies, since they may better represent the outcomes in a

local area. Studies with fewer than 100 patients in the sample were considered to be of very low quality and were excluded.

With regard to study methodology, we consider prospective cohort studies and studies using active surveillance to identify TB recurrence to be higher in quality than retrospective cohort studies and those employing passive surveillance to identify cases of TB recurrence. Finally, we consider studies that screened all symptomatic patients with sputum smear microscopy and mycobacterial culture to diagnose TB recurrence to be higher in quality than studies that diagnosed recurrence using only sputum smear microscopy or clinical diagnosis alone.

Data extraction and analysis

Two reviewers (RS and RN) independently extracted the data from each included study into a structured data extraction form. Disagreements were resolved by consulting a third reviewer (S Satyanarayana). From each study, we extracted information on the study design, location, setting (i.e., urban versus rural), sample size, and variables of interest (Table K). We also extracted information on 95% confidence intervals (95% CIs) where available; if 95% CIs were not reported, we calculated these from the data provided, assuming an infinite population size.

We generated Forest plots for each variable for which data were available from at least five studies using Stata version 14 (College Station, TX, USA). We assume that each study finding represents the local prevalence of each value in that facility, city, or district in India. India is a diverse country with substantial differences in the quality of public sector services and in the socioeconomic status and cultural practices of people in every state. Therefore, we allow that the proportion is likely to vary from study to study, representing meaningful local differences.

Given these assumptions, we conducted the meta-analysis for variable with data from 5 or more studies using a random effects model. We performed meta-analysis even if there was substantial heterogeneity in the values in different studies. We report the pooled prevalence and heterogeneity (I^2) for the values from the included studies, if formal meta-analysis was conducted. A narrative discussion of the results is included in the main text of this manuscript.

References

1. Mehra RK, Dhingra VK, Nish A, Vashist RP. Study of relapse and failure cases of CAT I retreated with CAT II under RNTCP--an eleven year follow up. Indian J Tuberc. 2008;55(4):188-91. PMID: 19295105.
2. Mahishale V, Patil B, Lolly M, Eti A, Khan S. Prevalence of Smoking and Its Impact on Treatment Outcomes in Newly Diagnosed Pulmonary Tuberculosis Patients: A Hospital-Based Prospective Study. Chonnam Med J. 2015;51(2):86-90. doi: 10.4068/cmj.2015.51.2.86. PMID: 26306303.
3. Swaminathan S, Deivanayagam CN, Rajasekaran S, Venkatesan P, Padmapriyadarsini C, Menon PA, et al. Long term follow up of HIV-infected patients with tuberculosis treated with 6-month intermittent short course chemotherapy. Natl Med J India. 2008;21(1):3-8. PMID: 18472696.
4. Tripathy S, Anand A, Inamdar V, Manoj MM, Khillare KM, Datye AS, et al. Clinical response of newly diagnosed HIV seropositive & seronegative pulmonary tuberculosis patients with the RNTCP Short Course regimen in Pune, India. Indian J Med Res. 2011;133:521-8. PMID: 21623038.
5. Thomas A, Gopi PG, Santha T, Chandrasekaran V, Subramani R, Selvakumar N, et al. Predictors of relapse among pulmonary tuberculosis patients treated in a DOTS programme in South India. Int J Tuberc Lung Dis. 2005;9(5):556-61. PMID: 15875929.
6. Sadacharam K, Gopi PG, Chandrasekaran V, Eusuff SI, Subramani R, Santha T, et al. Status of smear-positive TB patients at 2-3 years after initiation of treatment under a DOTS programme. Indian J Tuberc. 2007;54(4):199-203. PMID: 18072535.
7. Dandekar RH, Jagannath VD. The fate of tuberculosis cases after two years of DOTS chemotherapy in Aurangabad city, Maharasthra. Natl J Community Med. 2014;5(2):174-8.
8. Prasad R, Verma SK, Shrivastava P, Kant S, Kushwaha RA, Kumar S. A follow up study on Revised National Tuberculosis Control Programme (RNTCP): results from a single centre study. Lung India. 2008;25(4):142-4. doi: 10.4103/0970-2113.45277. PMID: 21264079.
9. Vashishtha R, Mohan K, Singh B, Devarapu SK, Sreenivas V, Ranjan S, et al. Efficacy and safety of thrice weekly DOTS in tuberculosis patients with and without HIV co-infection: an observational study. BMC Infect Dis. 2013;13:468. doi: 10.1186/1471-2334-13-468. PMID: 24099345.
10. Sharma SK, Solanki R, Mohan A, Jain NK, Chauhan LS. Outcomes of Category III DOTS treatment in immunocompetent patients with tuberculosis pleural effusion. Int J Tuberc Lung Dis. 2012;16(11):1505-9. doi: 10.5588/ijtld.12.0233. PMID: 23044446.
11. Sharma SK, Soneja M, Prasad KT, Ranjan S. Clinical profile & predictors of poor outcome of adult HIV-tuberculosis patients in a tertiary care centre in north India. Indian J Med Res. 2014;139(1):154-60. PMID: 24604050.

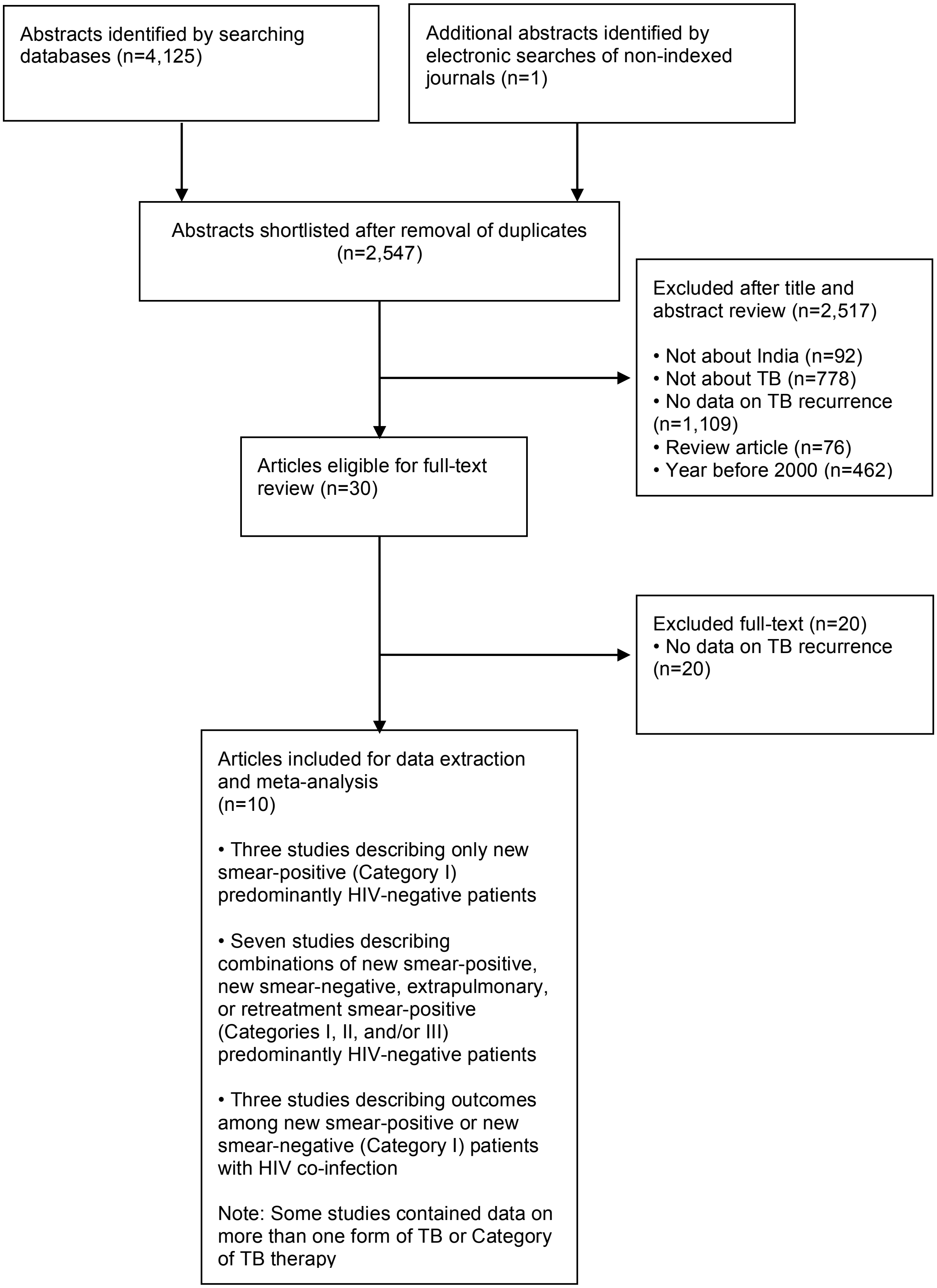

Fig D. PRISMA flowchart showing the selection process for studies evaluating post-treatment TB recurrence or death. TB=tuberculosis.

Table I. Search strategy to identify manuscripts describing recurrence rates among patients who completed TB treatment in India

**Terms for tuberculosis:**	"tuberculosis"[Mesh] OR *Mycobacterium tuberculosis*[tiab] OR TB[tiab]
**Terms for India:**	"India"[Mesh] OR India[tiab] OR India[ad] OR Indian[tiab] OR Indians[tiab]
**Terms for recurrence:**	recurrence[Mesh] OR relapse[tiab] OR recrudescence[tiab] OR reinfection[tiab] OR follow-up[tiab] OR failure[tiab]

Table J. Criteria for assessing quality of TB recurrence studies

Criteria	Quality level
**Sampling strategy**	
Random or comprehensive sampling of patients	High
Random or comprehensive sampling of patients but <50% of cohort retained in follow-up	Low (exclude)
Convenience sampling	Low (exclude)
**Sample size and distribution**	
>1 treatment center and 100+ patients	High
Single treatment center study with 100+ patients	Medium
<100 patients	Low (exclude)
**Cohort design and surveillance for recurrence**	
Active surveillance (prospective)	High
Passive surveillance (generally retrospective)	Medium to low
**Method for diagnosing recurrence**	
Sputum smear and mycobacterial culture	High
Sputum smear and clinical diagnosis without culture	Medium
Clinical diagnosis alone without microbiological tests	Low

Table K. Characteristics of the included studies for the meta-analyses of the proportion of TB patients who complete TB treatment but subsequently experience TB recurrence or death

Citation (year)	Location	Urban, rural, both, or not reported	Methodology	Sampling strategy<br><br>(Percent of patients who achieved cure or treatment completion but lost to follow-up before final relapse or death outcome assessed)	Treatment categories included in the cohort	Types of TB<br><br>(smear-positive pulmonary, smear-negative pulmonary, extrapulmonary)	Sample size<br><br>(Number of patients who achieved cure or treatment completion and followed-up for the full study period)	Patients with TB recurrence between 12 to 24 months<br><br>N (%)	Patients who died between 12 to 24 months<br><br>N (%)	Patients with an unfavorable outcome – death or recurrence<br><br>N (%)
Cohorts with category I smear-positive pulmonary tuberculosis patients only (predominantly HIV-negative)										
Mehra (2008)[1]	Delhi	Urban	Retrospective	Comprehensive (LFU not reported)	Category I	Smear-positive pulmonary	408	29 (7.1)	Not reported	Not reported
Sadacharam[a] (2007)[6]	Tiruvallur, Tamil Nadu	Rural	Retrospective	Comprehensive (11.2% LFU)	Category I	Smear-positive pulmonary	646	75 (11.6)	39 (4.4)	114 (17.6)
Thomas[a] (2005)[5]	Tiruvallur, Tamil Nadu	Rural	Prospective	Comprehensive (4.3% LFU)	Category I	Smear-positive pulmonary	511	62 (12.1)	12 (2.3)	74 (14.5)
Cohorts with other categories and types of tuberculosis (predominantly HIV-negative)										
Dandekar[b] (2014)[7]	Aurangabad, Maharashtra	Urban	Retrospective	Stratified random (16% LFU)	Category I and II	Smear-positive pulmonary, smear-negative pulmonary, extrapulmonary	276	9 (3.3)	23 (8.3)	32 (11.6)
Mahishale (2015)[2]	Belgaum, Karnataka	Urban	Prospective	Comprehensive (LFU not reported)	Category I	Smear-positive pulmonary, smear-negative pulmonary	2038	158 (7.8)	Not reported	Not reported

Prasad[b] (2008)[8]	Lucknow, Uttar Pradesh	Urban	Prospective	Comprehensive (28.3% LFU)	Category I, II, and III	Not reported (presumably all forms of TB)	152	11 (7.1)	4 (2.6)	15 (9.7)
Sadacharam (2007)[6]	Tiruvallur, Tamil Nadu	Rural	Retrospective	Comprehensive (11.2% LFU)	Category II	Smear-positive pulmonary	88	19 (21.6)	5 (5.7)	24 (27.3)
Sharma (2012)[10]	Delhi	Urban	Prospective	Comprehensive (13% LFU)	Category III	Tuberculous pleural effusion (extrapulmonary)	261	2 (0.8)	Not reported	Not reported
Tripathy[b] (2011)[4]	Pune, Maharashtra	Urban	Prospective	Comprehensive (13.8% LFU)	Category I and III	Smear-positive pulmonary, smear-negative pulmonary	138	7 (5.1)	4 (2.9)	11 (8.0)
Vashishtha[b] (2013)[9]	Delhi	Urban	Prospective	Comprehensive (11.7% LFU)	Category I	Smear-positive pulmonary, smear-negative pulmonary, extrapulmonary	128	2 (1.6)	1 (0.8)	3 (2.3)
Cohorts of HIV/TB co-infected patients										
Swaminathan (2008)[3]	Chennai, Tamil Nadu	Urban	Prospective	Comprehensive (0% LFU)	Category I	Smear-positive pulmonary	31*	12 (38.7)	7 (22.6)	19 (61.3)
Tripathy (2011)[4]	Pune, Maharashtra	Urban	Prospective	Comprehensive (9.8% LFU)	Category I	Smear-positive pulmonary, smear-negative pulmonary	83*	2 (2.4)	32 (38.6)	34 (41.0)
Vashishtha (2013)[9]	Delhi	Urban	Prospective	Comprehensive (9.8% LFU)	Category I	Smear-positive pulmonary, smear-negative pulmonary, extrapulmonary	109*	1 (0.9)	9 (8.3)	10 (9.1)

Sharma (2014)[11]	Delhi	Urban	Retrospective	Comprehensive (LFU not reported)	Category I	Smear-positive, smear-negative, extrapulmonary	332	12 (3.6)	Not reported	Not reported

*Note that for cohorts with HIV-infected TB patients we present cohorts with fewer than 100 patients in the table followed up because of the paucity of data in this area; however, we do not meta-analyze these findings.

[a]Included in the meta-analysis of studies evaluating recurrence of TB in new smear-positive patients treated with Category I therapy

[b]Included in the meta-analysis of studies evaluating recurrence of TB in new smear-negative, extrapulmonary, and retreatment other patients
