# Supplementary material for: The Tuberculosis Cascade of Care in India’s Public Sector: A Systematic Review and Meta-analysis
Source: PLoS Med. 2016 Oct 25;13(10):e1002149. doi: 10.1371/journal.pmed.1002149 (PMC5079571; doi:10.1371/journal.pmed.1002149)
Supplement: S5 Text — (PDF) [file pmed.1002149.s005.pdf]

S5 Text: Calculation of "steps" and "gaps" for the tuberculosis cascade of care in India, 2013

Step 1: Number of prevalent TB cases in India in 2013

Overall cascade (all forms of TB) – Step 1:

Overall prevalence of TB (all forms) for 2013 based on the 2015 WHO revision of this estimate [1, 2] = 2,700,000 (95%CI: 1,800,000—3,800,000)

Gap 1a/1b: Number of TB patients who do not reach public sector TB diagnostic facilities

Overall cascade (all forms of TB) – Gap 1a/1b:

Assumption 1: The total number of prevalent TB patients in India per WHO estimates for 2013 (Step 1) [1, 2] = 2,700,000 (95%CI:1,800,000—3,800,000)

Assumption 2: The total number of TB patients evaluated at RNTCP (public sector) TB diagnostic facilities in 2013 (Step 2) = 1,938,027 (95%CI:1,840,748—2,050,001)

Overall cascade Gap 1a/1b estimate = Overall cascade Step 1 estimate – Overall cascade Step 2 estimate = 2,700,000 – 1,938,027 = 761,973

Overall cascade Gap 1a/1b lower bound = Overall cascade Step 1 lower bound – Overall cascade Step 2 upper bound = 1,800,000 – 2,050,001 = -250,001

Overall cascade Gap 1a/1b upper bound = Overall cascade Step 1 upper bound – Overall cascade Step 2 lower bound = 3,800,000 – 1,840,748 = 1,959,252

Step 2: Number of TB cases evaluated at TB diagnostic facilities

New smear-positive patients – Step 2:

Assumption 1: Proportion of patients who fail to provide a second sputum specimen = 0.11 (95%CI:0.07—0.15) (value is drawn from the meta-analysis results presented in Fig 5 of the main manuscript)

Assumption 2: Incremental yield of a second sputum smear for diagnosing smear-positive TB = 0.119 (confidence interval not provided in source study [3])

Assumption 3: Smear-positive patients presenting to TB diagnostic facilities who remain undiagnosed because of failure to provide a second sputum smear = 0.119 X 0.11 (95%CI:0.07—0.15) = 0.013 (95%CI:0.008—0.018)

Assumption 4: Number of new smear-positive patients diagnosed (Step 3) = 720,617

New smear-positive Step 2 estimate = 720,617 / (1 – 0.013) = 730,108

New smear-positive Step 2 lower bound = 720,617 / (1 – 0.008) = 726,428

New smear-positive Step 2 upper bound = 720,617 / (1 – 0.018) = 733,826

Retreatment smear-positive patients – Step 2:

Assumption 1: Proportion of patients who fail to provide a second sputum specimen = 0.11 (95%CI:0.07—0.15)

Assumption 2: Incremental yield of a second sputum smear for diagnosing smear-positive TB = 0.119 (confidence interval not provided in source study [3])

Assumption 3: Smear-positive patients presenting to TB diagnostic facilities who remain undiagnosed because of failure to provide a second sputum smear = 0.119 X 0.11 (95%CI:0.07—0.15) = 0.013 (95%CI:0.008—0.018)

Assumption 4: Number of retreatment smear-positive patients diagnosed (Step 3)=207,573

Retreatment smear-positive Step 2 estimate = 207,573 / (1 – 0.013) = 210,307

Retreatment smear-positive Step 2 lower bound = 207,573 / (1 – 0.008) = 209,247

Retreatment smear-positive Step 2 upper bound = 207,573 / (1 – 0.018) = 211,378

New smear-negative patients – Step 2:

Assumption 1: In a recent study of the implementation of Xpert® MTB/Rif at 18 geographically diverse sites in India, Xpert® diagnosed 9600 smear-positive TB patients and 4,524 smear-negative TB patients [4].

Assumption 2: The sensitivity of Xpert® MTB/Rif for diagnosing smear-negative TB based on a recent meta-analysis is 0.67 (95%CI: 0.60—0.74) [5].

Assumption 3: Among all true TB patients being evaluated at designated microscopy centers in India, we estimated the ratio of smear-positive to smear-negative TB patients as follows:

(a) We estimate that if 4,524 smear-negative TB patients were diagnosed by Xpert® MTB/Rif in the multisite study in India [4], then 4,524 / 0.67 (95%CI: 0.60—0.74) = 6,752 (95%CI: 6,114—7,540) true smear-negative TB patients must have reached TB diagnostic facilities and been evaluated at these 18 sites in India.

(b) Based on this estimate of the number of smear-negative TB patients evaluated at these 18 sites, we estimate that the ratio of smear-positive to smear-negative TB patients who reach RNTCP centers and are evaluated for TB as follows:

Ratio of smear-positive:smear-negative TB estimate = 9,600:6,752 = 1.42:1

Ratio of smear-positive:smear-negative TB lower bound = 9,600:7,540 = 1.27 → we then adjust this estimated lower bound by assuming an infinite population size for this proportion, which suggests a confidence interval for this ratio of 1:27 (95%CI:1.26:1—1.29:1). We therefore use **1:26:1** as the lower bound estimate for this ratio.

Ratio of smear-positive: smear-negative TB upper bound = 9,600:6,114 = 1.57 → we then adjust this estimated upper bound by assuming an infinite population size for this proportion, which suggests a confidence interval for this ratio of 1.57:1 (95%CI: 1.55:1—1.59:1). We therefore use **1.59:1** as the upper bound estimate for this ratio.

Assumption 4: Number of smear-positive TB patients evaluated at diagnostic facilities (Step 2) = 730,108 (95%CI: 726,428—733,826)

New smear-negative Step 2 estimate = 730,108 / 1.42 = 514,161

New smear-negative Step 2 lower bound = 726,428 / 1.59 = 456,873

New smear-negative Step 2 upper bound = 733,826 / 1.26 = 582,402

Retreatment smear-negative patients – Step 2:

Assumption 1: In a recent study of the implementation of Xpert® MTB/Rif at 18 geographically diverse sites in India, Xpert® diagnosed 9600 smear-positive TB patients and 4,524 smear-negative TB patients [4].

Assumption 2: The sensitivity of Xpert® MTB/Rif for diagnosing smear-negative TB based on a recent meta-analysis is 0.67 (95%CI: 0.60—0.74) [5].

Assumption 3: Among all true TB patients being evaluated at designated microscopy centers in India, we estimated the ratio of smear-positive to smear-negative TB patients as follows:

(a) We estimate that if 4,524 smear-negative TB patients were diagnosed by Xpert® MTB/Rif in the multisite study in India [4], then 4,524 / 0.67 (95%CI: 0.60—0.74) = 6,752 (95%CI: 6,114—7,540) true smear-negative TB patients must have reached TB diagnostic facilities and been evaluated at these 18 sites in India.

(b) Based on this estimate of the number of smear-negative TB patients evaluated at these 18 sites, we estimate that the ratio of smear-positive to smear-negative TB patients who reach RNTCP centers and are evaluated for TB as follows:

Ratio of smear-positive:smear-negative TB estimate = 9,600:6,752 = 1.42:1

Ratio of smear-positive:smear-negative TB lower bound = 9,600:7,540 = 1.27 → we then adjust this estimated lower bound by assuming an infinite population size for this proportion, which suggests a confidence interval for this ratio of 1:27 (95%CI:1.26:1—1.29:1). We therefore use **1:26:1** as the lower bound estimate for this ratio.

Ratio of smear-positive: smear-negative TB upper bound = 9,600:6,114 = 1.57 → we then adjust this estimated upper bound by assuming an infinite population size for this proportion, which suggests a confidence interval for this ratio of 1.57:1 (95%CI: 1.55:1—1.59:1). We therefore use **1.59:1** as the upper bound estimate for this ratio.

Assumption 5: Number of retreatment smear-positive TB patients evaluated at diagnostic facilities (Step 2) = 210,307 (95%CI:209,247—211,378)

Retreatment smear-negative Step 2 estimate = 210,307 / 1.42 = 148,104

Retreatment smear-negative Step 2 lower bound = 209,247 / 1.59 = 131,602

Retreatment smear-negative Step 2 upper bound = 211,378 / 1.26 = 167,760

Extrapulmonary TB patients – Step 2:

Assumption 1: We estimate the proportion of extrapulmonary TB patients who remain undiagnosed despite presenting to a RNTCP TB diagnostic facility (Gap 2) by taking the average of the proportion of undiagnosed smear-positive TB patients (Gap 2) and the proportion of undiagnosed smear-negative TB patients (Gap 2).

Assumption 2: The proportion of undiagnosed smear-positive patients = 0.013 (95%CI:0.008–0.018)

Assumption 3: The proportion of undiagnosed smear-negative patients was estimated to be 0.376 (95%CI:0.293—0.453) as follows:

(a) Proportion of undiagnosed smear-negative patients estimate = (Smear-negative Step 2 estimate – Smear-negative Step 3 estimate) / Smear-negative Step 2 estimate = (514,161 – 320,982) / 514,161 = 0.376

(b) Proportion of undiagnosed smear-negative patients upper bound = (Smear-negative Step 2 upper bound – Smear-negative Step 3 lower bound) / Smear-negative Step 2 upper bound = (582,402 – 318,491) / 582,402 = 0.453

(c) Proportion of undiagnosed smear-negative patients lower bound = (Smear-negative Step 2 lower bound – Smear-negative Step 3 upper bound) / Smear-negative Step 3 lower bound = (456,873 – 323,148) / 456,873 = 0.293

Assumption 4: The proportion of undiagnosed extrapulmonary TB patients = the mean of the proportion of undiagnosed smear-positive patients and the proportion of undiagnosed smear-negative patients = 0.195 (95%CI:0.151—0.236)

Assumption 5: The number of extrapulmonary TB patients diagnosed (Step 3) = 249,779 (95%CI:247,840—251,465)

Extrapulmonary TB Step 2 estimate = 249,779 / (1 – 0.195) = 310,284

Extrapulmonary TB Step 2 lower bound = 247,840 / (1 – 0.151) = 291,920

Extrapulmonary TB Step 2 upper bound = 251,465 / (1 – 0.236) = 329,142

Multidrug-resistant patients – Step 2:

Assumption 1: The WHO's estimate of the number of "MDR TB patients among all notified TB patients" in India is based on mycobacterial culture-based epidemiological studies performed at RNTCP sites that estimate the proportion MDR TB patients among both new and retreatment TB patients being evaluated at TB diagnostic centers. In 2013, the vast majority of RNTCP TB diagnostic centers did not have access to mycobacterial culture or Xpert® MTB/Rif testing. We therefore assume that the WHO's estimate of MDR TB patients among all notified TB patients

provides a relatively accurate estimate of the number of MDR TB patients reaching RNTCP TB diagnostic centers (Step 2), but it does not provide an accurate estimate of the number of MDR TB patients diagnosed (Step 3), since the majority of these MDR TB patients in 2013 were diagnosed using sputum smear or empirically and therefore may have been misclassified as being "new" or "retreatment" cases.

MDR TB Step 2 estimate from the 2014 WHO report [6] = 61,000 (95%CI:47,000—76,000)

Overall cascade (all forms of TB) – Step 2:

Assumption 1: For MDR TB patients, we assume that Gap 2—the gap between Step 2 and Step 3—consists of MDR TB patients who present to RNTCP TB diagnostic facilities but who are misclassified as either "new" or "retreatment" cases. Since we assume these MDR TB patients are misclassified (rather than lost to follow-up or other outcomes), they remain within the overall TB cascade in the new and retreatment TB categories. We therefore exclude Gap 2 for MDR TB patients from the overall TB cascade estimate for Step 2 so that these patients are not "double counted". We do this by adding the Step 3 estimate for MDR TB to the overall Step 2 combined estimate for all other forms of TB.

Assumption 2: MDR TB Step 3 estimate = 25,062 (95%CI:24,678—25,493)

Overall cascade estimate for Step 2 = Combined estimate for all forms of TB except MDR TB for Step 2 + MDR TB Step 3 estimate = 1,912,965 + 25,062 = 1,938,027

Overall cascade Step 2 lower bound = Combined lower bounds for all forms of TB except MDR TB for Step 2 + MDR TB Step 3 lower bound = 1,816,070 + 24,678 = 1,840,748

Overall cascade Step 2 upper bound = Combined upper bounds for all forms of TB except MDR TB for Step 2 + MDR TB Step 3 upper bound = 2,024,508 + 25,493 = 2,050,001

Gap 2: Number of TB patients evaluated at public sector TB diagnostic facilities who remain undiagnosed

New smear-positive patients – Gap 2:

Assumption 1: New smear-positive Step 2 = 730,108 (95%CI:726,428—733,826)

Assumption 2: New smear-positive Step 3 = 720,617 (no confidence interval, see below for how this was estimated)

New smear-positive Gap 2 estimate = 730,108 – 720,617 = 9,491

New smear-positive Gap 2 lower bound = 726,428 – 720,617 = 5,811

New smear-positive Gap 2 upper bound = 733,826 – 720,617 = 13,209

Retreatment smear-positive patients – Gap 2:

Assumption 1: Retreatment smear-positive Step 2 = 210,307 (95%CI:209,247—211,378)

Assumption 2: Retreatment smear-positive Step 3 = 207,573

Retreatment smear-positive Gap 2 estimate = 210,307 – 207,573 = 2,734

Retreatment smear-positive Gap 2 lower bound = 209,247 – 207,573 = 1,674

Retreatment smear-positive Gap 2 upper bound = 211,378 – 207,573 = 3,805

New smear-negative patients – Gap 2:

Assumption 1: New smear-negative Step 2 = 514,161 (95%CI:456,873—582,402)

Assumption 2: New smear-negative Step 3 = 320,982 (95%CI:318,491—323,148)

New smear-negative Gap 2 estimate = 514,161 – 320,982 = 193,179

New smear-negative Gap 2 lower bound = 456,873 – 323,148 = 133,725

New smear-negative Gap 2 upper bound = 582,402 – 318,491 = 263,911

Retreatment smear-negative patients – Gap 2:

Assumption 1: Retreatment smear-negative Step 2 = 148,104 (95%CI:131,602—167,760)

Assumption 2: Retreatment smear-negative Step 3 = 105,893 (95%CI:105,071—106,607)

Retreatment smear-negative Gap 2 estimate = 148,104 – 105,893 = 42,211

Retreatment smear-negative Gap 2 lower bound = 131,602 – 106,607 = 24,995

Retreatment smear-negative Gap 2 upper bound = 167,760 – 105,071 = 62,689

Extrapulmonary TB patients – Gap 2:

Assumption 1: Extrapulmonary TB Step 2 = 310,284 (95%CI:291,920—329,142)

Assumption 2: Extrapulmonary TB Step 3 = 249,779 (95%CI:247,840—251,465)

Extrapulmonary TB Gap 2 estimate = Extrapulmonary Step 2 estimate – Extrapulmonary Step 3 estimate = 310,284 – 249,779 = 60,505

Extrapulmonary TB Gap 2 lower bound = Extrapulmonary Step 2 lower bound – Extrapulmonary Step 3 upper bound = 291,920 – 251,465 = 40,455

Extrapulmonary TB Gap 2 upper bound = Extrapulmonary Step 2 upper bound – Extrapulmonary Step 3 lower bound = 329,142 – 247,840 = 81,302

Multidrug resistant patients – Gap 2:

Assumption 1: From the 2014 WHO Global TB report [6], the estimated number of true MDR TB patients who were evaluated at TB diagnostic facilities (Step 2) = 61,000 (95%CI:47,000—76,000)

Assumption 2: Number of MDR TB patients diagnosed at TB diagnostic facilities (Step 3) = 25,062 (95%CI:24,678—25,493)

MDR TB Gap 2 estimate = 61,000 – 25,062 = 35,938

MDR TB Gap 2 lower bound = 47,000 – 25,493 = 21,507

MDR TB Gap 2 upper bound = 76,000 – 24,678 = 51,322

Overall cascade (all forms of TB) – Gap 2:

Overall cascade Gap 2 estimate = Overall Step 2 estimate for all forms of TB – Overall Step 3 estimate for all forms of TB = 1,938,027 – 1,629,906 = 308,121

Overall cascade Gap 2 lower bound = Overall Step 2 lower bound for all forms of TB – Overall Step 3 upper bound for all forms of TB = 1,840,748 – 1,634,903 = 205,845

Overall cascade Gap 2 upper bound = Overall Step 2 upper bound for all forms of TB – Overall Step 3 lower bound for all forms of TB = 2,050,001 – 1,624,270 = 425,731

Step 3: Number of patients successfully diagnosed with TB at public sector diagnostic facilities

New smear-positive patients – Step 3:

Assumption 1: From the 2014 *TB India* report [7], the total number of smear-positive patients diagnosed with TB in 2013 = 928,190

Assumption 2: From the 2015 *TB India* report [8], the number of new smear-positive TB patients registered for treatment in 2013 (Step 4) = 615,609

Assumption 3: From the 2015 *TB India* report [8], the number of retreatment smear-positive TB patients registered for treatment in 2013 (Step 4) = 177,326

Assumption 4: The proportion of new-smear-positive positive TB patients registered for treatment among all smear-positive patients registered for treatment = 615,609 / (615,609 + 177,326) = 615,609 / 792,935 = 0.7763

Assumption 5: The proportion of new smear-positive patients diagnosed with TB among all smear-positive patients diagnosed with TB is the same as the proportion of new smear-positive positive TB patients registered for treatment among all smear-positive patients registered for treatment.

New smear-positive Step 3 estimate = (928,190)(0.7763) = 720,617

Retreatment smear-positive patients – Step 3:

Assumption 1: From the 2014 TB India report [7], the total number of smear-positive patients diagnosed with TB in 2013 = 928,190

Assumption 2: From the 2015 TB India report [8], the number of new smear-positive TB patients registered for treatment in 2013(Step 4) = 615,609

Assumption 3: From the 2015 TB India report [8], the number of retreatment smear-positive TB patients registered for treatment in 2013 (Step 4) = 177,326

Assumption 4: The proportion of retreatment smear-positive positive TB patients registered for treatment among all smear-positive patients registered for treatment = 177,326 / (615,609 + 177,326) = 177,326 / 792,935 = 0.2236

Assumption 5: The proportion of retreatment smear-positive patients diagnosed with TB among all smear-positive patients diagnosed with TB is the same as the proportion of retreatment smear-positive positive TB patients registered for treatment among all smear-positive patients registered for treatment.

New smear-positive Step 3 estimate = (928,190)(0.2236) = 207,573

New smear-negative patients – Step 3:

Assumption 1: The pretreatment loss to follow-up rate for smear-negative TB patients = 0.105 (95%CI:0.098—0.111)

Assumption 2: From the 2015 *TB India* report [8], the number of new smear-negative patients registered in TB treatment in 2013 (Step 4) = 287,279

New smear-negative Step 3 estimate = 287,279 / (1 – 0.105) = 320,982

New smear-negative Step 3 lower bound = 287,279 / (1 – 0.098) = 318,491

New smear-negative Step 3 upper bound = 287,279 / (1 – 0.111) = 323,148

Retreatment smear-negative patients – Step 3:

Assumption 1: The pretreatment loss to follow-up rate for retreatment smear-negative TB patients = 0.105 (95%CI:0.098—0.111)

Assumption 2: From the 2015 *TB India* report [8], the number of retreatment smear-negative patients registered in TB treatment in 2013 (Step 4) = 94,774

Retreatment smear-negative Step 3 estimate = 94,774 / (1 – 0.105) = 105,893

Retreatment smear-negative Step 3 lower bound = 94,774 / (1 – 0.098) = 105,071

Retreatment smear-negative Step 3 upper bound = 94,774 / (1 – 0.111) = 106,607

Extrapulmonary TB patients – Step 3:

Assumption 1: The pretreatment loss to follow-up rate for extrapulmonary TB patients = 0.105 (95%CI:0.098—0.111)

Assumption 2: From the 2015 *TB India* report [8], the number of extrapulmonary TB patients registered in TB treatment in 2013 (Step 4) = 223,552

Extrapulmonary TB Step 3 estimate = 223,552 / (1 – 0.105) = 249,779

Extrapulmonary TB Step 3 lower bound = 223,552 / (1 – 0.098) = 247,840

Extrapulmonary TB Step 3 upper bound = 223,552 / (1 – 0.111) = 251,465

MDR TB patients:

Assumption 1: The pretreatment loss to follow-up rate for MDR TB patients based on a pooled prevalence of two studies [9, 10] = 0.230 (95%CI: 0.218-0.243)

Assumption 2: From the 2016 *TB India* report [11], the number of MDR TB patients registered in treatment in 2013 = 19,298

MDR TB Step 3 estimate = 19,298 / (1 – 0.23) = 25,062

MDR TB Step 3 lower bound = 19,298 / (1 – 0.218) = 24,678

MDR TB Step 3 upper bound = 19,298 / (1 – 0.243) = 25,493

Overall cascade (all forms of TB) – Step 3:

Overall cascade estimate for Step 3 = Combined Step 3 estimates for all forms of TB = 1,629,906

Overall cascade Step 3 lower bound = Combined Step 3 lower bounds for all forms of TB = 1,624,270

Overall cascade Step 3 upper bound = Combined Step 3 upper bounds for all forms of TB = 1,634,903

Gap 3: Number of patients diagnosed at public sector diagnostic facilities who are not registered for TB treatment (i.e., "pretreatment loss to follow-up" or "initial default")

New smear-positive patients – Gap 3:

Assumption 1: The number of new smear-positive patients diagnosed in 2013 (Step 3) = 720,617

Assumption 2: From the 2015 TB India report [8], the number of new smear-positive patients registered in TB treatment in 2013 (Step 4) = 615,609

New smear-positive Gap 3 estimate = 720,617 – 615,609 = 105,008 (no confidence interval as both values are extracted from the *TB India* reports)

Retreatment smear-positive patients – Gap 3:

Assumption 1: The number of retreatment smear-positive patients diagnosed in 2013 (Step 3) = 207,573

Assumption 2: From the 2015 *TB India* report [8], the number of retreatment smear-positive patients registered in TB treatment in 2013 (Step 4) = 177,326

Retreatment smear-positive Gap 3 estimate = 207,573 – 177,326 = 30,247 (no confidence interval as both values are precise and extracted from the *TB India* reports)

New smear-negative patients – Gap 3:

Assumption 1: The number of new smear-negative patients diagnosed in 2013 (Step 3) = 320,982 (95%CI:318,491—323,148)

Assumption 2: From the 2015 *TB India* report [8], the number of new smear-negative patients registered in TB treatment in 2013 (Step 4) = 287,279

New smear-negative Gap 3 estimate = 320,982 – 287,279 = 33,703

New smear-negative Gap 3 lower bound = 318,491 – 287,279 = 31,212

New smear-negative Gap 3 upper bound = 323,148 – 287,279 = 35,869

Retreatment smear-negative patients – Gap 3:

Assumption 1: The number of retreatment smear-negative patients diagnosed in 2013 (Step 3) = 105,893 (95%CI:105,071—106,607)

Assumption 2: From the 2014 *TB India* report [7], the number of retreatment smear-negative patients registered in treatment in 2013 (Step 4) = 94,774

Retreatment smear-negative Gap 3 estimate = 105,893 – 94,774 = 11,119

Retreatment smear-negative Gap 3 lower bound = 105,071 – 94,774 = 10,297

Retreatment smear-negative Gap 3 upper bound = 106,607 – 94,774 = 11,833

Extrapulmonary TB patients – Gap 3:

Assumption 1: The number of extrapulmonary TB patients diagnosed in 2013 (Step 3) = 249,779 (95%CI:247,840—251,465)

Assumption 2: From the 2015 *TB India* report [8], the number of extrapulmonary TB patients registered in treatment in 2013 (Step 4) = 223,552

Extrapulmonary TB Gap 3 estimate = 249,779 – 223,552 = 26,227

Extrapulmonary TB Gap 3 lower bound = 247,840 – 223,552 = 24,288

Extrapulmonary TB Gap 3 upper bound = 251,465 – 223,552 = 27,913

MDR TB patients – Gap 3:

Assumption 1: The number of MDR TB patients diagnosed in 2013 (Step 3) = 25,062 (95%CI:24,678—25,493)

Assumption 2: From the 2016 *TB India* report [11], the number of MDR TB patients registered in treatment in 2013 (Step 4) = 19,298

MDR TB Gap 3 estimate = 25,062 – 19,298 = 5,764

MDR TB Gap 3 lower bound = 24,678 – 19,298 = 5,380

MDR TB Gap 3 upper bound = 25,493 – 19,298 = 6,195

Overall cascade (all forms of TB) – Gap 3:

Overall cascade Gap 3 estimate = Overall cascade Step 3 estimate – Overall cascade Step 4 estimate = 1,629,906 – 1,417,838 = 212,068

Overall cascade Gap 3 lower bound = Overall cascade Step 3 lower bound – Overall cascade Step 4 estimate [no confidence interval for Step 4] = 1,624,270 – 1,417,838 = 206,432

Overall cascade Gap 3 upper bound = Overall cascade Step 3 upper bound – Overall cascade Step 4 estimate [no confidence interval for Step 4] = 1,634,903 – 1,417,838 = 217,065

Step 4: Number of patients registered for TB treatment in the public sector

The numbers of patients registered in TB treatment in 2013 for all forms of TB (new smear-positive, retreatment smear-positive, new smear-negative, retreatment smear-negative, extrapulmonary, and MDR TB) were extracted from the 2015 and 2016 *TB India* reports [8, 11].

New smear-positive patients – Step 4:

New smear-positive Step 4 estimate for 2013 from the 2015 TB India report [8] = 615,609

Retreatment smear-positive patients – Step 4:

Retreatment smear-positive Step 4 estimate for 2013 from the 2015 TB India report [8] = 177,326

New smear-negative patients – Step 4:

New smear-negative Step 4 estimate for 2013 from the 2015 TB India report [8] = 287,279

Retreatment smear-negative patients – Step 4:

Retreatment smear-negative Step 4 estimate for 2013 from the 2015 TB India report [8] = 94,774

Extrapulmonary TB patients – Step 4:

Extrapulmonary TB Step 4 estimate for 2013 from the 2015 TB India report [8] = 223,552

MDR TB patients – Step 4:

MDR TB Step 4 estimate for 2013 from the 2016 TB India report [11]= 19,298

Overall cascade (all forms of TB) – Step 4:

Overall cascade Step 4 estimate = Combined Step 4 estimates for all forms of TB = 1,417,838

Gap 4a/4b: Number of patients who fail therapy, are lost to follow-up, or die during TB treatment

New smear-positive patients – Gap 4a/4b:

New smear-positive Gap 4a/4b estimate = New smear-positive Step 4 estimate – New smear-positive Step 5 estimate = 615,609 – 541,736 = 73,873

Retreatment smear-positive patients – Gap 4a/4b:

Retreatment smear-positive Gap 4a/4b estimate = Retreatment smear-positive Step 4 estimate – Retreatment smear-positive Step 5 estimate = 177,326 – 125,901 = 51,425

New smear-negative patients – Gap 4a/4b:

New smear-negative Gap 4a/4b estimate = New smear-negative Step 4 estimate – New smear-negative Step 5 estimate = 287,279 – 258,551 = 28,728

Retreatment smear-negative patients – Gap 4a/4b:

Retreatment smear-negative Gap 4a/4b estimate = Retreatment smear-negative Step 4 estimate – Retreatment smear-negative Step 5 estimate = 94,774 – 78,852 = 15,922

Retreatment smear-negative Gap 4a/4b lower bound = Retreatment smear-negative Step 4 estimate [no confidence interval] – Retreatment smear-negative Step 5 upper bound = 94,774 – 80,747 = 14,027

Retreatment smear-negative Gap 4a/4b upper bound = Retreatment smear-negative Step 4 estimate [no confidence interval] – Retreatment smear-negative Step 5 lower bound = 94,774 – 76,767 = 18,007

Extrapulmonary TB patients – Gap 4a/4b:

Extrapulmonary TB Gap 4a/4b estimate = Extrapulmonary TB Step 4 estimate – Extrapulmonary TB Step 5 estimate = 15,649

MDR TB patients – Gap 4a/4b:

MDR TB Gap 4a/4b estimate = MDR TB Step 4 estimate – MDR TB Step 5 estimate = 10,477

Overall cascade (all forms of TB) – Gap 4a/4b:

Overall cascade Gap 4a/4b estimate = Overall Step 4 estimate for all forms of TB – Overall Step 5 estimate for all forms of TB = 196,074

Overall cascade Gap 4a/4b lower bound = Overall Step 4 lower bound for all forms of TB – Overall Step 5 upper found for all forms of TB = 194,179

Overall cascade Gap 4a/4b upper bound = Overall Step 4 upper bound for all forms of TB – Overall Step 5 lower bound for all forms of TB = 198,159

Step 5: Number of TB patients who achieve treatment completion or cure

The numbers of patients who achieved treatment completion or cure in 2013 for all forms of TB except retreatment smear-negative TB were extracted from the 2015 and 2016 *TB India* reports [8, 11]. The number of retreatment smear-negative patients who achieved treatment completion or cure was estimated as described below.

New smear-positive patients – Step 5:

New smear-positive Step 5 estimate for 2013 from the 2015 TB India report [8] = 541,736

Retreatment smear-positive patients – Step 5:

Retreatment smear-positive Step 5 estimate for 2013 from the 2015 TB India report [8] = 125,901

New smear-negative patients – Step 5:

New smear-negative Step 5 estimate for 2013 from the 2015 TB India report [8] = 258,551

Retreatment smear-negative TB patients – Step 5:

Assumption 1: Based on a study of treatment outcomes among retreatment "others" (smear-negative) patients [12], we estimate that the proportion of retreatment smear-negative patients who achieve treatment completion = 0.832 (95%CI:0.810—0.852).

Assumption 2: The number of retreatment smear-negative patients registered in treatment in 2013 = 94,774

Retreatment smear-negative Step 5 estimate = 94,774 X 0.832 = 78,852

Retreatment smear-negative Step 5 lower bound = 94,774 X 0.810 = 76,767

Retreatment smear-negative Step 5 upper bound = 94,774 X 0.852 = 80,747

Extrapulmonary TB patients – Step 5:

Extrapulmonary TB Step 5 estimate for 2013 from the 2015 TB India report [8] = 207,903

MDR TB patients – Step 5:

MDR TB Step 5 estimate for 2013 from the 2016 TB India report [11] = 8,821

Overall cascade (all forms of TB) – Step 5:

Overall cascade Step 5 estimate = Combined Step 5 estimates for all forms of TB = 1,221,764

Overall cascade Step 5 lower bound = Combined Step 5 estimates for all forms of TB other than retreatment smear-negative TB + Step 5 lower bound for retreatment smear-negative TB = 1,219,679

Overall cascade Step 5 upper bound = Combined Step 5 estimates for all forms of TB other than retreatment smear-negative TB + Step 5 upper bound for retreatment smear-negative TB = 1,223,659

Gap 5: Number of TB patients who experience death or TB recurrence in the first 12-24 months after completing therapy

New smear-positive TB patients – Gap 5:

Assumption 1: The post-treatment TB recurrence rate within 12-24 months of treatment completion for new smear-positive patients based on pooled prevalence of two studies [13, 14] = 0.162 (95%CI:0.142—0.185)

Assumption 2: From the 2015 TB India report, the number of smear-positive patients who achieved treatment completion or cure in 2013 = 541,736

New smear-positive Gap 5 estimate = 541,736 X 0.162 = 87,761

New smear-positive Gap 5 lower bound = 541,736 X 0.142 = 76,927

New smear-positive Gap 5 upper bound = 541,736 X 0.185 = 100,221

Retreatment smear-positive TB patients – Gap 5:

Assumption 1: The post-treatment TB recurrence rate within 12-24 months of treatment completion for retreatment smear-positive patients based on an estimate from a study [13] = 0.273 (95%CI:0.191—0.374)

Assumption 2: From the 2015 TB India report, the number of retreatment smear-positive patients who achieved treatment completion or cure in 2013 = 125,901

Retreatment smear-positive Gap 5 estimate = 125,901 X 0.273 = 34,371

Retreatment smear-positive Gap 5 lower bound = 125,901 X 0.191 = 24,047

Retreatment smear-positive Gap 5 upper bound = 125,901 X 0.374 = 47,087

New smear-negative TB patients – Gap 5:

Assumption 1: The post-treatment TB recurrence rate within 12-24 months of treatment completion for new smear-negative patients based on pooled prevalence of four studies [15-18] = 0.088 (95%CI:0.069—0.111)

Assumption 2: From the 2015 TB India report, the number of new smear-negative patients who achieved treatment completion or cure in 2013 = 258,551

New smear-negative Gap 5 estimate = 258,551 X 0.088 = 22,752

New smear-negative Gap 5 lower bound = 258,551 X 0.069 = 17,840

New smear-negative Gap 5 upper bound = 258,551 X 0.111 = 28,699

Retreatment smear-negative TB patients – Gap 5:

Assumption 1: The post-treatment TB recurrence rate within 12-24 months of treatment completion for retreatment smear-negative patients based on pooled prevalence of four studies [15-18] = 0.088 (95%CI:0.069—0.111)

Assumption 2: From the 2015 TB India report, the number of retreatment smear-negative patients who achieved treatment completion or cure in 2013 = 78,852 (95%CI:76,767—80,747)

Assumption 3: As calculated below, the number of retreatment smear-negative patients who experience post-treatment TB recurrence or death = 71,913 (95%CI:68,246—75,175)

Retreatment smear-negative Gap 5 estimate = 78,852 – 71,913 = 6,939

Retreatment smear-negative Gap 5 lower bound = 76,767 – 75,175 = 1,592

Retreatment smear-negative Gap 5 upper bound = 80,747 – 68,246 = 12,501

Extrapulmonary TB patients – Gap 5:

Assumption 1: The post-treatment TB recurrence rate within 12-24 months of treatment completion for extrapulmonary TB patients based on pooled prevalence of four studies [15-18] = 0.088 (95%CI:0.069—0.111)

Assumption 2: The number of extrapulmonary TB patients registered in TB treatment in 2013 who completed a full course of therapy from the 2015 *TB India* report [11] = 207,903

Extrapulmonary TB Gap 5 estimate = 207,903 X 0.088 = 18,295

Extrapulmonary TB Gap 5 lower bound = 207,903 X 0.069 = 14,345

Extrapulmonary TB Gap 5 upper bound = 207,903 X 0.111 = 23,077

MDR TB patients – Gap 5:

Assumption 1: The post-treatment completion TB recurrence rate within 12-24 months of treatment completion is at least as high as the TB recurrence rate for retreatment smear-positive patients = 0.273 (95%CI:0.191—0.374)

Assumption 2: The number of MDR TB patients registered in TB treatment in 2013 who completed a full course of therapy from the 2016 *TB India* report [11] = 8,821

MDR TB Gap 5 estimate = 8,821 X 0.273 = 2,408

MDR TB Gap 5 lower bound = 8,821 X 0.191 = 1,685

MDR TB Gap 5 upper bound = 8,821 X 0.374 = 3,299

Overall TB cascade – Gap 5:

Overall cascade Gap 5 estimate = Combined Gap 5 estimates for all forms of TB = 172,527

Overall cascade Gap 5 lower bound = Combined Gap 5 lower bounds for all forms of TB = 136,436

Overall cascade Gap 5 upper bound = Combined Gap 5 upper bounds for all forms of TB = 214,884

Step 6: Number of TB patients who are alive and TB recurrence-free 12-24 months after completing therapy

New smear-positive TB patients – Step 6:

Assumption 1: The post-treatment TB recurrence rate within 12-24 months of treatment completion for new smear-positive patients based on pooled prevalence of two studies [13, 14] = 0.162 (95%CI:0.142—0.185)

Assumption 2: From the 2015 TB India report, the number of smear-positive patients who achieved treatment completion or cure in 2013 = 541,736

New smear-positive Step 6 estimate = 541,736 X (1 – 0.162) = 453,975

New smear-positive Step 6 lower bound = 541,736 X (1 – 0.185) = 441,515

New smear-positive Step 6 upper bound = 541,736 X (1 – 0.142) = 464,809

Retreatment smear-positive TB patients – Step 6:

Assumption 1: The post-treatment TB recurrence rate within 12-24 months of treatment completion for retreatment smear-positive patients based on an estimate from a study [13] = 0.273 (95%CI:0.191—0.374)

Assumption 2: From the 2015 TB India report, the number of retreatment smear-positive patients who achieved treatment completion or cure in 2013 = 125,901

Retreatment smear-positive Step 6 estimate = 125,901 X (1 – 0.273) = 91,530

Retreatment smear-positive Step 6 lower bound = 125,901 X (1 – 0.374) = 78,814

Retreatment smear-positive Step 6 upper bound = 125,901 X (1 – 0.191) = 101,854

New smear-negative TB patients – Step 6:

Assumption 1: The post-treatment TB recurrence rate within 12-24 months of treatment completion for new smear-negative patients based on pooled prevalence of four studies [15-18] = 0.088 (95%CI:0.069—0.111)

Assumption 2: From the 2015 TB India report, the number of new smear-negative patients who achieved treatment completion or cure in 2013 = 258,551

New smear-negative Step 6 estimate = 258,551 X (1 – 0.088) = 235,799

New smear-negative Step 6 lower bound = 258,551 X (1 – 0.111) = 229,852

New smear-negative Step 6 upper bound = 258,551 X (1 – 0.069) = 240,711

Retreatment smear-negative TB patients – Step 6:

Assumption 1: The post-treatment TB recurrence rate within 12-24 months of treatment completion for retreatment smear-negative patients based on pooled prevalence of four studies [15-18] = 0.088 (95%CI:0.069—0.111)

Assumption 2: From the 2015 TB India report, the number of retreatment smear-negative patients who achieved treatment completion or cure in 2013 = 78,852 (95%CI:76,767—80,747)

Retreatment smear-negative Step 6 estimate = 78,852 X (1 – 0.088) = 71,913

Retreatment smear-negative Step 6 lower bound = 76,767 X (1 – 0.111) = 68,246

Retreatment smear-negative Step 6 upper bound = 80,747 X (1 – 0.069) = 75,175

Extrapulmonary TB patients – Step 6:

Assumption 1: The post-treatment TB recurrence rate within 12-24 months of treatment completion for extrapulmonary TB patients based on pooled prevalence of four studies [15-18] = 0.088 (95%CI:0.069—0.111)

Assumption 2: The number of extrapulmonary TB patients registered in TB treatment in 2013 who completed a full course of therapy from the 2015 *TB India* report [11] = 207,903

Extrapulmonary TB Step 6 estimate = 207,903 X (1 – 0.088) = 189,608

Extrapulmonary TB Step 6 lower bound = 207,903 X (1 – 0.111) = 184,826

Extrapulmonary TB Step 6 upper bound = 207,903 X (1 – 0.069) = 193,558

MDR TB patients – Step 6:

Assumption 1: The post-treatment completion TB recurrence rate within 12-24 months of treatment completion is at least as high as the TB recurrence rate for retreatment smear-positive patients = 0.273 (95%CI:0.191—0.374)

Assumption 2: The number of MDR TB patients registered in TB treatment in 2013 who completed a full course of therapy from the 2016 *TB India* report [11] = 8,821

MDR TB Step 6 estimate = 8,821 X (1 – 0.273) = 6,413

MDR TB Step 6 lower bound = 8,821 X (1 – 0.374) = 5,522

MDR TB Step 6 upper bound = 8,821 X (1 – 0.191) = 7,136

Overall TB cascade – Step 6:

Overall cascade Step 6 estimate = Combined Step 6 estimates for all forms of TB = 1,049,237

Overall cascade Step 6 lower bound = Combined Step 6 lower bounds for all forms of TB = 1,008,775

Overall cascade Step 6 upper bound = Combined Step 6 upper bounds for all forms of TB = 1,083,243

References

1. World Health Organization (WHO). Global tuberculosis report. Geneva: WHO, 2015 Contract No.: WHO/HTM/TB/2015.22.
2. World Health Organization (WHO). WHO TB burden estimates [dataset]. 2016 Mar 22 [cited 2016 June 20]. WHO. Available from: https://extranet.who.int/tme/generateCSV.asp?ds=estimates
3. Mase SR, Ramsay A, Ng V, Henry M, Hopewell PC, Cunningham J, et al. Yield of serial sputum specimen examinations in the diagnosis of pulmonary tuberculosis: a systematic review. Int J Tuberc Lung Dis. 2007;11(5):485-95. PMID: 17439669.
4. Sachdeva KS, Raizada N, Sreenivas A, Van't Hoog AH, van den Hof S, Dewan PK, et al. Use of Xpert MTB/RIF in Decentralized Public Health Settings and Its Effect on Pulmonary TB and DR-TB Case Finding in India. PLoS One. 2015;10(5):e0126065. doi: 10.1371/journal.pone.0126065. PMID: 25996389.
5. Steingart KR, Schiller I, Horne DJ, Pai M, Boehme CC, Dendukuri N. Xpert(R) MTB/RIF assay for pulmonary tuberculosis and rifampicin resistance in adults. Cochrane Database Syst Rev. 2014;1:Cd009593. doi: 10.1002/14651858.CD009593.pub3. PMID: 24448973.
6. World Health Organization (WHO). Global tuberculosis report. Geneva: World Health Organization, 2014 Contract No.: WHO/HTM/TB/2014.08.
7. Central TB Division. TB India 2014: Revised National TB Control Programme annual status report. New Delhi: Ministry of Health and Family Welfare, 2014.
8. Central TB Division. TB India 2015: Revised National TB Control Programme annual status report. New Delhi: Ministry of Health and Family Welfare, 2015.
9. Chadha SS, Sharath BN, Reddy K, Jaju J, Vishnu PH, Rao S, et al. Operational challenges in diagnosing multi-drug resistant TB and initiating treatment in Andhra Pradesh, India. PLoS One. 2011;6(11):e26659. doi: 10.1371/journal.pone.0026659. PMID: 22073182.
10. Shringarpure KS, Isaakidis P, Sagili KD, Baxi RK. Loss-To-Follow-Up on Multidrug Resistant Tuberculosis Treatment in Gujarat, India: The WHEN and WHO of It. PLoS One. 2015;10(7):e0132543. doi: 10.1371/journal.pone.0132543. PMID: 26167891.
11. Central TB Division. TB India 2016: Revised National TB Control Programme annual status report. New Delhi: Ministry of Health and Family Welfare, 2016.
12. Srinath S, Sharath B, Santosha K, Chadha SS, Roopa S, Chander K, et al. Tuberculosis 'retreatment others': profile and treatment outcomes in the state of Andhra Pradesh, India. Int J Tuberc Lung Dis. 2011;15(1):105-9. PMID: 21276305.
13. Sadacharam K, Gopi PG, Chandrasekaran V, Eusuff SI, Subramani R, Santha T, et al. Status of smear-positive TB patients at 2-3 years after initiation of treatment under a DOTS programme. Indian J Tuberc. 2007;54(4):199-203. PMID: 18072535.
14. Thomas A, Gopi PG, Santha T, Chandrasekaran V, Subramani R, Selvakumar N, et al. Predictors of relapse among pulmonary tuberculosis patients treated in a DOTS programme in South India. Int J Tuberc Lung Dis. 2005;9(5):556-61. PMID: 15875929.
15. Tripathy S, Anand A, Inamdar V, Manoj MM, Khillare KM, Datye AS, et al. Clinical response of newly diagnosed HIV seropositive & seronegative pulmonary tuberculosis patients with the RNTCP Short Course regimen in Pune, India. Indian J Med Res. 2011;133:521-8. PMID: 21623038.
16. Dandekar RH, Jagannath VD. The fate of tuberculosis cases after two years of DOTS chemotherapy in Aurangabad city, Maharasthra. Natl J Community Med. 2014;5(2):174-8.
17. Prasad R, Verma SK, Shrivastava P, Kant S, Kushwaha RA, Kumar S. A follow up study on Revised National Tuberculosis Control Programme (RNTCP): results from a single centre study. Lung India. 2008;25(4):142-4. doi: 10.4103/0970-2113.45277. PMID: 21264079.
18. Vashishtha R, Mohan K, Singh B, Devarapu SK, Sreenivas V, Ranjan S, et al. Efficacy and safety of thrice weekly DOTS in tuberculosis patients with and without HIV co-infection: an

observational study. BMC Infect Dis. 2013;13:468. doi: 10.1186/1471-2334-13-468. PMID: 24099345.
